# Synthesis and Stereochemical Assignment of Diastereomeric Steroidal Spiro-1,2,4-trioxolanes with Sitostanone Motif Using PLS2 Chemometric Analysis of DFT-Calculated and Experimental ^13^C NMR Data †

**DOI:** 10.3390/molecules31173105

**Published:** 2026-09-04

**Authors:** Alexander Lobov, Anastasiya Kerimova, Irina Smirnova, Dmitriy Polovyanenko, Irina Bagryanskaya, Oxana Kazakova

**Affiliations:** 1 Ufa Institute of Chemistry of the Ufa Federal Research Centre of the Russian Academy of Sciences, 71, pr. Oktyabrya, 450054 Ufa, Russia; lobovan@anrb.ru (A.L.); ana.orgchem@gmail.com (A.K.); si8081@yandex.ru (I.S.); 2 N. N. Vorozhtsov Novosibirsk Institute of Organic Chemistry SB RAS, 630090 Novosibirsk, Russia; dpolo@nioch.nsc.ru (D.P.); bagryan@nioch.nsc.ru (I.B.)

**Keywords:** 3-*spiro*-1,2,4-trioxolane, steroids, sitostanone, Griesbaum co-ozonolysis, X-ray analysis, high-resolution NMR, DFT, chemometrics

## Abstract

The synthesis and multi-step spectroscopic characterization of diastereomeric 3-spiro-1,2,4-trioxolanes obtained by Griesbaum co-ozonolysis of sitostanone O-methyl oxime with two types of fluorinated ketones are reported. The reaction furnished four possible diastereomers that differ in the α/β orientation of the peroxide bridge relative to the steroid A-ring and in the *syn*/*anti* orientation of the trifluoromethyl group at C5′. A chemometric PLS2 approach, linking experimental and DFT-calculated ^13^C chemical shifts, was used for the objective stereochemical assignment of each diastereomer in the inseparable mixtures. Single-crystal X-ray analysis of the isolated 3*R*,5′*R* and 3*R*,5′*S* stereoisomeric pair (α-*anti* and α-*syn* ozonides) provided unambiguous absolute configurations that validated the stereochemical assignments obtained by the combined NMR/DFT/PLS2 approach. This integrated synthetic, crystallographic, spectroscopic, and chemometric methodology provides reliable configurational assignment in complex spiro-peroxide mixtures and expands the analytical toolkit for such systems.

## 1. Introduction

Natural compounds are important sources of biologically active substances with relatively low toxicity and high selectivity. Among them, steroids constitute a large class of lipophilic molecules with a tetracyclic cyclopentanoperhydrophenanthrene core, widely distributed in plants, animals, and fungi [1]. Due to their structural and functional diversity, steroids are employed in pharmacology as anti-inflammatory and immunosuppressive agents, anabolic drugs, hormonal contraceptives, and anticancer therapeutics [2], as well as in materials science for the development of liposomal drug delivery systems and biocompatible polymers [3].

Steroids are classified into sterols (cholesterol, phytosterols), bile acids, steroid hormones, cardiac glycosides, saponins, and steroidal alkaloids. Of particular interest are phytosterols known as plant analogs of cholesterol, such as β-sitosterol and stigmasterol, which differ in the structure of the side chain and the functional group at C3, while their fully saturated analogs are referred to as stanols [4].

**Figure 1:**
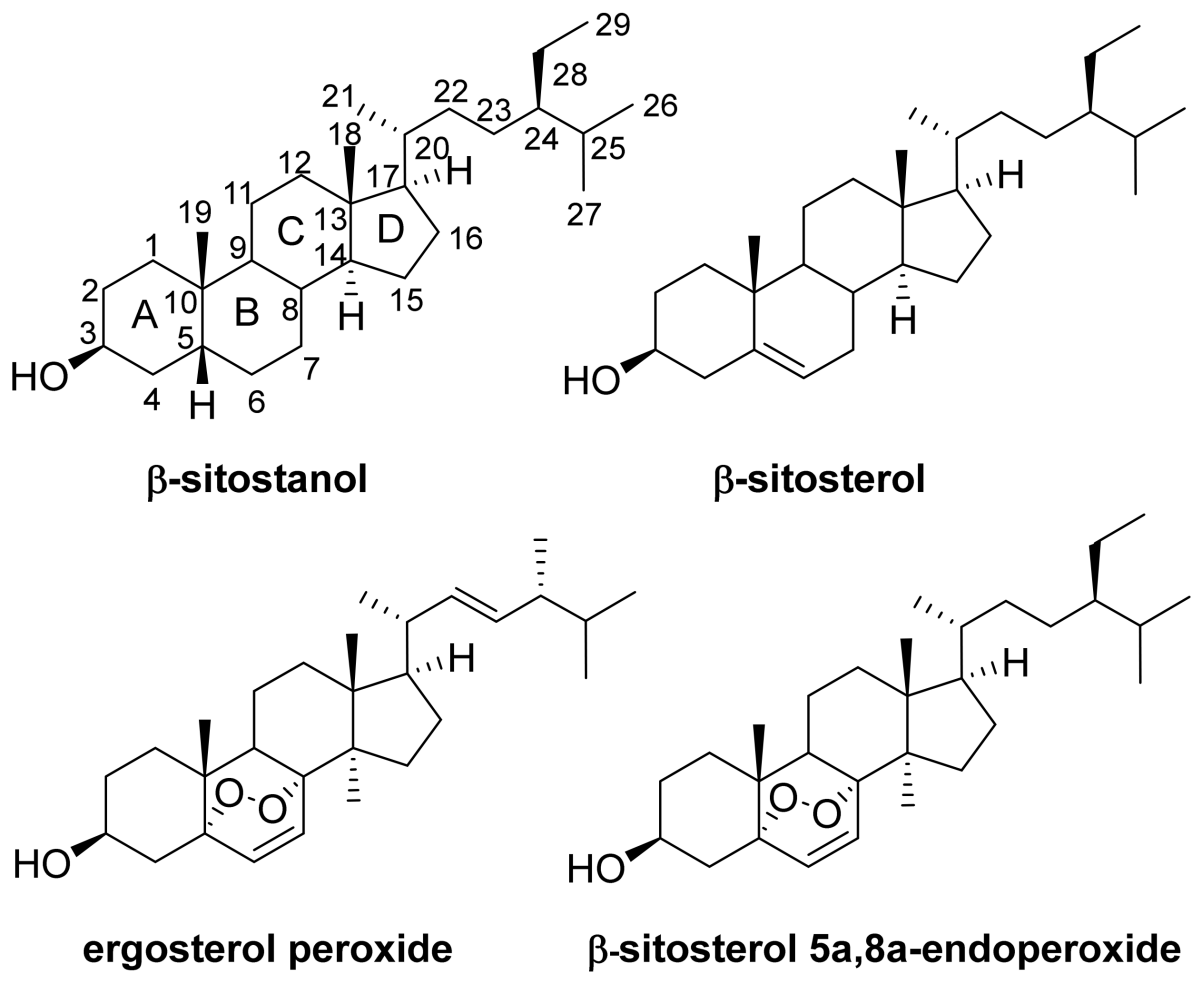
The structures of β-sitostanol, β-sitosterol, ergosterol peroxide and β-sitosterol 5a,8a-endoperoxide.

Two representative phytosterols are β-sitosterol and its saturated analog sitostanol (3β,5α-stigmastan-3-ol, Figure 1). In natural sources, sitostanol occurs in relatively low amounts and is found in various plant products, including vegetable oils, cereals, nuts, vegetables, and legumes. Industrially, an important source of phytosterols is wood processing by-products, particularly tall oil, as well as plant oils that are rich in β-sitosterol [5]. In plant materials, it is found almost exclusively as a ferulate or *p*-coumarate ester [6].

The interest in these compounds is driven by their pronounced biological effects [7]. In particular, sitostanol reduces intestinal cholesterol absorption by 20–30%, leading to a decrease in low-density lipoprotein levels by approximately 10–15% [8], and is used as a component of certain margarines [9]. In vitro studies for anticancer activity summarized in the literature commonly used β-sitosterol concentrations in the low-to-moderate micromolar range, for example, 8–16 μM in HT-29 cells, 10–16 μM in breast cancer cells, and 25–100 μM in PC-3 prostate cancer cells, whereas animal experiments employed doses such as 10–20 or 20 mg/kg, depending on the disease model and route of administration [10,11,12,13]. With regard to the mechanism of action, β-sitosterol and β-sitostanol have been reported to exhibit anticancer activity through the induction of apoptosis, cell-cycle arrest, and inhibition of metastatic processes. In silico studies have further proposed estrogen receptor β and caspase-3 as potential molecular targets [14,15,16].

However, sitostanol and β-sitosterol have low water solubility due to their lipophilic tetracyclic structure, which limits in vitro and in vivo administration, and their pharmaceutical development requires an appropriate formulation. To improve bioavailability, researchers utilize specific derivatives like fatty acid esters (e.g., sitostanol stearate, β-sitosterol β-D-glucoside) [17] and delivery systems like liposomes, micelles, or nanoemulsions [18,19,20]. Dietary or supplemental intake is typically studied in ranges aimed at cholesterol-lowering efficacy. Several dosage forms containing β-sitosterol or phytosterol mixtures have been described, including capsules, tablets, oral suspensions, and phytosterol-enriched food products [21]. For the relief of symptoms associated with benign prostatic hyperplasia, β-sitosterol has commonly been administered orally at doses of 60–130 mg/day in divided doses [22]. For cholesterol-lowering applications, phytosterols or phytostanols are generally used at approximately 1.8–2.6 g/day, usually with meals [23].

In recent years, considerable attention has been devoted to the synthesis of biologically active stable peroxides holding 1,2,4,5-tetraoxane [24,25], 1,2,4-trioxolane [26,27,28,29] as well as 1,2,4-dioxazolidine [30] units connected with diterpene-, triterpene- or cholane-type scaffolds. Peroxide-containing steroids constitute a promising yet insufficiently explored class of bioactive compounds. In particular, spirocyclic peroxides incorporating bile acid scaffolds, such as deoxycholic and lithocholic acids, have attracted attention because of their diverse pharmacological potential. Functionalization at C-3 of cholic acid derivatives with spiro-peroxide fragments has provided 1,2,3,4-tetraoxanes [31] as well as 1,2,4-trioxanes [32], which displayed antimalarial, antiproliferative, antibacterial, and other biological activities [20,33]. Notably, 3′-trifluoromethylated spiro-1,2,4-trioxolanes derived from deoxycholic acid demonstrated in vitro antimalarial activity against both chloroquine-sensitive T96 and chloroquine-resistant K1 strains of *Plasmodium falciparum* [27]. Lithocholic acid-based spiro-1,2,4-trioxolanes have also shown antiviral potential. In particular, N-cyclohexyl-3,3′-spiro-[5′-trifluoromethyl-5′-phenyl-1,2,4-trioxolano-3-yl]-5β-cholan-24-amide inhibited the replication of the influenza A/Puerto Rico/8/34 (H1N1) virus in vitro, with an IC_50_ of 12.5 μM and a selectivity index (SI) of 37 [34]. These findings support the potential of bile acid-derived steroidal peroxides as a platform for the development of novel biologically active agents.

1,2,4-Trioxolanes were obtained by the Griesbaum co-ozonolysis method as four [27], two [28] or, as demonstrated recently, one [29] diastereoisomer. In the present study, sitostanone was introduced in the form of O-methyl oxime for Griesbaum co-ozonolysis using fluorinated ketones as carbonyl components. To the best of our knowledge, spiro-derivatives of phytostanols remain largely unexplored; among them, the most closed analogs with peroxy-groups could be named as ergosterol peroxide and β-sitosterol 5a,8a-endoperoxide (Figure 1) [35,36].

As recently highlighted in a review dedicated to the Griesbaum co-ozonolysis reaction [37], stereochemical analysis of the resulting diastereomeric mixtures remains a key challenge, and complete separation of such mixtures by chromatographic methods is often unattainable. It was mentioned that unambiguous assignment of NMR signals to each diastereomer in the mixture is critically important for understanding the stereochemical outcome of the reaction and for the subsequent targeted synthesis of desired isomers.

The diastereoselectivity of 1,2,4-trioxolane formation in the Griesbaum co-ozonolysis of *O*-methyl 2-adamantanone oxime and 4-substituted cyclohexanones led predominantly to single tetrasubstituted ozonides, with the major isomer assigned a *cis* configuration, and is primarily governed by steric and conformational factors [38]. A similar highly selective outcome with single diastereoisomer was observed in the co-ozonolysis of methyl lithocholate 3-methoxy-oxime with trifluoromethylcyclohexanone [29]. In contrast to the application of 4-substituted cyclohexanones, the use of CF_3_-containing acyclic ketones, such as trifluoroacetone, typically results in reduced diastereoselectivity. Compared with the well-defined stereochemical outcome for adamantanone and 4-substituted cyclohexanone substrates, steroid and triterpene systems show a broader product distribution that reflects the greater conformational complexity of these natural-product frameworks.

In the current literature, several approaches have been developed for stereochemical analysis based on comparison of calculated and experimental NMR chemical shifts. One of the earliest is the CP3 parameter, introduced by Smith and Goodman in 2009 for comparing two sets of experimental data with two possible diastereomeric structures [39]. This method is based on comparison of chemical shift differences, thereby minimizing systematic errors in the calculations. Later, the same authors developed DP4, a probabilistic method designed for cases where only one set of experimental data and multiple possible stereoisomers are available [40]. In 2015, Sarotti and co-workers introduced an improved version, DP4+, which incorporates both scaled and unscaled data as well as higher levels of theory for NMR calculations [41]. Subsequent modifications include J-DP4, which takes spin–spin coupling constants into account [42], and DP4-AI, which automates data processing using machine learning [43].

For cases in which multiple experimental datasets are available, the MAEΔΔδ method was developed by Lauro and co-workers [44]. This approach performs unsupervised matching of experimental and calculated datasets by exhaustive permutation of all possible combinations and selection of the one that gives the minimum mean absolute deviation of chemical shift differences. A similar permutation-based approach, using deviations of chemical shifts from the mean value for each atom, was proposed by Boratyński for the analysis of four diastereomers [45].

The Griesbaum co-ozonolysis of sitostanone *O*-methyl oxime with two acyclic fluorinated ketones as carbonyl components afforded a mixture of inseparable diastereomeric 3-*spiro*-1,2,4-trioxolanes. To address the challenge of stereochemical analysis of the obtained diastereomers, a chemometric approach based on partial least squares (PLS2) regression was applied. This method establishes a mathematical relationship between two data blocks—DFT-calculated and experimental ^13^C NMR signal sets, previously assigned by 2D NMR—and enables objective assignment of the stereochemical configuration to each experimentally observed diastereomer. Unlike manual matching and atom-by-atom ranking of chemical shifts, PLS2 simultaneously takes into account information from all carbon atoms, making the assignment statistically robust and reproducible. Moreover, in contrast to methods designed for individual compounds (DP4+) or unsupervised matching of datasets (MAEΔΔδ), PLS2 does not search for a correspondence but rather statistically verifies an already established (based on 2D NMR) signal assignment, linking each experimental set to a specific stereochemical configuration. This conceptually different approach is expected to offer a more objective and statistically rigorous framework for stereochemical assignment directly from mixture data.

Despite the synthetic success, the complete chromatographic separation of the four diastereomeric 3-spiro-1,2,4-trioxolanes proved unattainable. Therefore, the aim of the present study was to develop a reliable strategy for the stereochemical assignment of each diastereomer directly in the inseparable mixture. It was hypothesized that a chemometric PLS2 approach, linking experimental and DFT-calculated ^13^C NMR chemical shifts, could provide an objective and statistically robust assignment of the stereochemical configuration. To validate this approach, we combined single-crystal X-ray diffraction, high-resolution 2D NMR spectroscopy, and DFT calculations within the PLS2 framework.

## 2. Results and Discussion

**Scheme 1:**
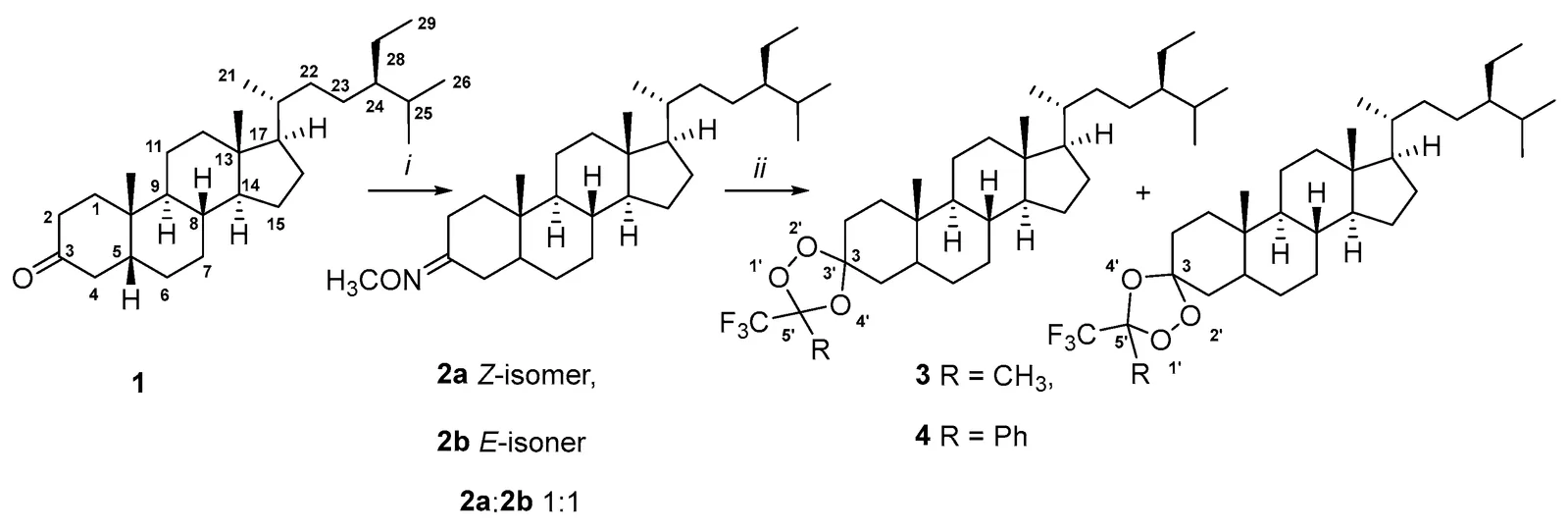
Reagents and conditions: *i*: MeONH_2_ · HCl, C_5_H_5_N–MeOH, reflux; *ii*: CF_3_(CO)CH_3_ (for **3**) or CF_3_(CO)C_6_H_5_ (for **4**), O_3_, −20 °C, CH_2_Cl_2_–cyclo-C_6_H_12_.

Sitostanone **1**, obtained by reduction of the C5(6)-double bond and following Jones oxidation of the C3-hydroxyl group of sitostanol according to [46], was used as the starting material (Scheme 1). The *O*-methyl oxime **2** was prepared by reaction of ketone **1** with methoxy-hydroxylamine in a pyridine–methanol mixture, yielding a Z/E mixture (**2a**:**2b**, 1:1) in 79% yield. Griesbaum co-ozonolysis [47] of **2** with CF_3_C(O)CH_3_ or CF_3_C(O)Ph afforded the corresponding 1,2,4-trioxolanes **3** and **4** as inseparable mixtures of four diastereomers in 67% and 59% yields, respectively.

The formation of four diastereomers can be rationalized by the mechanism of the Griesbaum co-ozonolysis. The reaction proceeds via a [3+2] cycloaddition between the carbonyl oxide, generated in situ from the *O*-methyl oxime **2**, and the fluorinated ketone. As demonstrated for substituted cyclohexanones [38], the stereochemical outcome is governed by the preferential axial attack of the carbonyl oxide on the carbonyl group.

**Figure 2:**
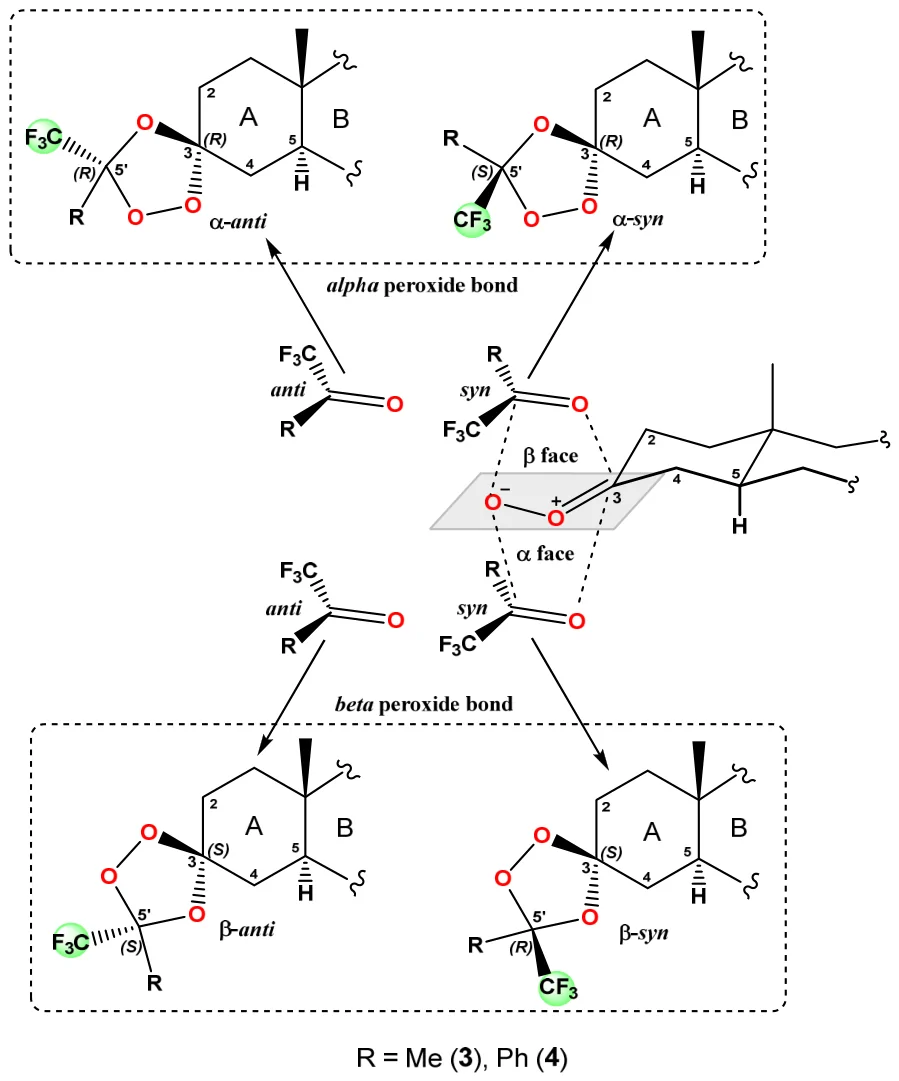
Stereochemical outcomes for the formation of the 1,2,4-trioxolane ring in **3** and **4**.

In the case of steroidal substrates such as sitostanone **1**, the conformational rigidity of the steroid A-ring further directs the attack from the α or β face, while the orientation of the trifluoromethyl group (*syn* or *anti*) is governed by the relative position of the ketone substituents during the cycloaddition. The combination of these steric and conformational factors accounts for the formation of the four diastereomers in non-statistical ratios, as observed experimentally. The diastereomers differ in two stereochemical features: (i) the α/β-orientation of the peroxide bridge with respect to steroid ring A, and (ii) the *syn*/*anti* orientation of the trifluoromethyl group at the stereogenic center C5′ relative to the C4 methylene group of ring A. Accordingly, the four diastereomers are designated as α-*anti* (3*R*,5′*R*), α-*syn* (3*R*,5′*S*), β-*anti* (3*S*,5′*S*), and β-*syn* (3*S*,5′*R*) (Figure 2).

**Figure 3:**
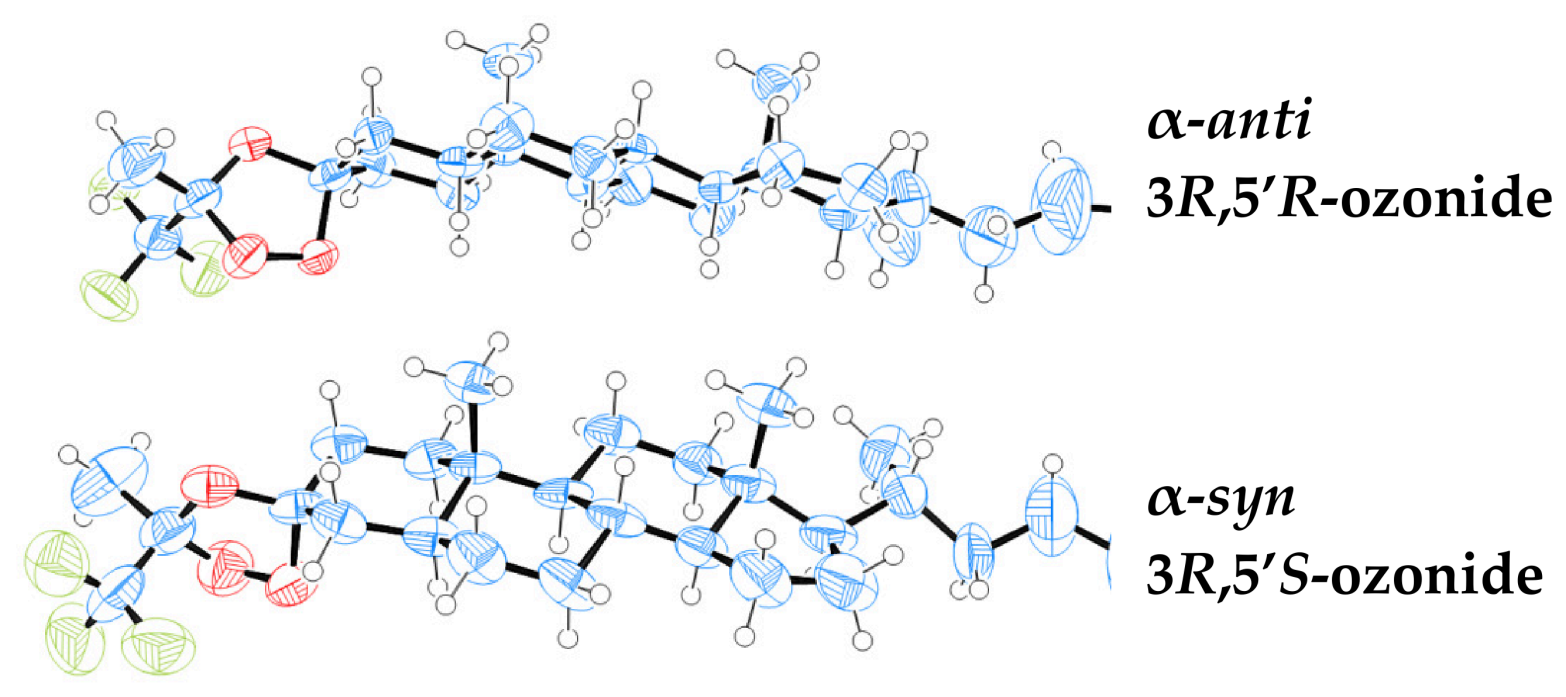
An ORTEP view of the α-isomers mixture of **3** showing 50% probability displacement ellipsoids. Oxygen atoms are highlighted in red, carbon atoms in blue, and fluorine atoms in green.

From the diastereomeric mixture of compound **3**, one pair of diastereomers was successfully isolated, and recrystallization from a petroleum ether–benzene mixture (1:1) afforded single crystals suitable for X-ray diffraction analysis. The crystal structure revealed two crystallographically independent molecules with α-*anti* (3*R*,5′*R*) and α-*syn* (3*R*,5′*S*) configurations of the 1,2,4-trioxolane ring (Figure 3). In both molecules, the bond lengths and angles are very similar and agree well with the statistical averages reported for related spiro compounds [48]. All cyclohexane fragments of the steroid nucleus adopt chair conformations, while the five-membered 1,2,4-trioxolane rings adopt twist conformations, with the O1′ and O2′ atoms deviating from the mean plane by approximately 0.4–0.5 Å. The C13–C14 bond shows a significant torsion, indicating strain in the five-membered ring D. Notably, in one of the independent molecules, the terminal ethyl group is disordered over two positions with an occupancy ratio of 0.80:0.20. Despite this disorder and the moderate quality of the crystal (R-factor = 9.56%), the X-ray data unambiguously established the absolute configuration of the α-anti and α-syn isomers. In the crystal structure of compound **3**, the molecules form a regular three-dimensional packing within the unit cell (Figure S1). The molecules are arranged periodically along the a and b axes with alternating orientations of the molecular fragments. The packing is characterized by efficient space filling without the formation of pronounced layered or chain motifs. Analysis of intermolecular distances reveals no abnormally short contacts between neighboring molecules, indicating the absence of specific directional interactions that would dominate the crystal packing. Importantly, no significant intermolecular interactions beyond standard van der Waals contacts, such as π-stacking or hydrogen bonding, were observed in the crystal packing, which accounts for the co-crystallization of two different diastereomers without the formation of solvates or dense packing motifs.

**Figure 4:**
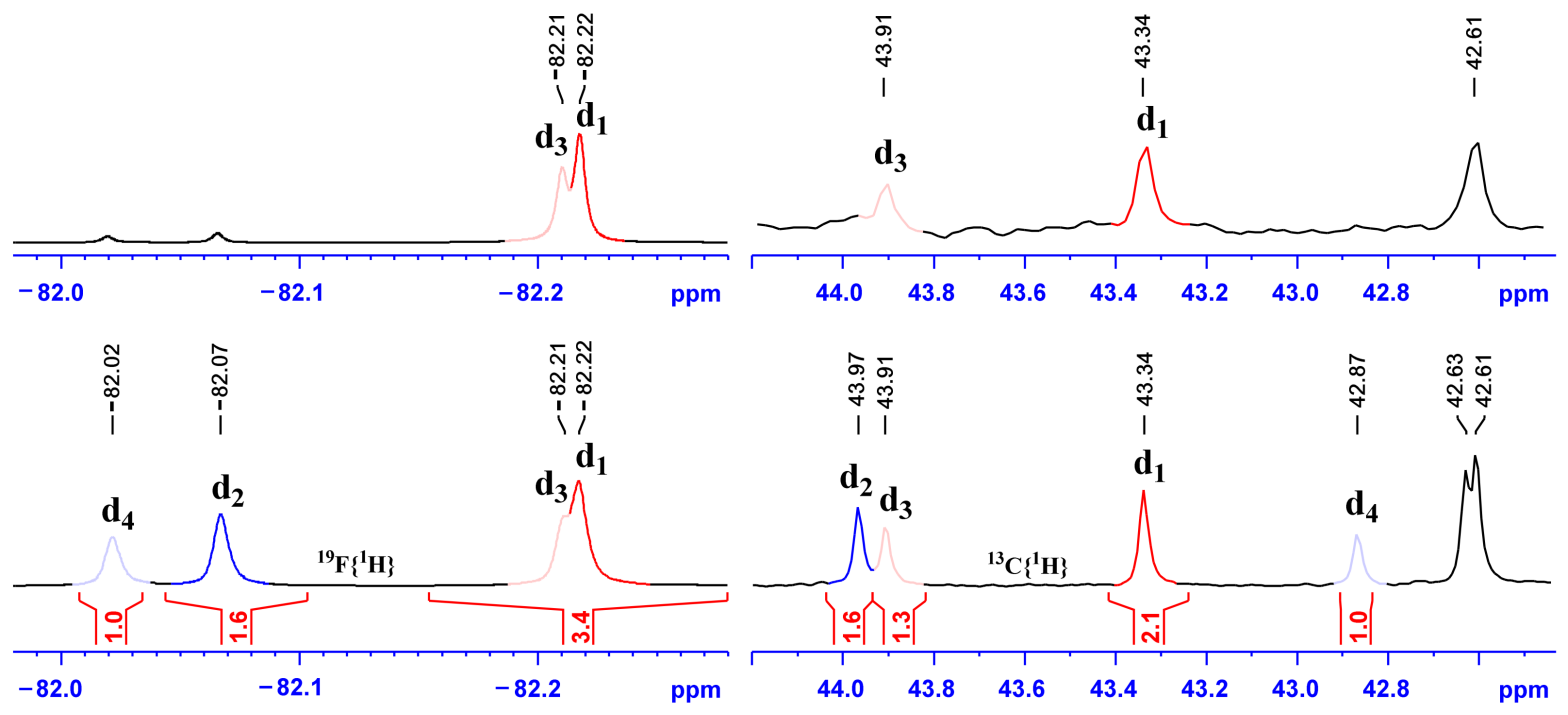
Discrimination of the C5 signals in the ^13^C{^1^H} NMR spectrum and the trifluoromethyl group in the ^19^F{^1^H} spectrum: initial mixture (**bottom**) vs. two-isomer mixture (**top**) for compound **3**.

**Figure 5:**
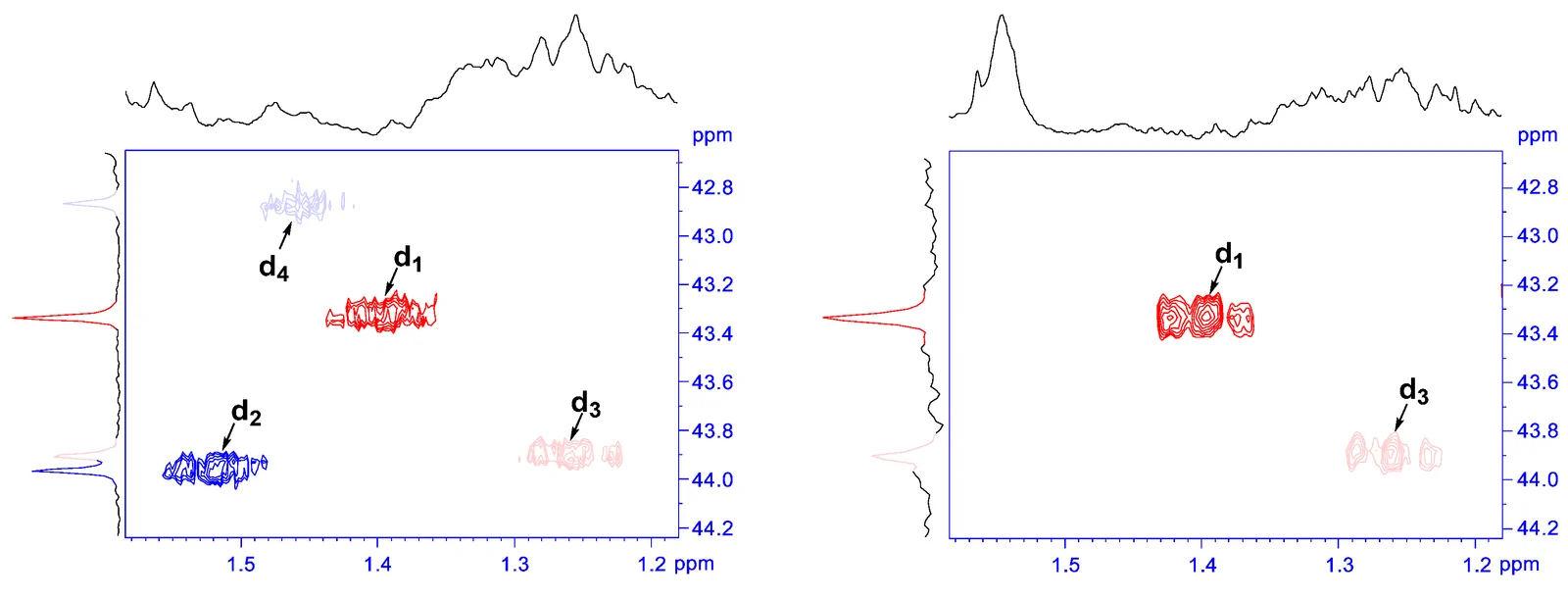
Correlation signals of the C5 methine groups in the {^1^H, ^13^C} HSQC spectrum of the initial mixture (**left**) and of the two-isomer mixture (**right**) for compound **3**.

For the diastereomeric mixture of compound **3** containing four diastereomers, as well as for the two-isomer α-fraction submitted for X-ray diffraction analysis, high-resolution 2D correlation experiments, including {^1^H, ^13^C} HSQC, {^1^H, ^13^C} HMBC, {^1^H, ^1^H} COSY-DQF, and {^19^F, ^1^H} HETCOR, enabled spectral separation and complete assignment of all ^1^H, ^13^C, and ^19^F resonances (see Section 3 and Table S1). Comparison of the spectra of the initial four-component mixture and the isolated α-pair (Figure 4 and Figure 5) allowed the signals belonging to the α-isomers (present in both fractions) to be distinguished from those of the β-isomers (absent in the X-ray fraction), providing preliminary assignments for the ^1^H, ^13^C, and ^19^F spectra. An additional criterion for reliable assignment of the three signals to the four diastereomers was the observed ^13^C signal intensity ratio of 2.0:1.6:1.3:1.0 for the diastereomers in the initial mixture, which was consistently reproduced for all non-overlapping signals. In the ^19^F{^1^H} NMR spectrum, the trifluoromethyl group signals were also resolved into four components with integral ratios fully matching those observed in the ^13^C{^1^H} spectra (Figure 4). For convenience in subsequent comparison with computational data, PLS2 model construction, and final stereochemical assignment, the diastereomers were labeled d_1_–d_4_ in order of decreasing abundance.

For each of the proposed diastereomers, the most favorable conformers with respect to pseudorotation within the 1,2,4-trioxolane ring and free rotation of the substituents were identified using molecular dynamics and Monte Carlo methods with the MMF94 force field as implemented in the PERCH modeling tool 2013.1 [49]. For the stationary points obtained for the four possible isomers, geometry optimizations, vibrational frequency calculations, and total energy evaluations were performed at the B3LYP/6-31G, B3LYP/6-31G(*d*), B3LYP/6-311+G(*2d*,*p*), and M062X/6-311+G(*d*,*p*) levels of theory, both in the gas phase and with implicit solvation (dichloromethane or chloroform) using the SMD model as implemented in Gaussian 16 [50] (see Table S2). DFT conformational analysis confirmed that the most stable conformers observed in the solid state by X-ray diffraction correspond to the dominant solution conformations used for the PLS2 models.

To evaluate the relative stability of the four diastereomers and to compare with the experimental ratios, thermodynamic parameters were calculated using the quasi-harmonic approximation (Table 1 and Table S3). This approach corrects the contribution of low-frequency vibrational modes (below 100 cm^−1^), which in the harmonic approximation often leads to an overestimated entropic contribution and, consequently, to non-physical populations [51,52]. For the entropic term, the Grimme quasi-harmonic (QS) approximation was employed, in which low-frequency modes are treated as free rotors, while for the enthalpic term, the Head-Gordon (QH) approximation was used, which includes a correction for the zero-point vibrational energy. All calculations were performed using the GoodVibes v3.2 program [53].

The best agreement with experiment was achieved at the M062X/6-311+G(*d*,*p*) + SMD (CHCl_3_) level (Bias = +0.008, R^2^ = 0.85). The high R^2^ values (0.85–0.89) indicate that thermodynamics qualitatively correctly predicts the relative stability order of the isomers. However, systematic deviations, namely overestimation of β-syn and underestimation of α-syn and β-anti populations, persist across all computational protocols, suggesting the presence of an additional factor not accounted for in the thermodynamic model (Table 1). Consequently, the product distribution in the Griesbaum co-ozonolysis is governed not by the thermodynamic stability of the final 1,2,4-trioxolanes, but rather by kinetic control at the cycloaddition stage of the carbonyl oxide to the ketone, which is consistent with literature data on the Griesbaum co-ozonolysis [37].

For the objective assignment of the stereochemical configuration to individual diastereomers, the partial least squares regression method was applied to the experimental ^13^C NMR signal sets. The PLS2 models (see Section 3) were constructed using ^13^C chemical shift data, since ^13^C resonances exhibit high sensitivity to stereochemical changes and cover a wide chemical shift range (0–220 ppm). Moreover, calculations of ^13^C magnetic shielding tensors using both the gauge-independent atomic orbital (GIAO) [54] and the continuous set of gauge transformations (CSGT) [55] methods are well-established and validated procedures.

To develop a systematic approach and evaluate the influence of the level of theory on the quality of chemical shift predictions, ^13^C magnetic shielding tensor (σ) calculations were performed at ten independent levels of theory, differing in functional, basis set, solvation model, and NMR calculation method (see Table S1). The calculations were carried out using the GIAO and CSGT methods at various levels, including B3LYP/6-31G, B3LYP/6-311G(*d*,*p*), and B3LYP/6-311+G(*2d*,*p*), as well as the well-established MPW1PW91/6-311+G(*2d*,*p*) approach [56] and the wB97XD/Def2SVP PCM (CHCl_3_) level [57] (see Tables S4 and S5).

**Figure 6:**
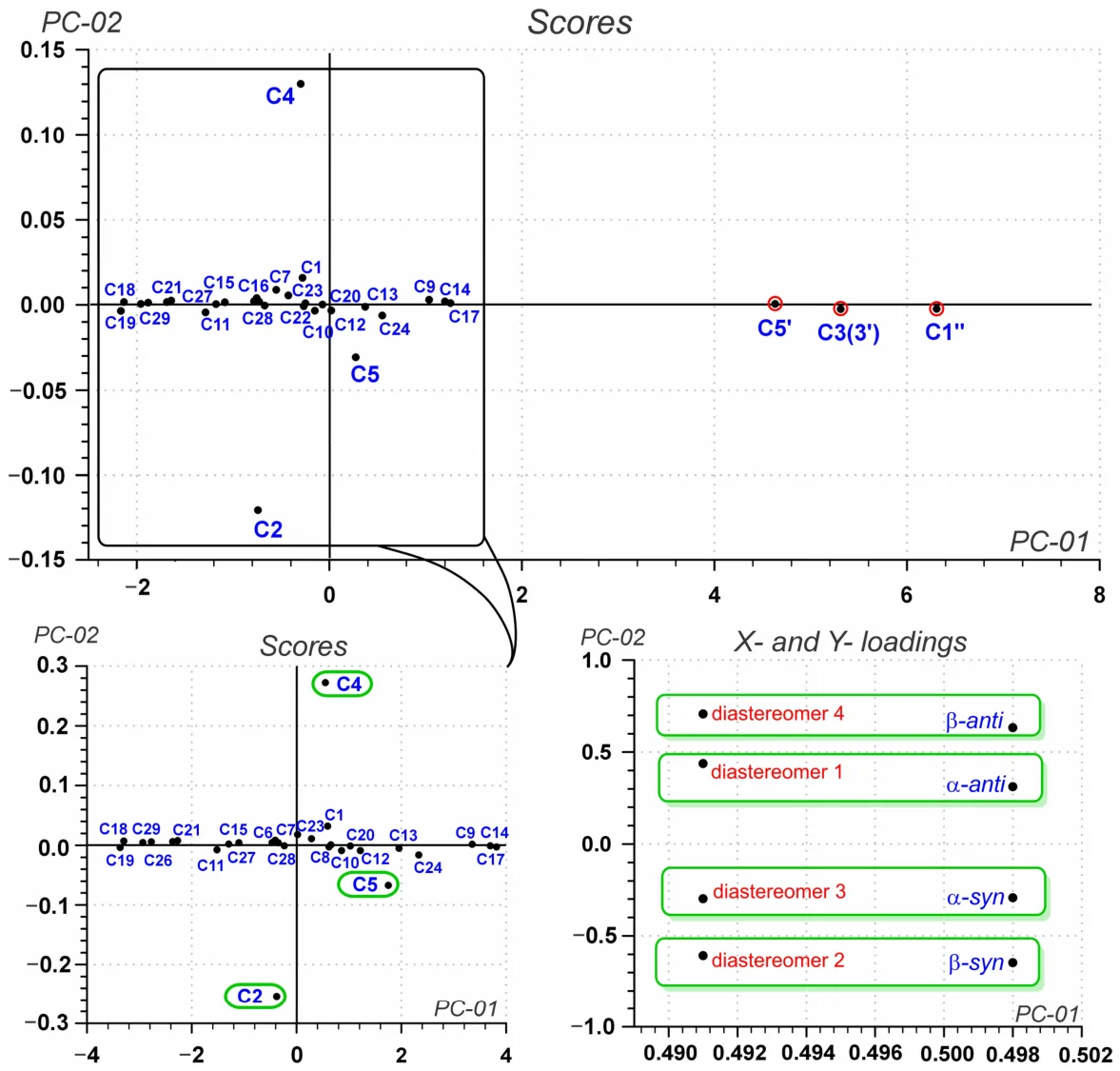
PLS2 analysis of **3** based on ^13^C NMR data calculated at the M062X/6-311+G(*d*,*p*) SMD//MPW1PW91/6-311+G(*2d*,*p*) CSGT level of theory before and after removal of structural outliers (atoms C3(3′), C5′, and C1″).

Analysis of the PLS2 model loadings of compound **3** revealed that atoms C3(3′), C5′, and C1″ exhibit anomalously high PC-01 (first principal component) values (>4.5), substantially exceeding those of the remaining carbon atoms. According to Tukey’s criterion (Q3 + 1.5 × IQR) [58], this identifies them as structural outliers (Figure 6, Figures S2 and S3). This behavior was observed consistently across all ten computational protocols, indicating a systematic nature of the deviation. The deviation arises, on the one hand, from the high sensitivity of the stereocenters C3′ and C5′—located in close proximity to the oxygen atoms of the 1,2,4-trioxolane ring to the chosen level of theory—and, on the other hand, from the fact that the C1″ atom is the trifluoromethyl carbon, for which standard basis sets typically afford systematic errors. Removal of these atoms from the predictor matrix X led to a substantial improvement in the model quality: the variance explained by the informative components PC-01 and PC-02 (second principal component) increased by more than a factor of four, from 1.02% to 4.14% and from 0.99% to 4.03%, respectively.

The refined PLS2 model of compound **3**, obtained after removal of the outliers C3(3′), C5′, and C1″, successfully resolves the four diastereomers along the second principal component (PC-02). PC-02 accounts for 3.9–4.1% of the total variance and serves a dual function: its sign defines the *syn*/*anti* orientation of the trifluoromethyl group (negative for anti, positive for syn), while its absolute value correlates with the α/β orientation of the peroxide bridge (systematically larger for β-isomers than for α-isomers) (see Table S6). The X-loadings clearly separate the calculated isomers into two groups, with α-anti and β-syn displaying positive PC-02 values (+0.300 and +0.639) and β-anti and α-syn displaying negative values (–0.647 and –0.291) (see Table S7). The Y-loadings exhibit an identical pattern for the experimental signals, establishing an unambiguous correspondence: d_1_ → α-anti, d_2_ → β-syn, d_3_ → α-syn, and d_4_ → β-anti, which holds for all computational protocols examined (see Tables S7 and S8).

The highest discrimination power along PC-02 is observed for the methylene carbons C2 and C4 of ring A. Their absolute loading values consistently exceed 0.25 across all computational protocols. This high sensitivity arises from their proximity to the stereogenic spiro center (atom C3(3′), which links the 1,2,4-trioxolane ring to ring A. Variations in the α/β orientation of the peroxide bridge affect the electron density in its bonds, while changes in the *syn*/*anti* orientation of the CF_3_ group modulate the spatial arrangement of the heterocycle relative to ring A. Both effects are directly reflected in the magnetic environment of C2 and C4. The methine carbon C5 shows markedly lower sensitivity, since its response to the *syn*/*anti* orientation is diminished by its distance from the trifluoromethyl group. Nevertheless, its signals still allow differentiation between α- and β-isomers (difference of ~0.5–1.0 ppm), making it an auxiliary diagnostic marker. Importantly, all of the observed trends, including the sign of PC-02 for *syn*/*anti* discrimination and the hierarchy of discrimination power (C4 > C2 >> C5), are reproduced across all computational protocols, regardless of the functional, basis set, solvation model, or NMR calculation method employed.

Comparative statistical analysis of the PLS2 models for compound **3** (see Table S6) allowed the identification of optimal computational protocols for practical application. For routine calculations and tasks where a balance between accuracy and computational cost is essential, the B3LYP/6-31G(d)//B3LYP/6-311+G(2d,p) GIAO level is recommended. This protocol provides high prediction quality (R^2^ = 0.954, RMSEC = 2.812) at minimal cost, making it suitable for widespread use in analogous studies. For tasks requiring maximum accuracy, the B3LYP/6-31G(d)//wB97XD/Def2SVP GIAO with PCM (CHCl3) approach is recommended. This protocol shows the best agreement with experiment among all the levels examined (R^2^ = 0.970, RMSEC = 2.266, Bias = +0.241, Slope = 0.852) at moderate computational expense. As an alternative for high accuracy, the B3LYP/6-311+G(2d,p)//MPW1PW91/6-311+G(2d,p) CSGT protocol may be used; it also gives excellent results (R^2^ = 0.959, RMSEC = 2.661, Slope = 0.858) but entails higher computational costs. Inclusion of solvation at the B3LYP/6-311+G(2d,p) SMD//MPW1PW91/6-311+G(2d,p) CSGT level improves the correlation (R^2^ = 0.966, Bias = +0.022) but introduces systematic bias (Slope = 0.785, Intercept = 5.390); therefore, it can only be recommended for specific solvent calculations, provided that systematic errors are carefully evaluated.

**Figure 7:**
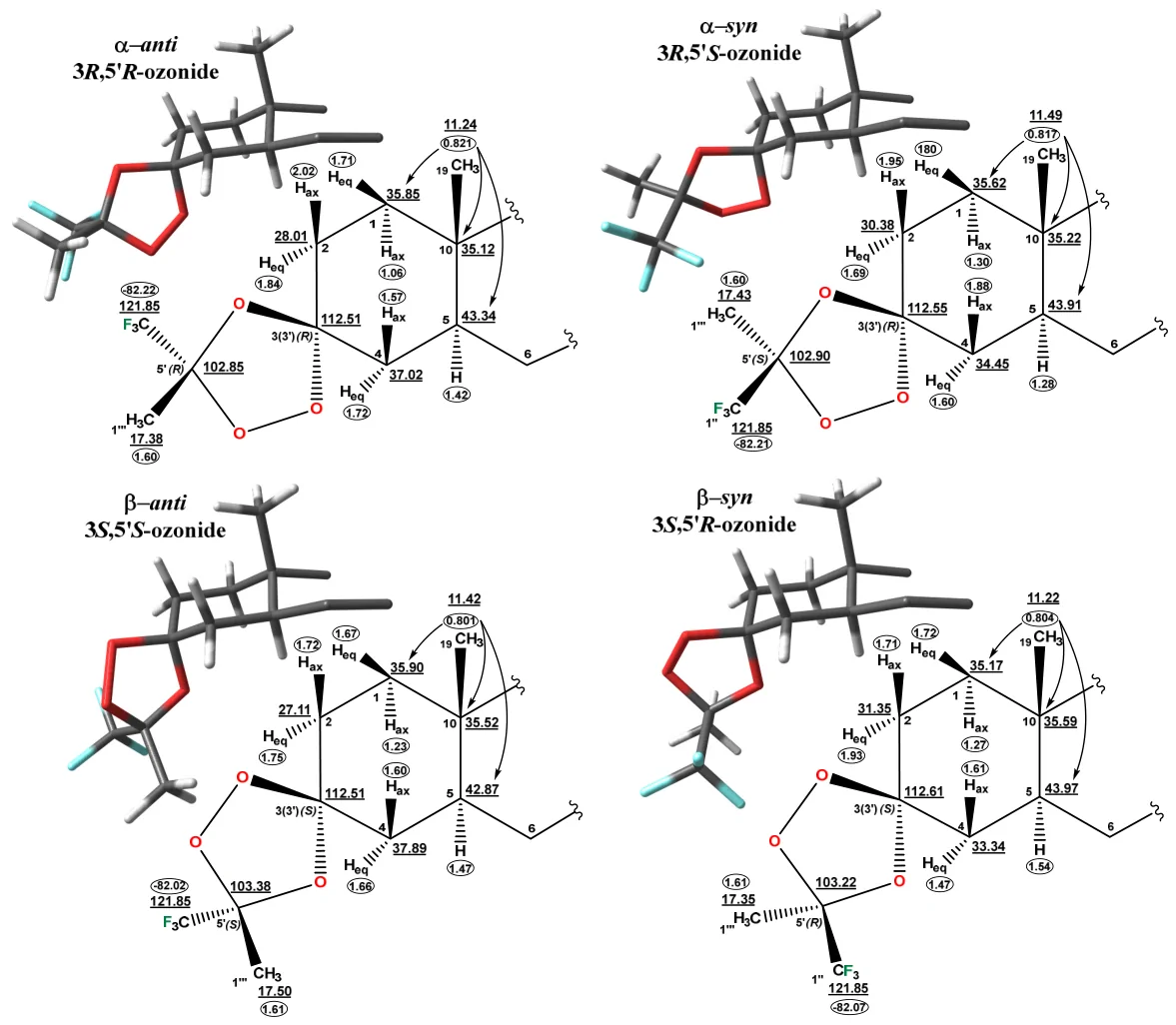
Structure and assignment of ^1^H and ^13^C NMR signals for diasteromeric spiro-1,2,4-trioxolanes **3**.

The combined use of X-ray diffraction, 2D NMR spectroscopy, and chemometric PLS2 analysis enabled the complete stereochemical assignment of all four diastereomers in the original mixture of **3**. The single-crystal X-ray data for the isolated α-pair of isomers served as the starting point for unambiguous calibration: the experimentally confirmed α-*anti* (3*R*,5′*R*) and α-*syn* (3*R*,5′*S*) structures were used to validate the calculated chemical shifts and to establish diagnostic spectral patterns for stereochemical differentiation. Two-dimensional correlation experiments (HSQC, HMBC, COSY-DQF, and HETCOR) provided complete assignment of the ^1^H, ^13^C, and ^19^F resonances in both the original mixture and the isolated α-fraction (Figure 7). The final step was the PLS2 analysis, which, through statistical comparison of the calculated and experimental ^13^C chemical shifts for all four possible diastereomers, allowed objective and reproducible assignment of each experimental diastereomer in the original mixture to a specific stereochemical configuration.

The PLS2 methodology, validated on compound **3**, was then applied to the structurally related compound **4**. From the original mixture of four diastereomers of **4** (ratio 2.3:2.2:1.3:1, labeled d_1_–d_4_), a fraction enriched in isomers d_1_ and d_2_ was isolated. High-resolution 2D NMR spectra, recorded for both the original four-component mixture and the enriched d_1_–d_2_ fraction, enabled comparative analysis and reliable separation of overlapping signals. As a result, complete assignment of the ^1^H, ^13^C, and ^19^F resonances was achieved for all four experimentally observed diastereomers (see Section 3 and Table S1).

In the next step, PLS2 analysis of the calculated and experimental ^13^C chemical shifts was performed to verify the assignments and to objectively correlate the signals of diastereomers d_1_–d_4_ with their stereochemical configurations. Identification of structural outliers in the PLS2 models of **4** revealed that statistically significant deviations (PC-01 > 3.675 according to Tukey’s criterion) were observed for the phenyl ring atoms and the trifluoromethyl carbon C1″, while C3(3′) and C5′ did not exceed this threshold (see Figures S4 and S5 and Table S9). PLS2 analysis of the thus-cleaned ^13^C data for the diastereomeric mixture of **4** allowed objective and reproducible assignment of a specific stereochemical configuration to each of the experimentally observed diastereomers: d_1_ → β-*syn*, d_2_ → β-*anti*, d_3_ → α-*syn*, and d_4_ → α-*anti* (see Tables S8 and S10).

Comparison of the statistical parameters of the PLS2 models for **3** (see Table S6) and **4** (see Table S11), after removal of structural outliers, showed that both the proportions of variance explained by PC-02 (3.9–4.2% and 3.6–4.5%, respectively) and the coefficients of determination R^2^ (0.939–0.970 and 0.950–0.970, respectively) are comparable. This confirms the high predictive power and transferability of the proposed chemometric approach, as well as the recommended computational protocols. The best results for both compounds were achieved with the B3LYP/6-31G(*d*)//wB97XD/Def2SVP GIAO with PCM level, for which R^2^ reaches 0.970 and RMSEC ranges from 2.266 to 2.272.

It should be emphasized that the stereochemical assignments obtained are consistent across all computational protocols examined, for both the raw and the refined PLS2 models. The removal of structural outliers was necessary to improve the statistical significance of the models, which supports the reliability of the assignments and their independence from the chosen level of theory and the outlier removal procedure. Furthermore, the atoms C2, C4, and C5 of ring A retain their discrimination power for **4**, confirming their general utility as diagnostic markers for 3-*spiro*-1,2,4-trioxolane derivatives of *β*-sitostanol.

The combination of X-ray diffraction analysis, 2D NMR spectroscopy, and PLS2 analysis proved to be an effective strategy for the reliable stereochemical identification of complex diastereomeric mixtures of steroidal and other types of 1,2,4-trioxolanes.

## 3. Materials and Methods

### 3.1. General Experimental Procedures

The NMR spectra of the synthesized compounds were recorded in CDCl_3_ solution on a Bruker Avance-III 500 MHz spectrometer (Billerica, MA, USA) equipped with a PABBO X direct-detection probe using 5 mm NMR tubes at 298 °K. The spectrometer operated at 500.13 MHz for ^1^H, 470.59 MHz for ^19^F, 125.76 MHz for ^13^C, and 50.68 MHz for ^15^N. ^1^H and ^13^C chemical shifts are reported in parts per million (ppm) relative to tetramethylsilane (TMS) as the internal standard. ^19^F chemical shifts are referenced to CFCl_3_ as the external standard. ^15^N chemical shifts are referenced to external liquid ammonia at 25 °C. ^1^H NMR spectra were acquired with a spectral width of 10 kHz, 64k data points, and 8 scans, providing a digital resolution of ca. 0.3 Hz (90° ^1^H pulse width = 11.5 μs). ^19^F NMR spectra were recorded with a spectral width of 7 kHz, 16k data points, 32 scans, a relaxation delay of 12.5 s, and a 90° ^19^F pulse width of 15.0 μs. ^19^F NMR spectra were acquired using zgflqn (for ^19^F) and zgfhigqn.2 (for ^19^F{^1^H}) sequences with a spectral width of 7.06 kHz, 16k data points, 64 scans, a 90° ^19^F pulse width = 11.5 μs, and a digital resolution of 0.86 Hz. ^13^C{^1^H} power-gated NMR spectra were acquired with a spectral width of 29.7 kHz, 64k data points, and the required number of scans (90° ^13^C pulse width = 9.7 μs). Gradient-selected {^1^H, ^13^C} HSQC spectra were recorded using the standard Bruker pulse sequences (hsqcetgp and hsqcedetgp). Data were collected with 2048 × 1024 data points, spectral widths of 6.8 kHz (*F*1, ^13^C) and 1.0 kHz (*F*2, ^1^H), and 2 scans per increment. The delay d4 was set to 1.72 ms. Gradient-selected {^1^H, ^13^C} HMBC spectra (hmbcgpndqf) were acquired with 4096 × 2048 data points, spectral widths of 17.6 kHz (*F*1, ^13^C) and 1.0 kHz (*F*2, ^1^H), and 2 or 6 scans per increment. The delay d6 was set to 71.4 ms. {^1^H, ^13^C} HSQC and HMBC data were processed using a sine-bell window function in both dimensions. {^1^H, ^15^N} gs-HMBC spectra (hmbcgpndqf) were recorded with 4096 × 512 data points, 12 scans per increment, and delays d6 = 142.8 ms. Spectral widths of 2.0 kHz (*F*2, ^1^H) and 20.2 kHz (*F*1, ^15^N) were used. {^19^F, ^1^H} HETCOR spectra were recorded using the standard Bruker sequence (hfcoqfqn). Data were collected with 8192 × 512 data points, spectral widths of 7.0 kHz (*F*2, ^19^F) and 1.0 kHz (*F*1, ^1^H), and 4 scans per increment. The delay d2 was set to 41.7 ms. {^1^H, ^1^H} COSY-DQF spectra were collected with 4096 × 1024 data points and 2 scans per increment. For {^1^H, ^1^H} NOESY experiments, the sample was degassed to remove dissolved oxygen; spectra were acquired with a spectral width of 2.0 kHz, 4096 data points, 512 increments, 2 transients per increment, and a mixing time of 0.5 s. Fourier transformation was performed with zero-filling and a shifted sine-bell apodization function in both dimensions for all 2D experiments. High-resolution mass spectrometry (HRMS) was performed using an Agilent LC/Q-TOF 6530 instrument (Santa Clara, CA, USA). Thin-layer chromatography analyses were performed on Sorbfil plates (Sorbpolimer, Krasnodar, Russia) using the solvent system chloroform–ethyl acetate, 40:1. Substances were detected by a 10% sulfuric acid solution with subsequent heating at 100–120 °C for 2–3 min. All chemicals were of reagent grade (Sigma-Aldrich, St. Louis, MO, USA). All spectral characteristics for the compounds **2**–**4** are included in the Supplementary Materials (see Figures S6–S56). Compound **1** was obtained according to the method described previously [46].

### 3.2. X-Ray Diffraction Analysis

XRD data for α-isomers mixture of **3d_1_**, **3d_4_** were obtained on a Bruker Kappa Apex II CCD diffractometer (Billerica, MA, USA) using φ, ω scans of narrow (0.8°) frames with Mo Kα radiation (λ = 0.71073 Å) and a graphite monochromator at T = 200(2) K. The structure was solved by direct methods and refined by the full-matrix least-squares method against all F2 in an anisotropic approximation using the SHELXT–2014/5 [59] and SHELXL-2018/3 [60] software suites. The positions of hydrogen atoms were calculated using the riding model. The SQUEEZE procedure (PLATON program version 2026.1 [61]) was used due to a highly disordered solvent molecule. The obtained crystal structures were analyzed for short contacts between nonbonded atoms in the PLATON and MERCURY software packages version 4.2.0 [62]. Absorption corrections were applied by the empirical multiscan method in SADABS Version 2008-1 software [63].

### 3.3. Chemometric Analysis (PLS2)

To objectively assign the experimental signals to the four diastereomers in the inseparable mixture, the method of partial least squares (PLS2) regression was applied. PLS2 is a multivariate statistical method that establishes a mathematical relationship between two data blocks: the X matrix (predictors) and the Y matrix (responses). The X matrix (predictors) consisted of the calculated ^13^C NMR chemical shifts for the four stereoisomers (α-anti, β-syn, α-syn, and β-anti), with each column corresponding to one isomer and each row to a carbon atom. The Y matrix (responses) contained the experimental chemical shifts for the four signals in the mixture (d_1_, d_2_, d_3_, d_4_). PLS2 regression was performed using the *pls2_nipals* function from the *chemometrics* package version 1.4.4 [64] within the *R* statistical computing environment version 4.5.3 [65]. The method is based on the NIPALS algorithm, as described in the fundamental chemometrics textbook by Varmuza and Filzmoser [66]. All data were mean-centered and scaled. Model validation was conducted using full cross-validation with the appropriate functions from the chemometrics package. The PLS2 model is described by two principal components: PC-01 (the first principal component) reflects the overall scale of chemical shifts and correlates with the type of carbon atom hybridization and its electronic environment; PC-02 (the second principal component) separates the four isomers into two groups based on their stereochemical configuration. For each carbon atom, coordinates in the PC-01/PC-02 space (Scores) were obtained, allowing the identification of atoms with the highest discriminatory power. Analysis of the loadings for the X and Y matrices enabled an unambiguous correspondence between the experimental signals and the structural isomers to be established.

### 3.4. The Procedure for Synthesis of Compound ***2***

*O*-Methyl-3-oximino-24-ethyl-5α-cholestan (**2**). CH_3_ONH_2_·HCl (0.17 g, 2 mmol) was added to the solution of compound **1** (0.41 g, 1 mmol) in a mixture of pyridine and methanol (30 mL, 1:1, *v*/*v*). The reaction mixture was refluxed for 8 h with a back condenser, cooled to room temperature, and quenched with 5% HCl (150 mL). The precipitate was filtered off, washed with water, and air-dried. A residue was purified by column chromatography (petroleum ester–ethyl acetate 60:1 → 1:1, chloroform as the eluents). The yield of amorphous substance was 0.36 g (89%) as a mixture of Z/E-isomers. Anal. Calcd. for C_30_H_53_NO: C, 81.20; H, 12.04; N, 3.16. Found: C, 80.95; H, 12.01; N, 3.12.

#### 3.4.1. *O*-Methyl-3(*Z*)-oximino-24-ethyl-5α-cholestan (**2a**)

^13^C NMR (CDCl_3_, δ ppm): 11.50 (C19); 11.99 (C29); 12.08 (C18); 18.74 (C21); 19.05 (C26); 19.82 (C27); 21.18 (C11); 23.07 (C28); 24.22 (C15); 26.07 (C23); 27.83 (C2); 27.91 (C4); 28.26 (C16); 28.87 (C6); 29.15 (C25); 31.82 (C7); 33.92 (C22); 35.42 (C8); 36.15 (C10); 36.16 (C20); 38.47 (C1); 39.97 (C12); 42.58 (C13); 45.44 (C5); 45.84 (C24); 54.04 (C9); 56.16 (C17); 56.39 (C14); 60.99 (C31); 160.08 (C3). ^15^N NMR (CDCl_3_, δ ppm): 355.47 (N30). ^1^H NMR (CDCl_3_, δ ppm, *J* Hz): 0.66 (s, 3H, H-18); 0.68 (m, 1H, H-9); 0.81 (d, 3H, ^3^*J*_26-25_ = 6.8, H-26); 0.83 (d, 3H, ^3^*J*_27-25_ = 6.8, H-27); 0.84 (t, 3H, ^3^*J*_29-28_ = 7.3, H-29); 0.87 (m, 1H, H_ax_-7); 0.88 (s, 3H, H-19); 0.91 (d, 3H, ^3^*J*_21-20_ = 6.5, H-21); 0.93 (m, 1H, H-24); 0.99 (m, 1H, H_A_-22); 1.00 (m, 1H, H-14); 1.04 (m, 1H, H_β_-15); 1.10 (m, 1H, H-17); 1.12 (ddd, 1H, ^2^*J* = 13.3, ^3^*J*_1ax-2ax_ = 14.8, ^3^*J*_1ax-2eq_ = 4.8, H_ax_-1); 1.12 (m, 1H, H_ax_-12); 1.16 (m, 2H, H-23); 1.22 (m, 1H, H-5); 1.24 (m, 1H, H_A_-28); 1.24 (m, 1H, H_β_-16); 1.25 (m, 1H, H_ax_-6); 1.28 (m, 1H, H_B_-28); 1.31 (m, 1H, H_ax_-11); 1.33 (m, 1H, H_B_-22); 1.35 (m, 1H, H-8); 1.35 (m, 1H, H-20); 1.36 (m, 1H, H_eq_-6); 1.49 (m, 1H, H_eq_-11); 1.56 (m, 1H, H_α_-15); 1.65 (m, 1H, H_ax_-4); 1.67 (m, 1H, H-25); 1.67 (m, 1H, H_eq_-7); 1.83 (m, 1H, H_α_-16); 1.87 (ddd, 1H, ^2^*J* = 13.3, ^3^*J*_1eq-2ax_ = 5.3, ^3^*J*_1eq-2eq_ = 2.4, H_eq_-1); 1.97 (dt, 1H, ^2^*J* = 12.6, ^3^*J*_12eq-11ax_ = 3.2, ^3^*J*_12eq-11eq_ = 3.2, H_eq_-12); 2.20 (td, 1H, ^2^*J* = 14.8, ^3^*J*_2ax-1ax_ = 14.8, ^3^*J*_2ax-1eq_ = 5.3, H_ax_-2); 2.27 (dddd, 1H, ^2^*J* = 14.8, ^3^*J*_2eq-1ax_ = 4.8, ^3^*J*_2eq-1eq_ = 2.4, ^4^*J*_2eq-4eq_ = 1.3, H_eq_-2); 2.89 (ddd, 1H, ^2^*J* = 15.1, ^3^*J*_4eq_-_5_ = 3.5, ^4^*J*_4eq-2eq_ = 1.3, H_eq_-4); 3.81 (s, 3H, H-31).

#### 3.4.2. *O*-Methyl-3(*E*)-oximino-24-ethyl-5α-cholestan (**2b**)

^13^C NMR (CDCl_3_, δ ppm): 11.33 (C19); 11.99 (C29); 12.07 (C18); 18.74 (C21); 19.05 (C26); 19.82 (C27); 21.14 (C2); 21.22 (C11); 23.07 (C28); 24.22 (C15); 26.07 (C23); 28.26 (C16); 28.69 (C6); 29.15 (C25); 31.80 (C7); 33.92 (C22); 34.45 (C4); 35.42 (C8); 36.11 (C10); 36.16 (C20); 37.41 (C1); 39.97 (C12); 42.56 (C13); 45.84 (C24); 46.68 (C5); 53.96 (C9); 56.16 (C17); 56.40 (C14); 60.98 (C31); 160.12 (C3). ^15^N NMR (CDCl_3_, δ ppm): 357.06 (N30). ^1^H NMR (CDCl_3_, δ ppm, *J* Hz): 0.66 (s, 3H, H-18); 0.67 (m, 1H, H-9); 0.81 (d, 3H, ^3^*J*_26-25_ = 6.8, H-26); 0.83 (d, 3H, ^3^*J*_27-25_ = 6.8, H-27); 0.84 (t, 3H, ^3^*J*_29-28_ = 7.3, H-29); 0.87 (m, 1H, H_ax_-7); 0.88 (s, 3H, H-19); 0.91 (d, 3H, ^3^*J*_21-20_ = 6.5, H-21); 0.93 (m, 1H, H-24); 0.99 (m, 1H, H_A_-22); 1.00 (m, 1H, H-14); 1.04 (ddd, 1H, ^2^*J* = 13.3, ^3^*J*_1ax-2ax_ = 14.8, ^3^*J*_1ax-2eq_ = 4.8, H_ax_-1); 1.04 (m, 1H, H_β_-15); 1.10 (m, 1H, H-17); 1.12 (m, 1H, H_ax_-12); 1.16 (m, 2H, H-23); 1.24 (m, 1H, H_A_-28); 1.24 (m, 1H, H_β_-16); 1.26 (m, 1H, H_ax_-6); 1.28 (m, 1H, H-5); 1.28 (m, 1H, H_B_-28); 1.31 (m, 1H, H_ax_-11); 1.33 (m, 1H, H_B_-22); 1.35 (m, 1H, H-8); 1.35 (m, 1H, H-20); 1.35 (m, 1H, H_eq_-6); 1.49 (m, 1H, H_eq_-11); 1.56 (m, 1H, H_α_-15); 1.67 (m, 1H, H-25); 1.67 (m, 1H, H_eq_-7); 1.81 (td, 1H, ^2^*J* = 14.8, ^3^*J*_2ax-1ax_ = 14.8, ^3^*J*_2ax-1eq_ = 5.3, H_ax_-2); 1.82 (ddd, 1H, ^2^*J* = 13.3, ^3^*J*_1eq-2ax_ = 5.3, ^3^*J*_1eq-2eq_ = 2.4, H_eq_-1); 1.83 (m, 1H, H_α_-16); 1.97 (dt, 1H, ^2^*J* = 12.6, ^3^*J*_12eq-11ax_ = 3.2, ^3^*J*_12eq-11eq_ = 3.2, H_eq_-12); 2.02 (m, 1H, H_eq_-4); 2.04 (m, 1H, H_ax_-4); 3.12 (dddd, 1H, ^2^*J* = 14.8, ^3^*J*_2eq-1ax_ = 4.8, ^3^*J*_2eq-1eq_ = 2.4, ^4^*J*_2eq-4eq_ = 1.3, H_eq_-2); 3.80 (s, 3H, H-31).

### 3.5. General Procedure of Griesbaum Co-Ozonolysis

Ozone was bubbled through a solution of compound **2** (0.47 g, 1 mmol) in a mixture of cyclohexane and CH_2_Cl_2_ (30 mL, 1:2, *v*/*v*) in the presence of CF_3_C(O)CH_3_ (0.18 mL, 2 mmol) (for compound **3**) or CF_3_C(O)Ph (0.28 mL, 2 mmol) (for compound **4**) at −20 °C with TLC control. After completion of the reaction, the solution was flushed with oxygen for 5 min before being concentrated in vacuo at room temperature to give a residue that was then purified by column chromatography (hexane as eluent).

#### 3.5.1. 3(*R*,*S*),5′(*R*,*S*)-3(3′)-Spiro-[(5′-trifluoromethyl-5′-methyl)-1′,2′,4′-trioxolane]-24-ethyl-5α-cholestan **3**

Yield g 0.36 g (67%) as a mixture of four isomers. Anal. Calcd. for C_32_H_53_F_3_O_3_: C, 70.81 anti (3R,5′R) and α-syn (3R,5′S) configurations H, 9.84; F, 10.50. Found: C, 70.69; H, 9.75; F, 10.43.

#### 3.5.2. 3(*R*),5′(*R*)-3(3′)-Spiro-[(5′-trifluoromethyl-5′-methyl)-1′,2′,4′-trioxolane]-24-ethyl-5α-cholestan **3** α-Anti (d_1_)

^1^H NMR (CDCl_3_, δ ppm, *J* Hz): 0.65 (s, 3H, H-18); 0.75 (m, 1H, H-9); 0.82 (s, 3H, H-19); 0.82 (d, 3H, ^3^*J*_26-25_ = 6.8, H-26); 0.83 (d, 3H, ^3^*J*_27-25_ = 6.8, H-27);0.84 (t, 3H, ^3^*J*_29-28_ = 7.5, H-29); 0.90 (d, 3H, ^3^*J*_21-20_ = 6.5, H-21);0.93 (m, 1H, H_ax_-7); 0.94 (m, 1H, H-24); 1.01 (m, 1H, H-14); 1.03 (m, 1H, H_A_-22); 1.06 (m, 1H, H_ax_-1); 1.06 (m, 1H, H_β_-15); 1.13 (m, 1H, H-17); 1.15 (m, 1H, H_ax_-12); 1.17 (m, 2H, H-23); 1.22 (m, 1H, H_A_-28); 1.27 (m, 1H, H_β_-16); 1.28 (m, 1H, H_B_-28); 1.30 (m, 1H, H_ax_-11); 1.30 (m, 1H, H_ax_-6); 1.32 (m, 1H, H_eq_-6); 1.34 (m, 1H, H_B_-22); 1.35 (m, 1H, H-8); 1.37 (m, 1H, H-20); 1.42 (m, 1H, H-5); 1.49 (m, 1H, H_eq_-11); 1.57 (m, 1H, H_ax_-4); 1.58 (m, 1H, H_α_-15); 1.60 (s, 3H, H-1‴); 1.67 (m, 1H, H-25); 1.69 (m, 1H, H_eq_-7); 1.71 (m, 1H, H_eq_-1); 1.72 (m, 1H, H_eq_-4); 1.82 (m, 1H, H_α_-16); 1.84 (m, 1H, H_eq_-2); 1.98 (m, 1H, H_eq_-12); 2.02 (m, 1H, H_ax_-2). ^13^C NMR (CDCl_3_, δ ppm): 11.24 (C19); 12.00 (C29); 12.08 (C18); 17.38 (C1‴); 18.75 (C21); 19.07 (C26); 19.83 (C27); 21.16 (C11); 23.12 (C28); 24.23 (C15); 26.15 (C23); 28.01 (C2); 28.27 (C16); 28.48 (C6); 29.22 (C25); 31.78 (C7); 33.97 (C22); 35.12 (C10); 35.45 (C8); 35.85 (C1); 36.18 (C20); 37.02 (C4); 39.93 (C12); 42.61 (C13); 43.34 (C5); 45.89 (C24); 53.75 (C9); 56.18 (C17); 56.36 (C14); 102.85 (q, ^2^*J*_CF_ = 33.3, C5′); 112.51 (C3(3′);); 121.85 (q, ^1^*J*_CF_ = 289.1, C1″); ^19^F NMR (CDCl_3_, δ ppm): −82.22 (F-1″).

#### 3.5.3. 3(*S*),5′(*R*)-3(3′)-Spiro-[(5′-trifluoromethyl-5′-methyl)-1′,2′,4′-trioxolane]-24-ethyl-5α-cholestan **3** β-Syn (d_2_)

^1^H NMR (CDCl_3_, δ ppm, *J* Hz): 0.66 (s, 3H, H-18); 0.75 (m, 1H, H-9); 0.82 (s, 3H, H-19); 0.82 (d, 3H, ^3^*J*_26-25_ = 6.8, H-26); 0.83 (d, 3H, ^3^*J*_26-25_ = 6.8, H-27); 0.84 (t, 3H, ^3^*J*_29-28_ = 7.5, H-29); 0.91 (d, 3H, ^3^*J*_21-20_ = 6.5, H-21); 0.93 (m, 1H, H_ax_-7); 0.94 (m, 1H, H-24); 1.01 (m, 1H, H-14); 1.03 (m, 1H, H_A_-22); 1.06 (m, 1H, H_β_-15); 1.11 (m, 1H, H-17); 1.15 (m, 1H, H_ax_-12); 1.17 (m, 2H, H-23); 1.22 (m, 1H, H_A_-28); 1.27 (m, 1H, H_β_-16); 1.27 (m, 1H, H_ax_-1); 1.28 (m, 1H, H_B_-28); 1.30 (m, 1H, H_ax_-11); 1.30 (m, 1H, H_ax_-6); 1.32 (m, 1H, H_eq_-6); 1.34 (m, 1H, H_B_-22); 1.35 (m, 1H, H-8); 1.37 (m, 1H, H-20); 1.47 (m, 1H, H_eq_-4); 1.49 (m, 1H, H_eq_-11); 1.54 (m, 1H, H-5); 1.58 (m, 1H, H_α_-15); 1.61 (m, 1H, H-1‴); 1.61 (m, 1H, H_ax_-4); 1.67 (m, 1H, H-25); 1.69 (m, 1H, H_eq_-7); 1.71 (m, 1H, H_ax_-2); 1.72 (m, 1H, H_eq_-1); 1.82 (m, 1H, H_α_-16); 1.93 (m, 1H, H_eq_-2); 1.98 (m, 1H, H_eq_-12). ^13^C NMR (CDCl_3_, δ ppm): 11.22 (C19); 12.00 (C29); 12.09 (C18); 17.35 (C1‴); 18.75 (C21); 19.07 (C26); 19.83 (C27); 21.31 (C11); 23.12 (C28); 24.23 (C15); 26.17 (C23); 28.24 (C6); 28.27 (C16); 29.22 (C25); 31.35 (C2); 31.78 (C7); 33.34 (C4); 33.97 (C22); 35.17 (C1); 35.45 (C8); 35.59 (C10); 36.18 (C20); 39.95 (C12); 42.63 (C13); 43.97 (C5); 45.89 (C24); 53.75 (C9); 56.22 (C17); 56.44 (C14); 103.22 (q, ^2^*J*_CF_ = 33.3, C5′); 112.61 (C3(3′)); 121.85 (q, ^1^*J*_CF_ = 289.1, C1″). ^19^F NMR (CDCl_3_, δ ppm): −82.07 (F-1″).

#### 3.5.4. 3(*R*),5′(*S*)-3(3′)-Spiro-[(5′-trifluoromethyl-5′-methyl)-1′,2′,4′-trioxolane]-24-ethyl-5α-cholestan **3** α-Syn (d_3_)

^1^H NMR (CDCl_3_, δ ppm, *J* Hz): 0.66 (s, 3H, H-18); 0.67 (m, 1H, H-9); 0.80 (s, 3H, H-19); 0.82 (d, 3H, ^3^*J*_26-25_ = 6.8, H-26); 0.83 (d, 3H, ^3^*J*_27-25_ = 6.8, H-27); 0.84 (t, 3H, ^3^*J*_29-28_ = 7.5, H-29); 0.88 (m, 1H, H_ax_-7); 0.91 (d, 3H, ^3^*J*_21-20_ = 6.45 Hz, H-21); 0.94 (m, 1H, H-24); 1.01 (m, 1H, H-14); 1.03 (m, 1H, H_A_-22); 1.06 (m, 1H, H_β_-15); 1.11 (m, 1H, H-17); 1.15 (m, 1H, H_ax_-12); 1.17 (m, 2H, H-23); 1.22 (m, 1H, H_A_-28); 1.27 (m, 1H, H_β_-16); 1.28 (m, 1H, H_B_-28); 1.28 (m, 1H, H-5); 1.30 (m, 1H, H_ax_-11); 1.30 (m, 1H, H_ax_-6); 1.30 (m, 1H, H_ax_-1); 1.32 (m, 1H, H_eq_-6); 1.34 (m, 1H, H_B_-22); 1.35 (m, 1H, H-8); 1.37 (m, 1H, H-20); 1.49 (m, 1H, H_eq_-11); 1.58 (m, 1H, H_α_-15); 1.60 (s, 3H, H-1‴); 1.60 (m, 1H, H_eq_-4); 1.67 (m, 1H, H-25); 1.67 (m, 1H, H_eq_-7); 1.69 (m, 1H, H_eq_-2); 1.80 (m, 1H, H_eq_-1); 1.82 (m, 1H, H_α_-16); 1.88 (m, 1H, H_ax_-4); 1.95 (m, 1H, H_ax_-2); 1.98 (m, 1H, H_eq_-12). ^13^C NMR (CDCl_3_, δ ppm): 11.49 (C19); 12.00 (C29); 12.09 (C18); 17.43 (C1‴); 18.75 (C21); 19.07 (C26); 19.83 (C27); 21.23 (C11); 23.12 (C28); 24.23 (C15); 26.17 (C23); 28.24 (C6); 28.27 (C16); 29.22 (C25); 30.38 (C2); 31.70 (C7); 33.97 (C22); 34.45 (C4); 35.22 (C10); 35.45 (C8); 35.90 (C1); 36.18 (C20); 40.00 (C12); 42.63 (C13); 43.91 (C5); 45.89 (C24); 53.73 (C9); 56.22 (C17); 56.45 (C14); 102.90 (q, ^2^*J*_CF_ = 33.3, C5′); 112.55 (C3(3′); 121.85 (q, ^1^*J*_CF_ = 289.1, C1″). ^19^F NMR (CDCl_3_, δ ppm): −82.21 (F-1″).

#### 3.5.5. 3(*S*),5′(*S*)-3(3′)-Spiro-[(5′-trifluoromethyl-5′-methyl)-1′,2′,4′-trioxolane]-24-ethyl-5α-cholestan **3** β-Anti (d_4_)

^1^H NMR (CDCl_3_, δ ppm, *J* Hz): 0.65 (s, 3H, H-18); 0.67 (m, 1H, H-9); 0.80 (s, 3H, H-19); 0.82 (d, 3H, ^3^*J*_26-25_ = 6.8, H-26); 0.83 (d, 3H, ^3^*J*_27-25_ = 6.8, H-27); 0.84 (t, 3H, ^3^*J*_29-28_ = 7.5, H-29); 0.90 (d, 3H, ^3^*J*_21-20_ = 6.5, H-21); 0.94 (m, 1H, H_ax_-7); 0.94 (m, 1H, H-24); 1.01 (m, 1H, H-14); 1.03 (m, 1H, H_A_-22); 1.06 (m, 1H, H_β_-15); 1.13 (m, 1H, H-17); 1.15 (m, 1H, H_ax_-12); 1.17 (m, 2H, H-23); 1.22 (m, 1H, H_A_-28); 1.23 (m, 1H, H_ax_-1); 1.27 (m, 1H, H_β_-16); 1.28 (m, 1H, H_B_-28); 1.30 (m, 1H, H_ax_-11); 1.30 (m, 1H, H_ax_-6); 1.32 (m, 1H, H_eq_-6); 1.34 (m, 1H, H_B_-22); 1.35 (m, 1H, H-8); 1.37 (m, 1H, H-20); 1.47 (m, 1H, H-5); 1.49 (m, 1H, H_eq_-11); 1.58 (m, 1H, H_α_-15); 1.60 (m, 1H, Hax-4); 1.61 (m, 1H, H-1‴); 1.66 (m, 1H, H_eq_-4); 1.67 (m, 1H, H-25); 1.67 (m, 1H, H_eq_-1); 1.71 (m, 1H, H_eq_-7); 1.72 (m, 1H, H_ax_-2); 1.75 (m, 1H, H_eq_-2); 1.82 (m, 1H, H_α_-16); 1.98 (m, 1H, H_eq_-12). ^13^C NMR (CDCl_3_, δ ppm): 11.42 (C19); 12.00 (C29); 12.08 (C18); 17.50 (C1‴); 18.75 (C21); 19.07 (C26); 19.83 (C27); 21.23 (C11); 23.12 (C28); 24.23 (C15); 26.15 (C23); 27.11 (C2); 28.27 (C16); 28.42 (C6); 29.22 (C25); 31.88 (C7); 33.97 (C22); 35.45 (C8); 35.52 (C10); 35.62 (C1); 36.18 (C20); 37.89 (C4); 39.99 (C12); 42.61 (C13); 42.87 (C5); 45.89 (C24); 53.73 (C9); 56.18 (C17); 56.36 (C14); 103.38 (q, ^2^*J*_CF_ = 33.3, C5′); 112.51 (C3(3′)); 121.85 (q, ^1^*J*_CF_ = 289.1, C1″). ^19^F NMR (CDCl_3_, δ ppm): −82.02 (F-1″).

Crystallographic data for mixture of **3d_1_**, **3d_3_**: C_28_H_45_F_3_O_3_, FW = 486.64, Monoclinic, P2_1_, *a* = 13.513(3), *b* = 10.218(3), *c* = 22.715(6) Å, *β* = 101.68(1)°, V = 3071(1) Å^3^, Z = 4, Dcalc. = 1.052 g cm^−3^, μ (Mo-Kα) = 0.078 mm-1, F(000) = 1056, 30,230 measured reflections (θmax = 25.02°, completeness 99.5%), 10,777 independent (Rint = 0.074), 640 parameters (67 restraints), R1 = 0.0956 (for 5140 observed I > 2σ(I)), wR2 = 0.2574 (all data), GooF = 1.07, largest diff. peak and hole 0.50 and −0.23 e.A^−3^. CCDC 2512888 contain the supplementary crystallographic data for this paper. These data can be obtained from the Cambridge Crystallographic Data Centre via www.ccdc.cam.ac.uk/data_request/cif (accessed on 2 March 2026).

#### 3.5.6. 3(*R*,*S*),5′(*R*,*S*)-3(3′)-Spiro-[(5′-trifluoromethyl-5′-phenyl)-1′,2′,4′-trioxolane]-24-ethyl-5α-cholestan **4**

The yield 0.36 g (59%) as a mixture of four isomers. Anal. Calcd. for C_37_H_55_F_3_O_3_: C, 73.48 H, 9.17; F, 9.42. Found: C, 73.35; H, 9.04; F, 9.33.

#### 3.5.7. 3(*S*),5′(*R*)-3(3′)-Spiro-[(5′-trifluoromethyl-5′-phenyl)-1′,2′,4′-trioxolane]-24-ethyl-5α-cholestan **4** β-Syn (d_1_)

^1^H NMR (CDCl_3_, δ ppm, *J* Hz): 0.66 (s, 3H, H-18); 0.80 (m, 1H, H-9); 0.80 (s, 3H, H-19); 0.82 (d, 3H, ^3^*J*_26-25_ = 6.8, H-26); 0.84 (d, 3H, ^3^*J*_27-25_ = 6.8, H-27); 0.85 (t, 3H, ^3^*J*_29-28_ = 7.5, H-29); 0.92 (d, 3H, ^3^*J*_21-20_ = 6.5, H-21); 0.94 (m, 1H, H-24); 0.99 (m, 1H, H_ax_-7); 1.01 (m, 1H, H_A_-22); 1.03 (m, 1H, H-14); 1.04 (m, 1H, H_β_-15); 1.12 (m, 1H, H-17); 1.15 (m, 1H, H_ax_-12); 1.18 (m, 2H, H-23); 1.23 (m, 1H, H_A_-28); 1.23 (m, 1H, H_ax_-1); 1.26 (m, 1H, H_ax_-6); 1.26 (m, 1H, H_β_-16); 1.28 (m, 1H, H_B_-28); 1.32 (m, 1H, H_ax_-11); 1.34 (m, 1H, H_B_-22); 1.35 (m, 1H, H-20); 1.37 (m, 1H, H-8); 1.40 (m, 1H, H_eq_-6); 1.48 (m, 1H, H_eq_-11); 1.58 (m, 1H, H_α_-15); 1.60 (m, 1H, H_eq_-2); 1.64 (m, 1H, H_eq_-1); 1.65 (m, 1H, H_ax_-2); 1.66 (m, 1H, H-25); 1.66 (m, 1H, H-5); 1.67 (m, 1H, H_eq_-4); 1.69 (m, 1H, H_ax_-4); 1.71 (m, 1H, H_eq_-7); 1.85 (m, 1H, H_α_-16); 2.00 (m, 1H, H_eq_-12); 7.40 (t, 2H, ^3^*J* = 7.2, H*_meta_*); 7.42 (t, 1H, ^3^*J* = 7.2, H*_para_*); 7.57 (d, 2H, ^3^*J* = 7.2, H*_ortho_*). ^13^C NMR (CDCl_3_, δ ppm): 11.54 (C19); 12.05 (C29); 12.12 (C18); 18.81 (C21); 19.13 (C26); 19.87 (C27); 21.35 (C11); 23.18 (C28); 24.29 (C15); 26.30 (C23); 28.33 (C16); 28.57 (C6); 29.30 (C25); 30.28 (C2); 31.87 (C7); 33.55 (C4); 34.05 (C22); 35.43 (C1); 35.55 (C8); 35.67 (C10); 36.27 (C20); 40.08 (C12); 42.69 (C13); 44.04 (C5); 45.96 (C24); 53.90 (C9); 56.35 (C17); 56.52 (C14); 103.43 (q, ^2^*J*_FC_ = 33.1, C5′); 113.45 (C3(3′)); 121.75 (q, ^1^*J*_FC_ = 288.8, C1″); 126.63 (C*_ortho_*); 128.32 (C*_meta_*); 130.24 (C*_para_*); 132.18 (C*_q_*). ^19^F NMR (CDCl_3_, δ ppm): −79.92 (F-1″).

#### 3.5.8. 3(*S*),5′(*S*)-3(3′)-Spiro-[(5′-trifluoromethyl-5′-phenyl)-1′,2′,4′-trioxolane]-24-ethyl-5α-cholestan **4** β-Anti (d_2_)

^1^H NMR (CDCl_3_, δ ppm, *J* Hz): 0.66 (s, 3H, H-18); 0.80 (s, 3H, H-19); 0.82 (d, 3H, ^3^*J*_26-25_ = 6.8, H-26); 0.82 (m, 1H, H-9); 0.84 (d, 3H, ^3^*J*_27-25_ = 6.8, H-27); 0.85 (t, 3H, ^3^*J*_29-28_ = 7.5, H-29); 0.91 (m, 1H, H_ax_-7); 0.92 (d, 3H, ^3^*J*_21-20_ = 6.5, H-21); 0.94 (m, 1H, H-24); 1.01 (m, 1H, H_A_-22); 1.03 (m, 1H, H-14); 1.04 (m, 1H, H_β_-15); 1.07 (m, 1H, H_eq_-6); 1.12 (m, 1H, H-17); 1.14 (m, 1H, H_ax_-6); 1.15 (m, 1H, H_ax_-12); 1.18 (m, 2H, H-23); 1.23 (m, 1H, H_A_-28); 1.26 (m, 1H, H_β_-16); 1.28 (m, 1H, H_B_-28); 1.32 (m, 1H, H_ax_-11); 1.34 (m, 1H, H-8); 1.34 (m, 1H, H_B_-22); 1.35 (m, 1H, H-20); 1.40 (m, 1H, H_eq_-4); 1.45 (m, 1H, H_ax_-1); 1.48 (m, 1H, H_ax_-4); 1.51 (m, 1H, H-5); 1.53 (m, 1H, H_eq_-11); 1.58 (m, 1H, H_α_-15); 1.65 (m, 1H, H_eq_-7); 1.66 (m, 1H, H-25); 1.81 (m, 1H, H_ax_-2); 1.85 (m, 1H, H_α_-16); 1.86 (m, 1H, H_eq_-1); 1.93 (m, 1H, H_eq_-2); 1.97 (m, 1H, H_eq_-12); 7.40 (t, 2H, ^3^*J* = 7.2, H*_meta_*); 7.42 (t, 1H, ^3^*J* = 7.2, H*_para_*); 7.57 (d, 2H, ^3^*J* = 7.2, H*_ortho_*). ^13^C NMR (CDCl_3_, δ ppm): 11.51 (C19); 12.05 (C29); 12.12 (C18); 18.81 (C21); 19.13 (C26); 19.87 (C27); 21.34 (C11); 23.18 (C28); 24.29 (C15); 26.30 (C23); 27.49 (C2); 28.30 (C6); 28.33 (C16); 29.30 (C25); 32.01 (C7); 34.05 (C22); 35.50 (C8); 35.61 (C10); 35.98 (C1); 36.26 (C20); 36.57 (C4); 40.04 (C12); 42.69 (C13); 43.39 (C5); 45.96 (C24); 53.94 (C9); 56.35 (C17); 56.52 (C14); 103.64 (q, ^2^*J*_FC_ = 33.1, C5′); 113.45 (C3(3′)); 121.80 (q, ^1^*J*_FC_ = 288.8, C1″); 126.63 (C*_ortho_*); 128.35 (C*_meta_*); 130.24 (C*_para_*); 132.37 (C*_q_*). ^19^F NMR (CDCl_3_, δ ppm): −79.76 (F-1″).

#### 3.5.9. 3(*R*),5′(*S*)-3(3′)-Spiro-[(5′-trifluoromethyl-5′-phenyl)-1′,2′,4′-trioxolane]-24-ethyl-5α-cholestan **4** α-Syn (d_3_)

^1^H NMR (CDCl_3_, δ ppm, *J* Hz): 0.64 (s, 3H, H-18); 0.68 (m, 1H, H-9); 0.82 (d, 3H, ^3^*J*_26-25_ = 6.8, H-26); 0.83 (s, 3H, H-19); 0.84 (d, 3H, ^3^*J*_27-25_ = 6.8, H-27); 0.85 (t, 3H, ^3^*J*_29-28_ = 7.5, H-29); 0.86 (m, 1H, H_ax_-7); 0.92 (d, 3H, ^3^*J*_21-20_ = 6.5, H-21); 0.94 (m, 1H, H-24); 0.99 (m, 1H, H-14); 1.01 (m, 1H, H_A_-22); 1.02 (m, 1H, H_β_-15); 1.10 (m, 1H, H-17); 1.13 (m, 1H, H_ax_-1); 1.13 (m, 1H, H_ax_-12); 1.18 (m, 2H, H-23); 1.23 (m, 1H, H_A_-28); 1.24 (m, 1H, H_ax_-6); 1.26 (m, 1H, H_β_-16); 1.28 (m, 1H, H_B_-28); 1.29 (m, 1H, H_ax_-11); 1.32 (m, 1H, H-8); 1.33 (m, 1H, H-5); 1.34 (m, 1H, H_B_-22); 1.35 (m, 1H, H-20); 1.35 (m, 1H, H_eq_-6); 1.47 (m, 1H, H_eq_-11); 1.55 (m, 1H, H_α_-15); 1.56 (m, 1H, H_eq_-1); 1.66 (m, 1H, H-25); 1.68 (m, 1H, H_eq_-7); 1.70 (m, 1H, H_ax_-2); 1.71 (m, 1H, H_eq_-2); 1.78 (m, 1H, H_eq_-4); 1.85 (m, 1H, H_α_-16); 1.96 (m, 1H, H_eq_-12); 1.97 (m, 1H, H_ax_-4); 7.40 (t, 2H, ^3^*J* = 7.2, H*_meta_*); 7.42 (t, 1H, ^3^*J* = 7.2, H*_para_*); 7.55 (d, 2H, ^3^*J* = 7.2, H*_ortho_*). ^13^C NMR (CDCl_3_, δ ppm): 11.32 (C19); 12.05 (C29); 12.14 (C18); 18.81 (C21); 19.13 (C26); 19.87 (C27); 21.23 (C11); 23.18 (C28); 24.26 (C15); 26.22 (C23); 28.29 (C16); 28.48 (C6); 29.30 (C25); 29.99 (C2); 31.77 (C7); 34.01 (C22); 34.63 (C4); 35.33 (C10); 35.48 (C8); 35.65 (C1); 36.22 (C20); 39.99 (C12); 42.65 (C13); 44.00 (C5); 45.96 (C24); 53.77 (C9); 56.24 (C17); 56.40 (C14); 103.14 (q, ^2^*J*_FC_ = 33.1, C5′); 113.43 (C3(3′)); 121.80 (q, ^1^*J*_FC_ = 288.8, C1″); 126.71 (C*_ortho_*); 128.29 (C*_meta_*); 130.24 (C*_para_*); 132.22 (C*_q_*). ^19^F NMR (CDCl_3_, δ ppm): −79.75 (F-1″).

#### 3.5.10. 3(*R*),5′(*R*)-3(3′)-Spiro-[(5′-trifluoromethyl-5′-phenyl)-1′,2′,4′-trioxolane]-24-ethyl-5α-cholestan **4** α-Anti (d_4_)

^1^H NMR (CDCl_3_, δ ppm, *J* Hz): 0.64 (s, 3H, H-18); 0.68 (m, 1H, H-9); 0.82 (d, 3H, ^3^*J*_26-25_ = 6.8, H-26); 0.84 (d, 3H, ^3^*J*_27-25_ = 6.8, H-27); 0.84 (s, 3H, H-19); 0.85 (m, 1H, H_ax_-7); 0.85 (t, 3H, ^3^*J*_29-28_ = 7.5, H-29); 0.92 (d, 3H, ^3^*J*_21-20_ = 6.5, H-21); 0.94 (m, 1H, H-24); 0.99 (m, 1H, H-14); 1.01 (m, 1H, H_A_-22); 1.02 (m, 1H, H_β_-15); 1.10 (m, 1H, H-17); 1.10 (m, 1H, H_ax_-6); 1.13 (m, 1H, H_ax_-12); 1.13 (m, 1H, H_eq_-6); 1.15 (m, 1H, H_ax_-1); 1.18 (m, 2H, H-23); 1.23 (m, 1H, H_A_-28); 1.26 (m, 1H, H_β_-16); 1.28 (m, 1H, H_B_-28); 1.29 (m, 1H, H_ax_-11); 1.32 (m, 1H, H-5); 1.32 (m, 1H, H-8); 1.34 (m, 1H, H_B_-22); 1.35 (m, 1H, H-20); 1.47 (m, 1H, H_eq_-11); 1.47 (m, 1H, H_eq_-4); 1.55 (m, 1H, H_α_-15); 1.56 (m, 1H, H_ax_-4); 1.61 (m, 1H, H_eq_-7); 1.66 (m, 1H, H-25); 1.75 (m, 1H, H_eq_-1); 1.85 (m, 1H, H_α_-16); 1.97 (m, 1H, H_eq_-12); 2.02 (m, 1H, H_eq_-2); 2.11 (m, 1H, H_ax_-2); 7.40 (t, 2H, ^3^*J* = 7.2, H*_meta_*); 7.42 (t, 1H, ^3^*J* = 7.2, H*_para_*); 7.55 (d, 2H, ^3^*J* = 7.2, H*_ortho_*). ^13^C NMR (CDCl_3_, δ ppm): 11.32 (C19); 12.05 (C29); 12.14 (C18); 18.81 (C21); 19.13 (C26); 19.87 (C27); 21.26 (C11); 23.18 (C28); 24.26 (C15); 26.22 (C23); 28.15 (C6); 28.27 (C2); 28.29 (C16); 29.30 (C25); 31.77 (C7); 34.01 (C22); 35.24 (C10); 35.47 (C8); 35.94 (C1); 36.22 (C20); 36.47 (C4); 40.04 (C12); 42.65 (C13); 43.51 (C5); 45.96 (C24); 53.80 (C9); 56.24 (C17); 56.40 (C14); 103.08 (q, ^2^*J*_FC_ = 33.1, C5′); 113.43 (C3(3′)); 121.75 (q, ^1^*J*_FC_ = 288.8, C1″); 126.71 (C*_ortho_*); 128.33 (C*_meta_*); 130.24 (C*_para_*); 132.22 (C*_q_*).^19^F NMR (CDCl_3_, δ ppm): −79.68 (F-1″).

## 4. Conclusions

The novelty of this work lies in the integrated use of synthetic, crystallographic, spectroscopic, and computational methods to unambiguously determine the stereochemistry of a complex mixture of new spiro-1,2,4-trioxolanes with a β-sitostanone unit. The first synthesis of diastereomeric β-sitostanone spiro-1,2,4-trioxolanes by the Griesbaum co-ozonolysis reaction was achieved. X-ray diffraction analysis established that the isolated α-fraction of compound **3** is a crystallizing pair of diastereomers with the α-*anti* (3*R*,5′*R*) and α-*syn* (3*R*,5′*S*) configurations. The use of chemometric PLS2 analysis, through comparison of DFT-calculated and experimental ^13^C NMR chemical shifts, enabled objective and reproducible assignment of the stereochemical configuration to each diastereomer directly in the inseparable mixture. Unlike traditional methods (DP4+, MAEΔΔδ), the PLS2 approach not only verifies signal assignments but also allows the identification of diagnostic atoms (C2, C4, C5) and the statistically justified removal of structural outliers, thereby significantly enhancing the reliability of the assignment. The developed PLS2 methodology represents a powerful tool for the stereochemical analysis of complex diastereomeric mixtures, particularly in cases where chromatographic separation and X-ray diffraction data are unavailable, expanding the methodology for the analysis of complex peroxide-containing mixtures.

## Figures and Tables

**Table 1:** Boltzmann populations at 0 °C for different computational approaches and experimental distribution at 27 °C for compound **3**.

No.	Level of Theory	α-*Anti*	β-*Syn*	α-*Syn*	β-*Anti*	Bias	R^2^
	Experiment	0.339	0.271	0.220	0.169	-	-
1	B3LYP/6-31G	0.371	0.264	0.305	0.060	+0.014	0.75
2	B3LYP/6-31G(*d*)	0.342	0.186	0.330	0.143	−0.016	0.82
3	B3LYP/6-31G(*d*) + SMD (CH_2_Cl_2_)	0.473	0.128	0.255	0.144	+0.032	0.68
4	B3LYP/6-311G(*d*,*p*)	0.364	0.329	0.207	0.100	+0.015	0.80
5	B3LYP/6-311+G(*2d*,*p*)	0.424	0.273	0.189	0.115	−0.010	0.89
6	B3LYP/6-311+G(*2d*,*p*) + SMD (CH_2_Cl_2_)	0.490	0.100	0.198	0.212	+0.000	−2.44
7	M062X/6-311+G(*d*,*p*)	0.255	0.229	0.239	0.277	+0.045	−0.62
8	M062X/6-311+G(*d*,*p*) + SMD (CHCl_3_)	0.361	0.321	0.162	0.155	+0.008	0.85

## Data Availability

The original contributions presented in this study are included in the article/Supplementary Material. Further inquiries can be directed to the corresponding author.

## Supplementary Materials

The following supporting information can be downloaded at https://www.mdpi.com/article/10.3390/molecules31173105/s1, Table S1: Experimental ^13^C{^1^H} NMR chemical shifts for the diastereomeric mixtures of compounds **3** and **4**; Table S2: Computational approximations (protocols) for calculating ^13^C chemical shifts; Table S3: Quantitative energetic data for the four diastereomers of compound **3**; Table S4: DFT-calculated 13C NMR chemical shifts for the diastereomers of compound **3**; Table S5: DFT-calculated 13C NMR chemical shifts for the diastereomers of compound **4**; Table S6: Statistical parameters of refined PLS2 models for different computational protocols based on the diastereomeric mixture of compound **3**; Table S7: Refined PLS2-model PC-02 loadings for different computational protocols based on the diastereomeric mixture of compound **3**; Table S8: Summary of PLS2-based stereochemical assignment for compound **3** and compound **4** across different computational protocols; Table S9: Identification of structural outliers for compound **4** across different computational protocols using the Tukey criterion (Q3 + 1.5 × IQR); Table S10: PLS2 PC-02 loadings for different computational protocols based on the diastereomeric mixture of compound **4**; Table S11: Statistical parameters of refined PLS2 models for different computational protocols based on the diastereomeric mixture of compound **4**; Figure S1: Crystal packing of α-isomers mixture of compound **3**; Figure S2: PLS2 analysis of compound **3**; Figure S3: PLS2 analysis of compound **3** after removal of structural outliers; Figure S4: PLS2 analysis of compound **4**; Figure S5. PLS2 analysis of compound **4** after removal of structural outliers; Figures S6–S56: The 2D NMR spectra of compounds **2**–**4**. Figures S57 and S58. HRMS data of compounds **3**d_1_, **3**d_3_ and **3**d_2_, **3**d_4_.
