# Supplementary material for: Synthesis and Stereochemical Assignment of Diastereomeric Steroidal Spiro-1,2,4-trioxolanes with Sitostanone Motif Using PLS2 Chemometric Analysis of DFT-Calculated and Experimental 13C NMR Data
Source: Molecules. 2026 Sep 4;31(17):3105. doi: 10.3390/molecules31173105 (PMC13567592; doi:10.3390/molecules31173105)
Supplement: Supplementary file 1 [file molecules-31-03105-s001.zip › molecules-4495062-supplementary.pdf]

Supplementary Information

Synthesis and Stereochemical Assignment of Diastereomeric Steroidal Spiro-1,2,4-trioxolanes with Sitostanone Motif Using PLS2 Chemometric Analysis of DFT-Calculated and Experimental ^{13}C NMR Data †

Alexander Lobov [1], Anastasiya Kerimova [1], Irina Smirnova [1], Dmitriy Polovyanenko [2], Irina Bagryanskaya [2] and Oxana Kazakova [1,*]

[1] Ufa Institute of Chemistry of the Ufa Federal Research Centre of the Russian Academy of Sciences, 71, pr. Oktyabrya, 450054 Ufa, Russia; lobovan@anrb.ru (A.L.); ana.orgchem@gmail.com (A.K.); si8081@yandex.ru (I.S.)
[2] N. N. Vorozhtsov Novosibirsk Institute of Organic Chemistry SB RAS, 630090 Novosibirsk, Russia; dpolo@nioch.nsc.ru (D.P.); bagryan@nioch.nsc.ru (I.B.)
* Correspondence: obf@anrb.ru
† Dedicated to the memory of our colleague Leonid Spirikhin for his significant contribution to the structure elucidation of natural compounds.

Abstract

The synthesis and multi-step spectroscopic characterization of diastereomeric 3-spiro-1,2,4-trioxolanes obtained by Griesbaum co-ozonolysis of sitostanone O-methyl oxime with two types of fluorinated ketones are reported. The reaction furnished four possible diastereomers that differ in the α/β orientation of the peroxide bridge relative to the steroid A-ring and in the *syn*/*anti* orientation of the trifluoromethyl group at C5′. A chemometric PLS2 approach, linking experimental and DFT-calculated ^{13}C chemical shifts, was used for the objective stereochemical assignment of each diastereomer in the inseparable mixtures. Single-crystal X-ray analysis of the isolated 3*R*,5′*R* and 3*R*,5′*S* stereoisomeric pair (α-*anti* and α-*syn* ozonides) provided unambiguous absolute configurations that validated the stereochemical assignments obtained by the combined NMR/DFT/PLS2 approach. This integrated synthetic, crystallographic, spectroscopic, and chemometric methodology provides reliable configurational assignment in complex spiro-peroxide mixtures and expands the analytical toolkit for such systems.

Keywords: 3-*spiro*-1,2,4-trioxolane; steroids; sitostanone; Griesbaum co-ozonolysis; X-ray analysis; high-resolution NMR; DFT; chemometrics

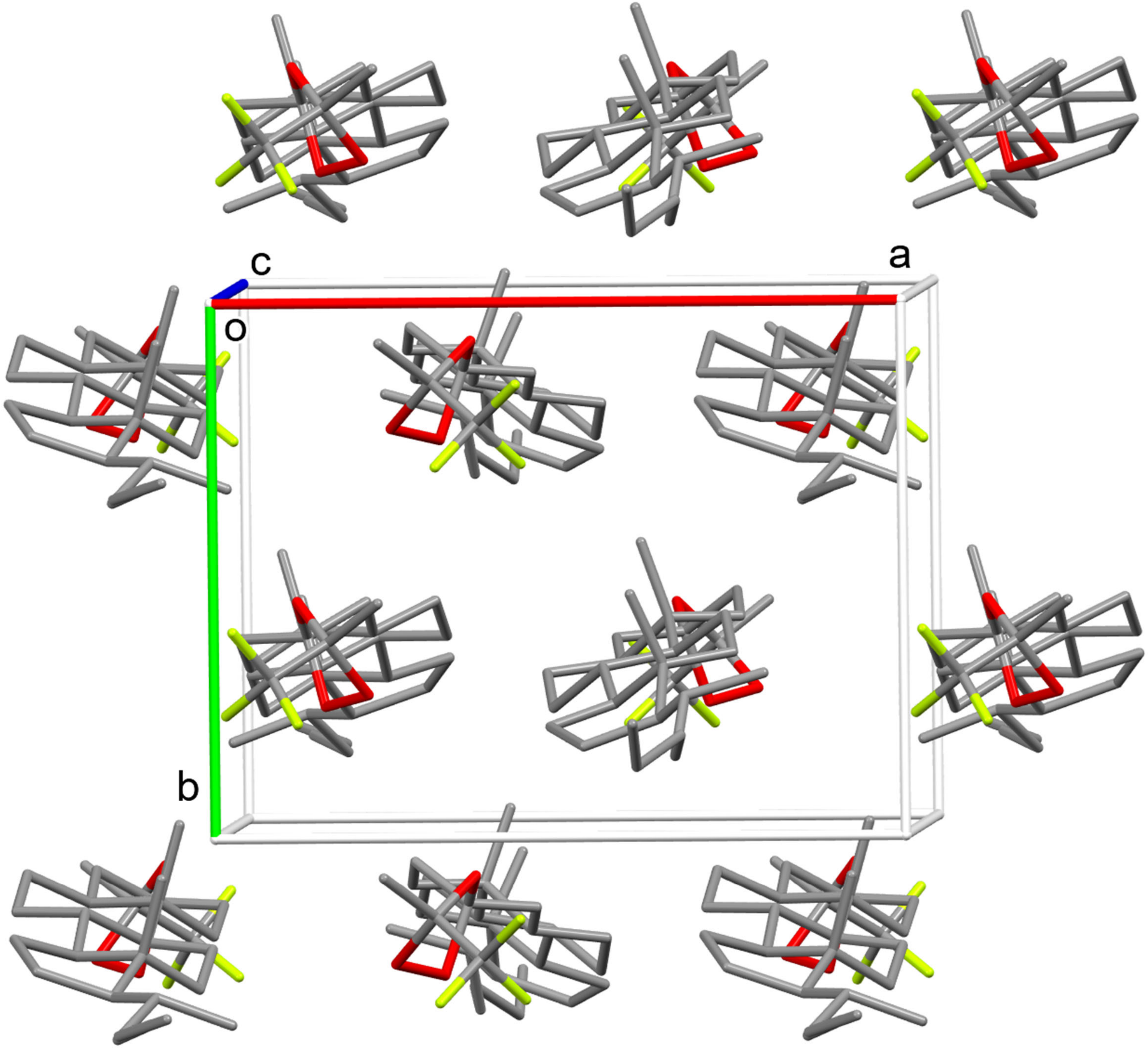

Figure S1. Crystal packing of α-isomers mixture of compound **3** along crystallographic axis c. The H atoms are omitted for clarity

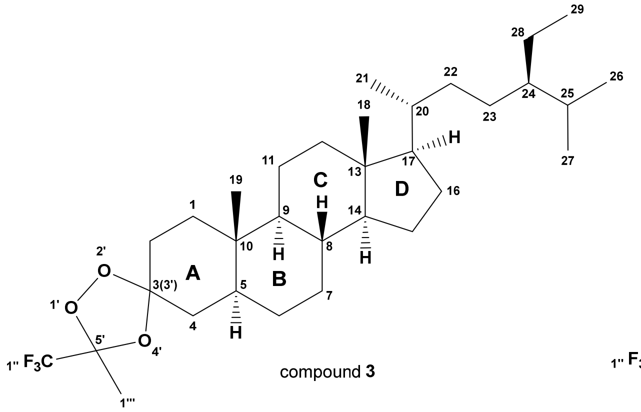

compound **3**

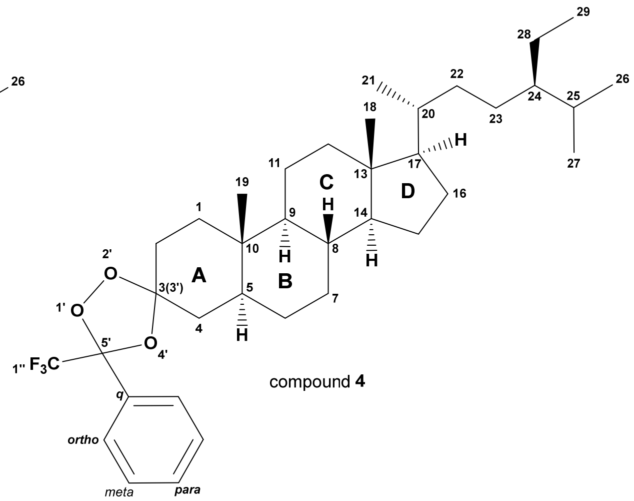

compound **4**

Table S1. Experimental $^{13}C\{^1H\}$ NMR chemical shifts (δ, ppm) for the diastereomeric mixtures (d_1-d_4) of compounds **3** and **4** in $CDCl_3$

compound **3**				
№	d_1	d_2	d_3	d_4
C1	35.85	35.17	35.43	35.90
C2	28.01	31.35	30.28	27.11
C3(3')	112.51	112.61	113.45	112.51
C4	37.02	33.34	33.55	37.89
C5	43.34	43.97	44.04	42.87
C6	28.48	28.24	28.57	28.42
C7	31.78	31.78	31.87	31.88
C8	35.45	35.45	35.55	35.45
C9	53.75	53.75	53.90	53.73
C10	35.12	35.59	35.67	35.52
C11	21.16	21.31	21.35	21.23
C12	39.93	39.95	40.08	39.99
C13	42.61	42.63	42.69	42.61
C14	56.36	56.44	56.52	56.36
C15	24.23	24.23	24.29	24.23
C16	28.27	28.27	28.33	28.27
C17	56.18	56.22	56.35	56.18
C18	12.08	12.09	12.12	12.08
C19	11.24	11.22	11.54	11.42
C20	36.18	36.18	36.27	36.18
C21	18.75	18.75	18.81	18.75
C22	33.97	33.97	34.05	33.97
C23	26.15	26.17	26.30	26.15
C24	45.89	45.89	45.96	45.89
C25	29.22	29.22	29.30	29.22
C26	19.07	19.07	19.13	19.07
C27	19.83	19.83	19.87	19.83
C28	23.12	23.12	23.18	23.12
C29	12.00	12.00	12.05	12.00
C5'	102.85	103.22	103.43	103.38
C1''	121.85	121.85	121.75	121.85
C1'''	17.38	17.35	132.18	17.50

compound **4**				
№	d_1	d_2	d_3	d_4
C1	35.43	35.98	35.65	35.94
C2	30.28	27.49	29.99	28.27
C3(3')	113.45	113.45	113.43	113.43
C4	33.55	36.57	34.63	36.47
C5	44.04	43.39	44.00	43.51
C6	28.57	28.30	28.48	28.15
C7	31.87	32.01	31.77	31.77
C8	35.55	35.50	35.48	35.47
C9	53.90	53.94	53.77	53.80
C10	35.67	35.61	35.33	35.24
C11	21.35	21.34	21.23	21.26
C12	40.08	40.04	39.99	40.04
C13	42.69	42.69	42.65	42.65
C14	56.52	56.52	56.40	56.40
C15	24.29	24.29	24.26	24.26
C16	28.33	28.33	28.29	28.29
C17	56.35	56.35	56.24	56.24
C18	12.12	12.12	12.14	12.14
C19	11.54	11.51	11.32	11.32
C20	36.27	36.26	36.22	36.22
C21	18.81	18.81	18.81	18.81
C22	34.05	34.05	34.01	34.01
C23	26.30	26.30	26.22	26.22
C24	45.96	45.96	45.96	45.96
C25	29.30	29.30	29.30	29.30
C26	19.13	19.13	19.13	19.13
C27	19.87	19.87	19.87	19.87
C28	23.18	23.18	23.18	23.18
C29	12.05	12.05	12.05	12.05
C5'	103.43	103.64	103.14	103.08
C1''	121.75	121.80	121.80	121.75
C_q	132.18	132.37	132.22	132.22
C_{ortho}	126.63	126.63	126.71	126.71
C_{meta}	128.32	128.35	128.29	128.33
C_{para}	130.24	130.24	130.24	130.24

Table S2. Computational approximations (protocols) for calculating ^{13}C chemical shifts.

№	Geometry Optimization	NMR Calculation	Notes
**1**	B3LYP/6-31G	B3LYP/6-31G GIAO	Base level
**2**	B3LYP/6-31G(*d*)	B3LYP/6-311+G(*2d*,*p*) GIAO	Recommended for routine calculations (good balance of accuracy and cost; R^2 = 0.954, RMSEC = 2.812)
**2A**	B3LYP/6-31G(*d*)	wB97XD/Def2SVP PCM($CHCl_3$) GIAO	Recommended for maximum accuracy (best overall performance: R^2 = 0.970, RMSEC = 2.266, Bias = +0.241, Slope = 0.852)
**3**	B3LYP/6-31G(d) SMD (CH_2Cl_2)	B3LYP/6-311+G(*2d*,*p*) GIAO	Effect of solvation on geometry optimization (GIAO)
**4**	B3LYP/6-311G(*d*,*p*)	B3LYP/6-311G(*d*,*p*) GIAO	Effect of extended Pople basis set (GIAO)
**4A**	B3LYP/6-311G(*d*,*p*)	MPW1PW91/6-311+G(*2d*,*p*) CSGT	CSGT vs GIAO on the same basis set
**5**	B3LYP/6-311+G(*2d*,*p*)	MPW1PW91/6-311+G(*2d*,*p*) CSGT	High accuracy alternative (R^2 = 0.959, RMSEC = 2.661, Slope = 0.858) but higher computational cost
**6**	B3LYP/6-311+G(*2d*,*p*) SMD (CH_2Cl_2)	MPW1PW91/6-311+G(*2d*,*p*) CSGT	Solvation improves correlation (R^2 = 0.966) but introduces systematic bias (Slope = 0.785); only for specific solvent calculations
**7**	M062X/6-311+G(*d*,*p*)	MPW1PW91/6-311+G(*2d*,*p*) CSGT	M062X geometry optimization (gas phase)
**8**	M062X/6-311+G(*d*,*p*) SMD ($CHCl_3$)	MPW1PW91/6-311+G(*2d*,*p*) CSGT	M062X geometry + solvation (SMD, $CHCl_3$)
**8A**	M062X/6-311+G(*d*,*p*) SMD ($CHCl_3$)	M062X/6-311+G(*d*,*p*) GIAO	Full consistency: M062X for geometry, solvation, and NMR (GIAO)

Table S3. Quantitative energetic data for the four diastereomers of compound **3** at different computational protocols (1–8)

	Structure	E	ZPE	H	qh-H	T.S	T.qh-S	G(T)	qh-G(T)	Boltz
Protocol 1	**α-*anti***	-1776.015522	0.818877	-1775.162695	-1775.168433	0.093468	0.086928	-1775.256163	-1775.255361	0.371
	**α-*syn***	-1776.015471	0.818897	-1775.162636	-1775.168399	0.093293	0.086791	-1775.255929	-1775.255190	0.305
	**β-*syn***	-1776.015081	0.818894	-1775.162202	-1775.168005	0.093760	0.087061	-1775.255962	-1775.255066	0.264
	**β-*anti***	-1776.015078	0.819477	-1775.161873	-1775.167577	0.092439	0.086208	-1775.254312	-1775.253785	0.060
Protocol 2	**α-*anti***	-1776.473856	0.797815	-1775.641382	-1775.647185	0.095131	0.088265	-1775.736513	-1775.735450	0.342
	**α-*syn***	-1776.473817	0.797803	-1775.641349	-1775.647134	0.095178	0.088285	-1775.736527	-1775.735419	0.330
	**β-*syn***	-1776.473368	0.797823	-1775.640909	-1775.646695	0.095082	0.088229	-1775.735991	-1775.734923	0.186
	**β-*anti***	-1776.473305	0.797900	-1775.640796	-1775.646584	0.094862	0.088111	-1775.735657	-1775.734695	0.143
Protocol 3	**α-*anti***	-1776.497347	0.795362	-1775.667184	-1775.673037	0.095887	0.088722	-1775.763071	-1775.761759	0.473
	**α-*syn***	-1776.497342	0.795577	-1775.667064	-1775.672858	0.095379	0.088368	-1775.762443	-1775.761226	0.255
	**β-*syn***	-1776.496995	0.795680	-1775.666673	-1775.672445	0.095027	0.088186	-1775.761700	-1775.760630	0.128
	**β-*anti***	-1776.496901	0.795633	-1775.666595	-1775.672407	0.095267	0.088320	-1775.761862	-1775.760727	0.144
Protocol 4	**α-*anti***	-1776.946097	0.809164	-1776.102807	-1776.108594	0.093731	0.087176	-1776.196538	-1776.195770	0.364
	**α-*syn***	-1776.946096	0.809410	-1776.102616	-1776.108401	0.093293	0.086879	-1776.195909	-1776.195280	0.207
	**β-*syn***	-1776.945454	0.809010	-1776.102195	-1776.108115	0.094481	0.087567	-1776.196676	-1776.195681	0.329
	**β-*anti***	-1776.945493	0.809456	-1776.101996	-1776.107805	0.093271	0.086847	-1776.195267	-1776.194653	0.100
Protocol 5	**α-*anti***	-1777.005980	0.808555	-1776.163209	-1776.169034	0.094036	0.087400	-1776.257245	-1776.256434	0.424
	**α-*syn***	-1777.005970	0.808898	-1776.162957	-1776.168752	0.093423	0.086983	-1776.256380	-1776.255735	0.189
	**β-*syn***	-1777.005378	0.808581	-1776.162526	-1776.168453	0.094503	0.087601	-1776.257029	-1776.256054	0.273
	**β-*anti***	-1777.005424	0.808870	-1776.162439	-1776.168277	0.093499	0.087026	-1776.255938	-1776.255303	0.115
Protocol 6	**α-*anti***	-1777.032256	0.805601	-1776.192248	-1776.198220	0.095471	0.088137	-1776.287719	-1776.286357	0.490
	**α-*syn***	-1777.032242	0.805821	-1776.192131	-1776.198005	0.094510	0.087569	-1776.286641	-1776.285574	0.198
	**β-*syn***	-1777.031919	0.805982	-1776.191713	-1776.197564	0.094260	0.087414	-1776.285973	-1776.284977	0.100
	**β-*anti***	-1777.031828	0.805618	-1776.191838	-1776.197762	0.094854	0.087869	-1776.286692	-1776.285631	0.212
Protocol 7	**α-*anti***	-1776.247183	0.794478	-1775.418493	-1775.424033	0.093728	0.087200	-1775.512221	-1775.511233	0.255
	**α-*syn***	-1776.247228	0.794538	-1775.418486	-1775.424022	0.093702	0.087157	-1775.512187	-1775.511179	0.239
	**β-*syn***	-1776.246853	0.794401	-1775.418209	-1775.423782	0.094027	0.087359	-1775.512236	-1775.511141	0.229
	**β-*anti***	-1776.246853	0.794326	-1775.418267	-1775.423849	0.094158	0.087457	-1775.512425	-1775.511306	0.277
Protocol 8	**α-*anti***	-1776.273503	0.791992	-1775.447278	-1775.452811	0.093681	0.087180	-1775.540959	-1775.539991	0.361
	**α-*syn***	-1776.273559	0.792530	-1775.446919	-1775.452322	0.093442	0.086979	-1775.540360	-1775.539301	0.162
	**β-*syn***	-1776.273213	0.792045	-1775.446862	-1775.452446	0.094140	0.087445	-1775.541002	-1775.539890	0.321
	**β-*anti***	-1776.273261	0.792326	-1775.446772	-1775.452200	0.093459	0.087062	-1775.540230	-1775.539262	0.155

All values are given in Hartree. E — absolute energy; ZPE — zero-point vibrational energy; H and qh-H — enthalpic terms; T·S and T·qh-S — entropic terms; G(T) and qh-G(T) — Gibbs free energies; Boltz — Boltzmann populations at 0 °C

Table S4. DFT-calculated ^{13}C NMR chemical shifts (δ, ppm) for the four diastereomers (α-*anti*, β-*syn*, α-*syn*, and β-*anti*) of compound **3** obtained at different computational protocols and used for PLS2 model construction

	Protocol 1				Protocol 2					Protocol 2A				Protocol 3			
№	**α-*anti***	**β-*syn***	**α-*syn***	**β-*anti***	**α-*anti***	**β-*syn***	**α-*syn***	**β-*anti***	**α-*anti***	**α-*anti***	**β-*syn***	**α-*syn***	**β-*anti***	**α-*anti***	**β-*syn***	**α-*syn***	**β-*anti***
C1	38.41	38.15	38.49	39.43	39.91	39.32	39.76	40.28	39.91	36.51	36.24	36.56	36.81	39.93	39.28	39.71	40.13
C2	29.78	34.01	33.05	28.98	32.44	35.65	34.33	31.01	32.44	29.29	32.16	31.29	28.45	32.16	35.37	34.00	30.86
C3(3')	126.11	126.20	126.20	126.35	119.26	119.37	119.36	119.39	119.26	113.18	113.33	113.13	113.14	120.28	120.29	120.34	120.25
C4	39.49	35.07	35.93	40.36	41.20	37.18	38.53	42.08	41.20	37.92	34.79	35.76	38.69	40.90	36.93	38.28	41.76
C5	46.20	47.73	46.87	45.89	48.27	49.16	49.04	48.09	48.27	43.92	44.54	44.10	43.38	48.23	49.09	49.00	47.67
C6	30.43	30.66	30.54	30.44	32.58	32.57	32.78	32.78	32.58	28.72	28.80	28.78	28.71	32.55	32.58	32.74	32.61
C7	33.73	33.75	33.74	33.95	36.12	36.11	36.10	36.29	36.12	32.31	32.35	32.21	32.40	36.00	35.99	35.98	36.38
C8	38.85	38.90	38.86	38.92	40.96	40.96	40.90	40.86	40.96	36.33	36.32	36.26	36.30	40.80	40.83	40.77	40.74
C9	56.29	56.53	56.30	56.40	59.52	59.36	59.04	59.23	59.52	54.37	54.43	54.30	54.46	59.46	59.23	58.91	59.25
C10	41.15	41.76	41.42	41.50	41.95	41.68	42.19	42.79	41.95	37.35	37.80	37.44	37.69	41.82	41.52	42.08	42.20
C11	24.29	24.62	24.42	24.33	25.16	25.33	25.19	25.16	25.16	22.56	22.69	22.62	22.62	25.07	25.25	25.11	25.19
C12	41.72	41.94	41.63	41.63	44.43	44.60	44.45	44.42	44.43	40.31	40.35	40.28	40.30	44.44	44.56	44.42	44.30
C13	48.73	48.94	49.06	48.92	49.57	49.60	49.73	49.60	49.57	44.49	44.53	44.52	44.55	49.47	49.49	49.65	49.27
C14	59.03	59.13	59.21	59.31	61.70	61.60	61.49	61.66	61.70	57.51	57.51	57.50	57.54	61.59	61.42	61.31	61.56
C15	27.79	27.78	27.80	27.87	28.03	28.07	27.95	28.10	28.03	25.42	25.42	25.42	25.45	27.94	28.00	27.87	28.01
C16	32.94	33.07	33.00	33.10	33.07	33.02	33.01	33.05	33.07	30.37	30.42	30.42	30.40	32.99	32.91	32.95	33.09
C17	59.85	60.05	60.12	60.12	62.40	62.48	62.29	62.43	62.40	57.83	57.93	57.82	57.85	62.22	62.28	62.06	62.26
C18	14.03	14.28	14.37	14.23	12.30	12.35	12.44	12.40	12.30	13.83	13.83	13.83	13.84	12.29	12.36	12.43	12.45
C19	13.25	13.65	13.39	13.44	11.65	11.83	11.62	11.77	11.65	13.06	13.30	13.02	13.17	11.64	11.82	11.59	11.74
C20	40.95	40.95	40.89	40.85	43.60	43.70	43.71	43.63	43.60	38.97	38.89	38.94	38.96	43.52	43.61	43.67	43.60
C21	19.66	19.32	19.42	19.44	19.13	19.17	19.19	19.15	19.13	18.96	18.86	18.98	18.96	19.06	19.21	19.17	19.19
C22	37.86	37.89	37.89	37.88	40.03	39.92	40.09	40.08	40.03	36.54	36.49	36.54	36.57	39.97	39.82	39.95	39.93
C23	36.34	35.80	35.80	35.99	37.71	37.89	37.58	37.52	37.71	34.65	34.68	34.71	34.72	37.61	37.90	37.57	37.76
C24	48.62	48.92	49.01	48.95	52.46	52.13	52.57	52.57	52.46	46.17	46.20	46.24	46.20	52.41	52.10	52.54	52.25
C25	31.35	31.32	31.49	31.38	33.88	33.32	33.76	33.79	33.88	28.55	28.68	28.61	28.62	33.71	33.14	33.49	33.59
C26	16.41	16.41	16.23	16.19	15.68	15.63	15.52	15.55	15.68	15.90	15.88	15.86	15.91	15.66	15.59	15.48	15.60
C27	25.12	25.35	25.39	25.34	25.70	25.66	25.71	25.74	25.70	24.71	24.74	24.72	24.74	25.72	25.69	25.74	25.66
C28	32.12	32.36	32.06	32.00	33.97	34.00	34.02	33.99	33.97	30.89	30.96	30.89	30.89	33.87	33.86	33.91	34.00
C29	14.67	14.70	14.83	14.80	14.58	14.55	14.57	14.59	14.58	14.31	14.23	14.27	14.26	14.57	14.54	14.55	14.56
C5'	111.91	112.30	111.94	112.38	110.08	110.21	110.08	110.49	110.08	105.19	105.36	105.13	105.53	109.88	110.14	109.90	110.29
C1''	142.76	142.69	142.66	142.71	133.63	133.69	133.64	133.66	133.63	130.13	130.05	129.98	130.07	133.90	133.94	133.91	133.96
C1'''	18.18	18.25	18.08	18.20	18.39	18.34	18.53	18.48	18.39	18.34	18.31	18.34	18.34	17.990	18.06	18.177	18.07

The calculated ^{13}C NMR chemical shifts were referenced to TMS using the absolute shielding constants σ(TMS) calculated at the same level of theory for each protocol: Protocol 1 — 194.37 ppm; Protocol 2 — 182.48 ppm; Protocol 2A — 195.12 ppm; Protocol 3 — 182.33 ppm

Table S4. DFT-calculated ^{13}C NMR chemical shifts (δ, ppm) for the four diastereomers (α-*anti*, β-*syn*, α-*syn*, and β-*anti*) of compound **3** obtained at different computational protocols and used for PLS2 model construction

	Protocol 4				Protocol 4A					Protocol 5				Protocol 6			
№	**α-*anti***	**β-*syn***	**α-*syn***	**β-*anti***	**α-*anti***	**β-*syn***	**α-*syn***	**β-*anti***	**α-*anti***	**α-*anti***	**β-*syn***	**α-*syn***	**β-*anti***	**α-*anti***	**β-*syn***	**α-*syn***	**β-*anti***
C1	40.61	40.19	40.65	41.09	38.30	37.86	38.23	38.56	38.30	38.40	37.92	38.29	38.51	38.32	37.64	38.07	38.50
C2	32.79	36.06	34.75	31.54	30.69	34.02	32.72	29.57	30.69	30.71	34.01	32.69	29.55	30.26	33.59	32.27	29.10
C3(3')	119.23	119.24	119.20	119.37	117.19	117.24	117.19	117.20	117.19	117.16	117.12	117.17	117.09	118.26	118.17	118.31	118.11
C4	41.34	37.86	39.05	42.54	39.17	35.67	36.80	40.26	39.17	39.16	35.69	36.84	40.29	38.71	35.27	36.49	39.93
C5	48.42	49.35	49.15	48.22	45.94	46.78	46.45	45.58	45.94	45.83	46.62	46.38	45.47	45.80	46.50	46.32	45.27
C6	33.03	33.19	33.13	33.12	31.00	31.12	31.07	31.14	31.00	31.07	31.19	31.13	31.24	31.02	31.14	31.09	31.24
C7	36.56	36.57	36.60	36.86	34.38	34.34	34.33	34.64	34.38	34.39	34.34	34.33	34.64	34.26	34.28	34.15	34.36
C8	41.08	41.21	41.02	41.01	38.80	38.88	38.83	38.86	38.80	38.66	38.72	38.68	38.72	38.50	38.55	38.48	38.56
C9	59.81	59.95	59.80	59.96	56.70	56.87	56.82	57.03	56.70	56.67	56.81	56.80	56.98	56.57	56.54	56.46	56.61
C10	42.02	42.74	42.46	42.71	39.99	40.61	40.09	40.46	39.99	39.79	40.38	39.89	40.22	39.60	40.13	39.75	40.06
C11	25.93	26.25	25.96	25.87	23.97	24.24	24.08	24.03	23.97	23.97	24.23	24.08	24.01	23.80	23.99	23.91	23.90
C12	44.96	45.04	44.68	44.80	42.30	42.56	42.35	42.39	42.30	42.39	42.63	42.43	42.45	42.39	42.46	42.40	42.41
C13	50.10	50.22	50.17	50.18	47.56	47.66	47.68	47.63	47.56	47.36	47.47	47.50	47.44	47.32	47.31	47.33	47.35
C14	62.02	62.04	62.14	62.37	59.14	59.24	59.13	59.30	59.14	59.07	59.16	59.06	59.22	58.92	58.83	58.86	58.92
C15	28.82	28.85	28.71	28.77	26.89	26.93	26.89	26.93	26.89	26.88	26.92	26.88	26.91	26.76	26.76	26.77	26.77
C16	33.59	33.74	33.61	33.77	31.61	31.54	31.48	31.60	31.61	31.61	31.54	31.48	31.59	31.50	31.46	31.51	31.51
C17	62.51	62.73	62.82	62.98	59.88	59.95	59.98	59.98	59.88	59.92	59.94	59.97	59.99	59.63	59.74	59.56	59.68
C18	12.91	13.34	13.34	13.34	11.97	12.07	12.07	12.05	11.97	12.07	12.15	12.16	12.15	12.08	12.13	12.07	12.08
C19	12.51	12.87	12.72	12.74	11.49	11.79	11.51	11.59	11.49	11.58	11.87	11.61	11.62	11.47	11.70	11.48	11.60
C20	43.36	43.46	43.32	43.28	41.30	41.33	41.18	41.27	41.30	41.20	41.26	41.12	41.18	41.12	41.05	41.20	41.14
C21	20.11	19.84	19.97	19.89	19.07	18.92	18.90	18.89	19.07	19.12	19.02	19.00	18.98	18.95	18.97	19.02	18.95
C22	40.62	40.99	40.77	40.67	38.49	38.46	38.37	38.34	38.49	38.53	38.50	38.41	38.36	38.29	38.19	38.22	38.25
C23	38.84	38.24	38.24	38.30	36.21	36.04	36.09	36.18	36.21	36.26	36.12	36.18	36.26	36.13	36.21	36.24	36.17
C24	52.20	52.71	52.68	52.45	50.11	50.17	50.02	49.89	50.11	49.98	50.13	49.96	49.80	49.86	49.77	49.85	49.77
C25	33.42	33.51	33.70	33.82	32.01	32.07	31.92	32.01	32.01	31.98	31.98	31.84	31.93	31.78	31.73	31.68	31.81
C26	16.46	16.33	16.04	16.16	15.54	15.62	15.49	15.54	15.54	15.67	15.74	15.61	15.67	15.60	15.58	15.52	15.60
C27	26.10	26.25	26.43	26.38	25.44	25.48	25.44	25.41	25.44	25.58	25.63	25.59	25.57	25.62	25.65	25.59	25.62
C28	34.65	34.59	34.07	34.11	32.55	32.73	32.58	32.64	32.55	32.66	32.83	32.68	32.76	32.61	32.59	32.51	32.59
C29	15.18	15.07	15.38	15.40	14.42	14.52	14.44	14.45	14.42	14.54	14.63	14.55	14.56	14.55	14.53	14.51	14.55
C5'	108.89	109.22	109.10	109.42	107.37	107.64	107.44	107.77	107.37	107.25	107.68	107.33	107.81	106.98	107.56	107.06	107.71
C1''	132.42	132.39	132.34	132.42	131.12	131.20	131.14	131.20	131.12	131.16	131.22	131.17	131.22	131.50	131.55	131.52	131.53
C1'''	19.37	19.36	19.38	19.29	18.17	18.16	18.23	18.14	18.17	18.38	18.39	18.43	18.39	18.09	18.17	18.10	18.19

The calculated ^{13}C NMR chemical shifts were referenced to TMS using the absolute shielding constants σ(TMS) calculated at the same level of theory for each protocol: Protocol 4 — 184.41 ppm; Protocol 4A — 187.16 ppm; Protocol 5 — 187.49 ppm; Protocol 6 — 187.27ppm

Table S4. DFT-calculated ^{13}C NMR chemical shifts (δ, ppm) for the four diastereomers (α-*anti*, β-*syn*, α-*syn*, and β-*anti*) of compound **3** obtained at different computational protocols and used for PLS2 model construction

	Protocol 7				Protocol 8					Protocol 8A			
№	**α-*anti***	**β-*syn***	**α-*syn***	**β-*anti***	**α-*anti***	**β-*syn***	**α-*syn***	**β-*anti***	**α-*anti***	**α-*anti***	**β-*syn***	**α-*syn***	**β-*anti***
C1	38.58	38.34	38.41	38.82	38.39	37.91	38.28	38.50	38.39	39.64	39.53	40.05	39.95
C2	30.54	33.48	32.41	29.38	30.69	34.00	32.67	29.53	30.69	32.15	35.08	34.33	31.49
C3(3')	114.95	115.04	114.96	115.06	117.14	117.11	117.15	117.08	117.14	120.49	120.35	120.57	120.86
C4	39.12	35.56	36.81	40.03	39.14	35.67	36.83	40.27	39.14	41.72	37.45	39.24	42.59
C5	45.79	46.28	46.09	45.27	45.81	46.60	46.36	45.46	45.81	47.37	48.23	47.60	46.83
C6	31.46	31.52	31.48	31.54	31.06	31.17	31.12	31.22	31.06	31.93	31.43	31.26	31.81
C7	34.88	34.71	34.88	34.98	34.37	34.32	34.32	34.62	34.37	35.35	35.34	35.28	35.64
C8	38.62	38.63	38.54	38.52	38.65	38.71	38.67	38.70	38.65	40.00	40.10	40.33	40.57
C9	56.28	56.58	56.48	56.65	56.66	56.80	56.79	56.97	56.66	59.47	59.62	59.65	59.56
C10	38.36	38.88	38.40	38.81	39.78	40.36	39.88	40.21	39.78	40.67	41.13	40.64	40.82
C11	24.07	24.37	24.11	24.16	23.95	24.22	24.07	24.00	23.95	24.38	24.18	23.94	23.85
C12	42.49	42.72	42.55	42.69	42.38	42.61	42.42	42.43	42.38	44.17	44.14	43.62	43.71
C13	45.59	45.85	45.81	45.71	47.35	47.45	47.48	47.42	47.35	49.11	49.40	49.05	48.74
C14	59.07	59.14	59.16	59.36	59.06	59.15	59.05	59.21	59.06	62.51	62.77	62.58	62.91
C15	27.40	27.27	27.35	27.32	26.87	26.91	26.86	26.90	26.87	27.59	27.68	27.64	27.53
C16	32.42	32.12	32.12	32.32	31.59	31.53	31.47	31.58	31.59	33.17	33.12	32.94	33.22
C17	59.55	59.91	59.70	59.86	59.90	59.92	59.96	59.97	59.90	63.53	63.59	63.43	63.58
C18	12.83	12.86	12.89	12.84	12.06	12.13	12.14	12.14	12.06	13.25	12.83	12.95	13.05
C19	12.82	13.29	12.33	12.89	11.57	11.85	11.60	11.61	11.57	12.30	12.68	12.08	12.02
C20	41.51	41.12	41.17	41.19	41.19	41.25	41.10	41.16	41.19	42.51	42.59	42.54	42.67
C21	20.41	19.86	20.30	20.01	19.10	19.00	18.99	18.96	19.10	19.77	19.64	20.00	20.05
C22	38.88	39.23	38.97	39.16	38.51	38.49	38.39	38.34	38.51	40.56	40.28	40.23	40.08
C23	37.44	36.46	37.04	36.71	36.24	36.11	36.16	36.25	36.24	37.03	37.17	37.15	37.24
C24	48.63	49.28	49.24	48.93	49.97	50.12	49.94	49.79	49.97	51.81	52.02	51.95	51.69
C25	32.10	32.09	32.13	32.36	31.97	31.97	31.83	31.92	31.97	31.63	31.40	31.60	31.72
C26	15.90	16.07	15.94	16.00	15.65	15.72	15.60	15.66	15.65	15.95	16.64	16.11	16.54
C27	25.53	25.67	25.69	25.70	25.56	25.61	25.57	25.55	25.56	25.97	26.52	26.29	26.42
C28	32.57	32.63	32.55	32.67	32.64	32.81	32.67	32.74	32.64	33.11	33.37	32.89	32.98
C29	14.77	14.88	14.84	14.88	14.52	14.62	14.54	14.55	14.52	14.17	14.21	14.47	14.45
C5'	105.37	105.58	105.39	105.62	107.24	107.67	107.32	107.80	107.24	109.87	110.57	110.10	110.37
C1''	128.74	128.80	128.74	128.75	131.14	131.20	131.15	131.21	131.14	129.84	129.91	129.72	129.93
C1'''	18.20	18.17	18.29	18.27	18.37	18.37	18.42	18.38	18.37	19.35	19.64	19.26	19.93

The calculated ^{13}C NMR chemical shifts were referenced to TMS using the absolute shielding constants σ(TMS) calculated at the same level of theory for each protocol: Protocol 7 — 187.61 ppm; Protocol 8 — 187.47 ppm; Protocol 8A — 188.68

Table S5. DFT-calculated ^{13}C NMR chemical shifts (δ, ppm) for the four diastereomers (α-*anti*, β-*syn*, α-*syn*, and β-*anti*) of compound **4** obtained at different computational protocols and used for PLS2 model construction

	Protocol 1				Protocol 2					Protocol 2A				Protocol 4			
№	**β-*syn***	**β-*anti***	**α-*syn***	**α-*anti***	**β-*syn***	**β-*anti***	**α-*syn***	**α-*anti***	**β-*syn***	**β-*syn***	**β-*anti***	**α-*syn***	**α-*anti***	**β-*syn***	**β-*anti***	**α-*syn***	**α-*anti***
C1	38.24	39.51	38.30	38.53	39.50	40.30	39.63	40.01	39.50	36.15	36.84	36.47	36.54	40.16	41.22	40.29	40.64
C2	33.07	28.66	32.64	29.55	34.74	30.79	34.26	32.25	34.74	31.32	28.08	30.64	29.11	35.12	31.43	34.31	32.51
C3(3')	126.95	126.86	126.80	126.69	119.89	119.83	120.10	119.87	119.89	114.20	114.11	114.17	114.14	119.96	119.95	119.71	119.71
C4	34.75	39.49	35.80	39.11	36.86	41.12	38.36	40.37	36.86	34.48	37.77	35.50	37.25	37.61	41.54	38.85	40.83
C5	47.89	46.19	46.93	46.30	49.02	48.18	48.76	48.19	49.02	44.58	43.49	44.18	43.89	49.76	48.34	49.21	48.65
C6	30.69	30.33	30.54	30.29	32.75	32.42	32.67	32.39	32.75	28.85	28.52	28.82	28.52	33.26	32.94	33.15	32.82
C7	34.07	33.97	33.73	33.70	36.33	36.15	36.01	36.05	36.33	32.39	32.40	32.23	32.23	36.83	36.83	36.55	36.56
C8	39.00	38.85	38.88	38.90	40.84	40.97	40.59	40.84	40.84	36.36	36.30	36.28	36.27	41.19	40.87	41.21	41.18
C9	56.57	56.42	56.20	56.16	59.80	59.61	59.50	59.17	59.80	54.55	54.56	54.28	54.35	60.14	59.95	59.82	59.73
C10	41.72	41.72	41.41	41.35	42.19	42.37	41.45	42.34	42.19	37.81	37.72	37.53	37.44	42.69	42.75	42.44	42.26
C11	24.35	24.37	24.42	24.26	25.33	25.24	25.26	25.04	25.33	22.68	22.65	22.60	22.59	25.83	25.99	26.11	25.95
C12	41.71	41.81	41.93	41.79	44.51	44.38	44.35	44.43	44.51	40.37	40.39	40.29	40.33	44.87	45.02	45.21	45.14
C13	48.94	49.11	48.78	48.80	49.58	49.52	49.49	49.34	49.58	44.53	44.54	44.50	44.48	50.14	50.56	50.08	50.05
C14	59.05	59.07	59.01	59.11	61.87	61.94	61.82	61.65	61.87	57.58	57.63	57.48	57.49	61.92	61.88	61.91	62.27
C15	27.78	27.90	27.79	27.88	27.93	28.10	28.08	28.04	27.93	25.44	25.41	25.42	25.41	28.78	28.96	28.80	28.84
C16	33.03	33.34	33.13	33.09	33.27	32.91	33.08	32.98	33.27	30.39	30.44	30.34	30.38	33.65	34.13	33.75	33.65
C17	59.83	60.10	60.10	59.88	62.50	62.50	62.46	62.54	62.50	57.86	57.95	57.82	57.85	62.45	62.74	62.57	62.80
C18	14.17	14.30	14.06	13.98	12.42	12.37	12.24	12.29	12.42	13.83	13.86	13.89	13.84	12.98	13.13	12.93	12.90
C19	13.62	13.62	13.32	13.34	11.92	11.82	11.74	11.59	11.92	13.21	13.20	13.07	13.11	13.02	13.04	12.61	12.63
C20	41.01	41.09	41.00	40.85	43.61	43.67	43.60	43.51	43.61	38.97	38.97	38.94	39.01	43.26	43.66	43.60	43.05
C21	19.53	19.34	19.26	19.22	19.23	19.23	19.06	19.04	19.23	18.99	18.98	18.89	18.90	20.00	19.78	19.73	19.45
C22	37.81	37.71	38.01	37.62	39.93	40.05	40.03	39.99	39.93	36.59	36.55	36.55	36.53	40.78	40.63	40.97	40.50
C23	36.22	35.95	35.72	36.34	37.82	37.91	37.81	37.79	37.82	34.76	34.72	34.67	34.66	38.64	38.34	38.43	38.37
C24	48.70	48.77	48.17	47.99	52.23	52.07	52.12	52.26	52.23	46.29	46.14	46.31	46.15	52.54	52.58	51.78	51.46
C25	31.91	31.26	31.65	31.33	33.60	33.26	33.57	33.82	33.60	28.65	28.66	28.62	28.56	34.00	33.46	33.51	33.61
C26	16.54	16.39	16.52	16.40	15.54	15.63	15.54	15.63	15.54	15.97	15.89	15.92	15.95	16.50	16.27	16.43	16.40
C27	25.25	25.29	25.47	25.21	25.67	25.70	25.70	25.67	25.67	24.76	24.74	24.73	24.71	26.12	26.30	26.41	26.27
C28	32.29	32.13	32.33	32.43	34.03	33.99	33.96	33.98	34.03	30.84	30.94	30.85	30.86	34.38	34.16	34.50	34.56
C29	14.68	14.67	14.69	14.75	14.59	14.54	14.58	14.64	14.59	14.28	14.28	14.23	14.28	15.14	15.09	15.05	15.34
C5'	112.20	112.40	112.05	111.85	110.99	111.19	110.67	110.73	110.99	106.50	106.61	106.34	106.27	110.11	110.34	109.80	109.82
C1''	142.50	142.48	142.68	142.60	133.58	133.56	133.55	133.62	133.58	129.62	129.55	129.68	129.74	132.20	132.26	132.20	132.19
C_q	127.79	127.68	127.41	127.35	139.92	139.96	139.71	139.55	139.92	133.01	133.02	132.86	132.77	140.86	140.93	140.36	140.63
C_{ortho}	123.93	123.94	123.99	123.93	133.35	133.34	133.26	133.29	133.35	128.27	128.28	128.27	128.27	133.71	133.66	133.63	133.54
C_{meta}	123.51	123.48	123.43	123.54	132.81	132.82	132.85	132.78	132.81	128.30	128.31	128.26	128.26	133.13	133.10	133.16	133.13
C_{para}	126.01	125.99	126.04	125.93	135.19	135.26	135.26	135.28	135.19	131.08	131.08	131.10	131.09	135.50	135.50	135.52	135.47

The calculated ^{13}C NMR chemical shifts were referenced to TMS using the absolute shielding constants σ(TMS) calculated at the same level of theory for each protocol: Protocol 1 — 194.37 ppm; Protocol 2 — 182.48 ppm; Protocol 2A — 195.12 ppm; Protocol 4 — 184.41 ppm

Table S5. DFT-calculated ^{13}C NMR chemical shifts (δ, ppm) for the four diastereomers (α-*anti*, β-*syn*, α-*syn*, and β-*anti*) of compound **4** obtained at different computational protocols and used for PLS2 model construction

	Protocol 4A				Protocol 5					Protocol 7				Protocol 8			
№	**β-*syn***	**β-*anti***	**α-*syn***	**α-*anti***	**β-*syn***	**β-*anti***	**α-*syn***	**α-*anti***	**β-*syn***	**β-*syn***	**β-*anti***	**α-*syn***	**α-*anti***	**β-*syn***	**β-*anti***	**α-*syn***	**α-*anti***
C1	37.80	38.73	38.02	38.41	37.91	38.76	38.02	38.50	37.91	38.29	38.80	38.29	38.53	38.41	38.83	38.31	38.62
C2	33.01	29.34	32.26	30.48	32.93	29.34	32.18	30.47	32.93	31.99	29.02	31.79	30.07	31.84	28.89	31.47	29.80
C3(3')	117.98	117.90	118.01	117.90	117.79	117.74	117.85	117.79	117.79	115.69	115.61	115.59	115.49	116.68	116.59	116.47	116.45
C4	35.43	39.25	36.65	38.59	35.46	39.21	36.64	38.55	35.46	35.23	38.48	36.54	38.18	35.05	38.38	36.30	37.89
C5	47.02	45.69	46.64	45.98	46.88	45.61	46.54	45.85	46.88	46.67	45.59	46.23	45.60	46.63	45.56	46.18	45.63
C6	31.18	30.79	31.15	30.80	31.24	30.85	31.21	30.86	31.24	31.56	31.09	31.61	31.14	31.54	31.14	31.64	31.15
C7	34.54	34.59	34.33	34.36	34.51	34.61	34.36	34.37	34.51	34.96	35.20	34.82	34.83	34.79	35.09	34.66	34.72
C8	38.92	38.83	38.86	38.87	38.76	38.67	38.70	38.72	38.76	38.66	38.47	38.62	38.62	38.55	38.42	38.57	38.52
C9	57.02	56.94	56.54	56.65	56.99	56.90	56.49	56.62	56.99	56.98	56.56	56.16	56.19	56.87	56.45	55.98	56.16
C10	40.58	40.47	40.23	40.09	40.34	40.22	40.03	39.87	40.34	38.96	38.94	38.61	38.51	38.77	38.79	38.49	38.36
C11	24.08	24.03	24.04	23.94	24.07	24.01	24.02	23.92	24.07	24.22	24.12	24.07	23.92	24.20	24.07	24.05	23.88
C12	42.42	42.45	42.41	42.46	42.50	42.51	42.49	42.54	42.50	42.66	42.50	42.56	42.47	42.63	42.47	42.51	42.46
C13	47.63	47.65	47.60	47.60	47.44	47.45	47.42	47.41	47.44	45.75	45.70	45.76	45.79	45.74	45.62	45.72	45.75
C14	59.20	59.32	59.07	59.11	59.16	59.27	58.99	59.04	59.16	59.40	59.38	58.97	59.01	59.21	59.19	58.74	58.84
C15	26.92	26.91	26.88	26.84	26.91	26.91	26.87	26.83	26.91	27.32	27.38	27.33	27.36	27.20	27.30	27.25	27.28
C16	31.64	31.75	31.64	31.60	31.65	31.76	31.63	31.60	31.65	32.31	32.40	32.20	32.15	32.28	32.28	32.07	32.06
C17	59.81	59.92	59.86	60.03	59.80	59.90	59.89	60.02	59.80	59.60	59.58	59.83	59.55	59.40	59.40	59.64	59.41
C18	11.99	12.07	12.04	12.03	12.07	12.16	12.12	12.11	12.07	12.77	12.84	12.80	12.83	12.75	12.89	12.85	12.79
C19	11.77	11.69	11.48	11.51	11.86	11.77	11.56	11.59	11.86	12.93	12.60	12.61	12.44	13.06	12.63	12.71	12.53
C20	41.36	41.31	41.35	41.26	41.30	41.24	41.26	41.19	41.30	41.42	41.39	41.17	41.21	41.36	41.32	41.04	41.12
C21	19.07	18.85	18.81	18.55	19.18	18.96	18.85	18.64	19.18	20.48	20.34	19.86	20.20	20.39	20.34	19.82	19.98
C22	38.23	38.45	38.25	38.21	38.30	38.50	38.30	38.26	38.30	39.23	39.14	38.85	38.75	39.20	38.99	38.64	38.66
C23	36.38	36.06	36.23	36.05	36.46	36.14	36.26	36.13	36.46	37.15	37.11	36.83	37.12	37.05	37.15	36.82	36.85
C24	49.93	49.93	49.85	49.57	49.91	49.90	49.81	49.51	49.91	49.05	48.88	48.74	48.80	49.02	48.93	48.77	48.84
C25	32.05	32.05	32.04	32.10	31.97	31.96	31.97	32.02	31.97	32.12	32.15	31.89	31.88	31.99	31.97	31.71	31.60
C26	15.59	15.54	15.52	15.58	15.70	15.65	15.67	15.70	15.70	15.96	16.02	16.03	15.97	15.94	15.97	16.05	15.86
C27	25.38	25.47	25.43	25.52	25.53	25.63	25.63	25.67	25.53	25.57	25.63	25.54	25.56	25.61	25.62	25.60	25.62
C28	32.64	32.60	32.66	32.72	32.73	32.69	32.81	32.82	32.73	32.52	32.65	32.68	32.63	32.41	32.55	32.66	32.47
C29	14.46	14.47	14.50	14.43	14.58	14.58	14.63	14.55	14.58	14.82	14.80	14.76	14.76	14.85	14.80	14.70	14.71
C5'	108.23	108.41	107.99	107.94	108.28	108.47	107.89	107.85	108.28	106.08	106.21	105.94	105.95	105.90	106.02	105.59	105.62
C1''	131.02	131.05	131.01	131.00	131.01	131.05	130.99	131.00	131.01	128.57	128.60	128.57	128.56	128.82	128.84	128.78	128.79
C_q	138.30	138.21	138.08	138.13	138.26	138.14	138.12	138.13	138.26	138.12	138.04	137.83	138.21	137.91	137.77	137.71	138.18
C_{ortho}	132.78	132.77	132.79	132.79	132.63	132.62	132.65	132.64	132.63	132.62	132.62	132.63	132.59	132.65	132.66	132.68	132.64
C_{meta}	132.30	132.31	132.28	132.28	132.20	132.22	132.18	132.18	132.20	132.36	132.37	132.29	132.31	132.51	132.53	132.44	132.46
C_{para}	134.70	134.73	134.68	134.69	134.55	134.59	134.52	134.53	134.55	134.66	134.67	134.59	134.61	134.67	134.69	134.60	134.62

The calculated ^{13}C NMR chemical shifts were referenced to TMS using the absolute shielding constants σ(TMS) calculated at the same level of theory for each protocol: Protocol 4A — 187.16 ppm; Protocol 5 — 187.49 ppm; Protocol 7 — 187.61 ppm; Protocol 8 — 187.47 ppm

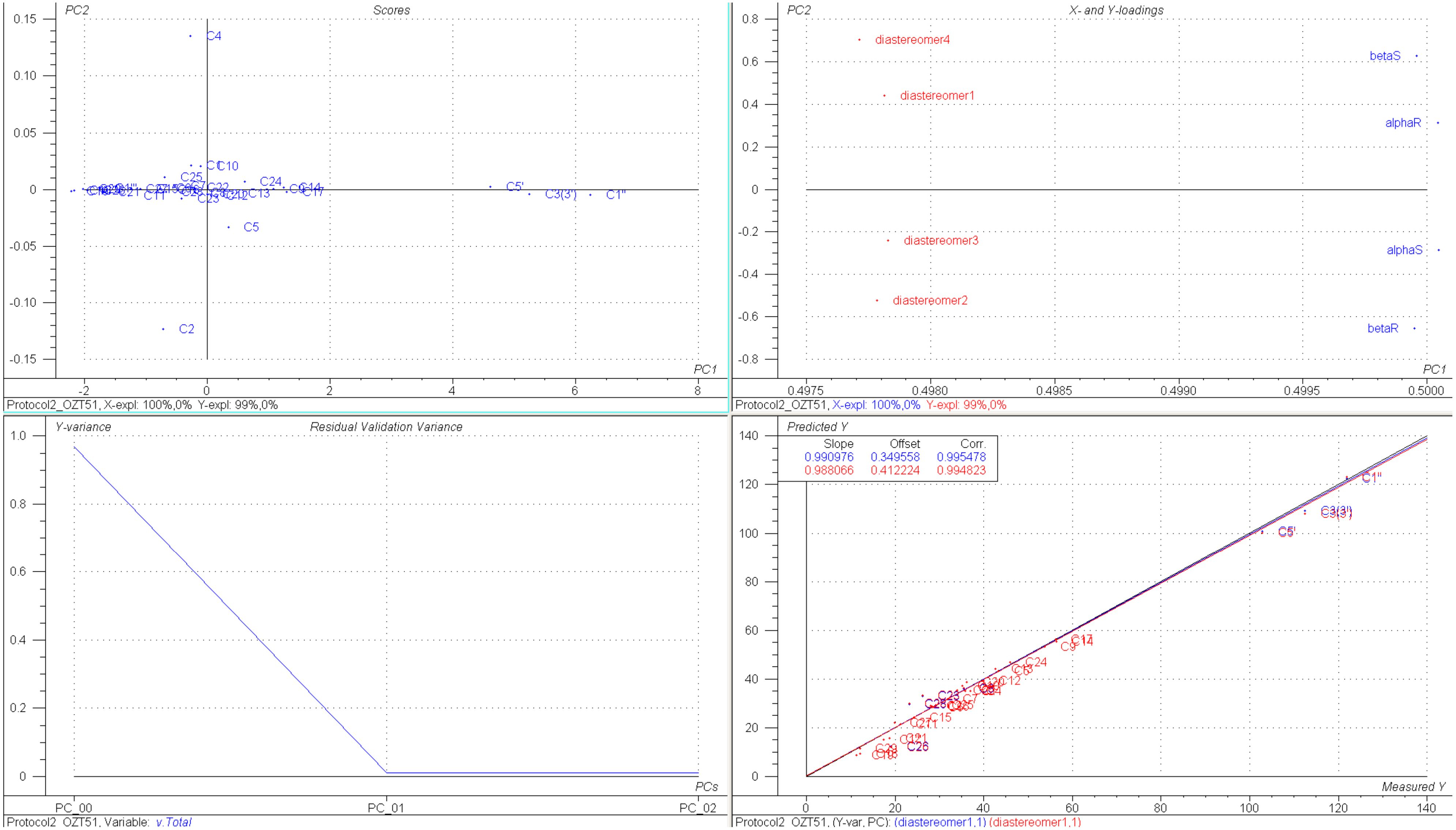

Figure S2. PLS2 analysis of compound **3** (protocol 2) prior to the removal of structural outliers. In the Scores plot (PC-01 vs PC-02), atoms C3(3'), C5', and C1" show significant deviation along PC-01 (values > 4.5), allowing them to be identified as structural outliers.

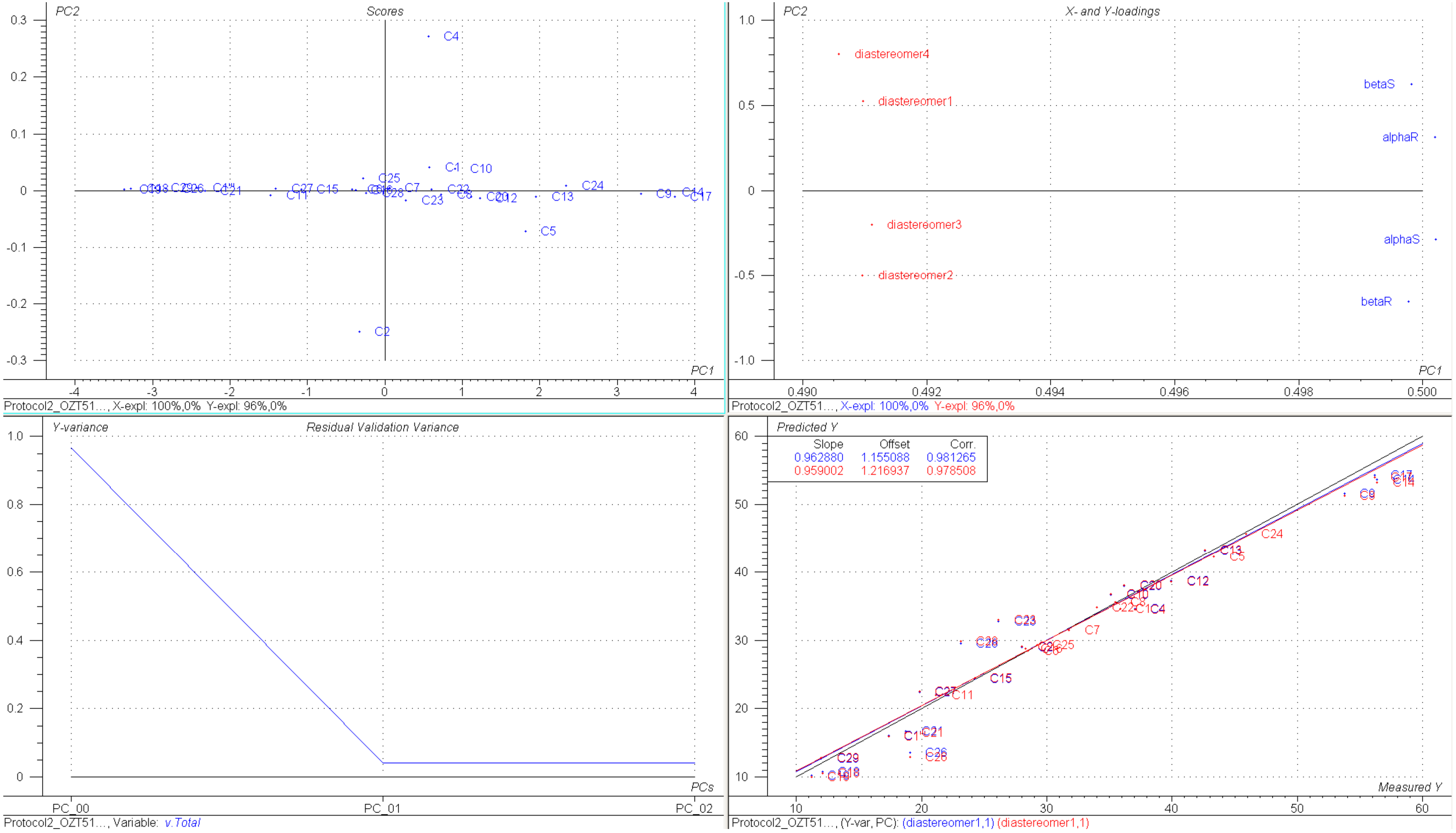

Figure S3. PLS2 analysis of compound **3** (protocol 2) after removal of structural outliers (C3(3'), C5', and C1"). The exclusion of atoms with PC-01 > 4.5 led to a significant improvement in the model quality: the explained variance for PC-01 and PC-02 increased from 1.02% and 0.99% to 4.14% and 4.03%, respectively.

**Table S6**. Statistical parameters of refined PLS2 models for different computational protocols based on the diastereomeric mixture of compound **3**.							
№		**PC-02, %**	**R^2**	**Bias**	**RMSEC**	**Slope**	**Intercept**
1	B3LYP/6-31G // B3LYP/6-31G GIAO	1.17	0.950	+0.346	2.951	0.813	4.294
2	B3LYP/6-31G(d) // B3LYP/6-311+G(2d,p) GIAO	4.03	0.954	+0.548	2.812	0.857	2.830
2A	B3LYP/6-31G(d) // wB97XD/Def2SVP PCM($CHCl_3$) GIAO	3.43	0.970	+0.241	2.266	0.852	2.705
3	B3LYP/6-31G(d) SMD (CH_2Cl_2) // B3LYP/6-311+G(2d,p) GIAO	4.09	0.949	+0.620	2.953	0.856	2.863
4	B3LYP/6-311G(d,p) // B3LYP/6-311G(d,p) GIAO	3.94	0.955	+0.509	2.796	0.858	2.843
4A	B3LYP/6-311G(d,p) // MPW1PW91/6-311+G(2d,p) CSGT	3.90	0.953	+0.534	2.850	0.855	2.915
5	B3LYP/6-311+G(2d,p) // MPW1PW91/6-311+G(2d,p) CSGT	3.92	0.959	+0.388	2.661	0.858	2.802
6	B3LYP/6-311+G(2d,p) SMD (CH_2Cl_2) // MPW1PW91/6-311+G(2d,p) CSGT	4.06	0.966	+0.022	2.407	0.785	5.390
7	M062X/6-311+G(d,p) // MPW1PW91/6-311+G(2d,p) CSGT	4.15	0.944	+0.632	3.116	0.904	1.746
8	M062X/6-311+G(d,p) SMD ($CHCl_3$) // MPW1PW91/6-311+G(2d,p) CSGT	3.92	0.959	+0.388	2.661	0.858	2.802
8A	M062X/6-311+G(d,p) SMD ($CHCl_3$) // M062X/6-311+G(d,p) GIAO	3.19	0.939	+0.115	3.251	0.798	4.840

PC-02, % – indicates the proportion of variance in the data responsible for the separation of diastereomers (component informativeness).
R^2 – coefficient of determination. Indicates what fraction of the variance in the experimental data the model explains overall (overall predictive power).
RMSEC – root-mean-square error of calibration. Indicates the absolute accuracy of predictions in units of chemical shift (ppm).
Bias – systematic error. Indicates whether the model has a tendency to overestimate or underestimate predictions.
Slope and **Intercept** – parameters of the regression line: Predicted = Slope × Experimental + Intercept. They characterize how ideally (Slope = 1, Intercept = 0) the model reproduces the experiment**.**

**Table S7**. Refined PLS2-model PC-02 loadings for different computational protocols based on the diastereomeric mixture of compound **3**									
	**Approximations**	***α-anti***		***β-syn***		***α-syn***		***β-anti***	
		**X**	**Y (→ d_1)**	**X**	**Y (→ d_2)**	**X**	**Y (→ d_3)**	**X**	**Y (→ d_4)**
1	B3LYP/6-31G // B3LYP/6-31G GIAO	+0.388	+0.387	–0.495	–0.477	–0.232	–0.233	+0.614	+0.551
2	B3LYP/6-31G(d) // B3LYP/6-311+G(2d,p) GIAO	+0.312	+0.442	–0.655	–0.522	–0.291	–0.242	+0.627	+0.703
2A	B3LYP/6-31G(d) // wB97XD/Def2SVP PCM($CHCl_3$) GIAO	**–0.340**	**–0.393**	**+0.636**	**+0.735**	**+0.319**	**+0.400**	**–0.615**	**–0.685**
3	B3LYP/6-31G(d) SMD (CH_2Cl_2) // B3LYP/6-311+G(2d,p) GIAO	+0.321	+0.444	–0.654	–0.542	–0.289	–0.255	+0.622	+0.707
4	B3LYP/6-311G(d,p) // B3LYP/6-311G(d,p) GIAO	+0.300	+0.458	–0.649	–0.552	–0.289	–0.251	+0.639	+0.717
4A	B3LYP/6-311G(d,p) // MPW1PW91/6-311+G(2d,p) CSGT	+0.316	+0.417	–0.646	–0.606	–0.299	–0.304	+0.628	+0.680
5	B3LYP/6-311+G(2d,p) // MPW1PW91/6-311+G(2d,p) CSGT	+0.310	+0.412	–0.647	–0.614	–0.294	–0.311	+0.631	+0.675
6	B3LYP/6-311+G(2d,p) SMD (CH_2Cl_2) // MPW1PW91/6-311+G(2d,p) CSGT	+0.300	+0.441	–0.647	–0.584	–0.291	–0.279	+0.638	+0.705
7	M062X/6-311+G(d,p) // MPW1PW91/6-311+G(2d,p) CSGT	+0.335	+0.240	–0.656	–0.799	–0.293	–0.492	+0.615	+0.504
8	M062X/6-311+G(d,p) SMD ($CHCl_3$) // MPW1PW91/6-311+G(2d,p) CSGT	+0.300	+0.436	–0.647	–0.609	–0.291	–0.300	+0.639	+0.706
8A	M062X/6-311+G(d,p) SMD ($CHCl_3$) // M062X/6-311+G(d,p) GIAO	**–0.352**	**–0.450**	**+0.673**	**+0.614**	**+0.271**	**+0.304**	**–0.592**	**–0.720**

Values in **bold** indicate inverted sign of PC-02. This is a computational artifact due to differences in functional, basis set, NMR method, and solvation model; it does not affect the stereochemical assignment, which remains consistent: *α*-anti → d_1, *β*-syn → d_2, *α*-syn → d_3, and *β*-anti → d_4

**Table S8**. Summary of PLS2-based stereochemical assignment for compound **3** and compound **4** across different computational protocols					
	**Approximations**	***α-anti***	***β-syn***	***α-syn***	***β-anti***
№		compound **3**			
1	B3LYP/6-31G // B3LYP/6-31G GIAO	d_1	d_2	d_3	d_4
2	B3LYP/6-31G(d) // B3LYP/6-311+G(2d,p) GIAO	d_1	d_2	d_3	d_4
2A	B3LYP/6-31G(d) // wB97XD/Def2SVP PCM($CHCl_3$) GIAO	d_1	d_2	d_3	d_4
3	B3LYP/6-31G(d) SMD (CH_2Cl_2) // B3LYP/6-311+G(2d,p) GIAO	d_1	d_2	d_3	d_4
4	B3LYP/6-311G(d,p) // B3LYP/6-311G(d,p) GIAO	d_1	d_2	d_3	d_4
4A	B3LYP/6-311G(d,p) // MPW1PW91/6-311+G(2d,p) CSGT	d_1	d_2	d_3	d_4
5	B3LYP/6-311+G(2d,p) // MPW1PW91/6-311+G(2d,p) CSGT	d_1	d_2	d_3	d_4
6	B3LYP/6-311+G(2d,p) SMD (CH_2Cl_2) // MPW1PW91/6-311+G(2d,p) CSGT	d_1	d_2	d_3	d_4
7	M062X/6-311+G(d,p) // MPW1PW91/6-311+G(2d,p) CSGT	d_1	d_2	d_3	d_4
8	M062X/6-311+G(d,p) SMD ($CHCl_3$) // MPW1PW91/6-311+G(2d,p) CSGT	d_1	d_2	d_3	d_4
8A	M062X/6-311+G(d,p) SMD ($CHCl_3$) // M062X/6-311+G(d,p) GIAO	d_1	d_2	d_3	d_4
		compound **4**			
1	B3LYP/6-31G // B3LYP/6-31G GIAO	d_4	d_1	d_3	d_2
2	B3LYP/6-31G(d) // B3LYP/6-311+G(2d,p) GIAO	d_4	d_1	d_3	d_2
2A	B3LYP/6-31G(d) // wB97XD/Def2SVP PCM($CHCl_3$) GIAO	d_4	d_1	d_3	d_2
4	B3LYP/6-311G(d,p) // B3LYP/6-311G(d,p) GIAO	d_4	d_1	d_3	d_2
4A	B3LYP/6-311G(d,p) // MPW1PW91/6-311+G(2d,p) CSGT	d_4	d_1	d_3	d_2
5	B3LYP/6-311+G(2d,p) // MPW1PW91/6-311+G(2d,p) CSGT	d_4	d_1	d_3	d_2
7	M062X/6-311+G(d,p) // MPW1PW91/6-311+G(2d,p) CSGT	d_4	d_1	d_3	d_2
8	M062X/6-311+G(d,p) SMD ($CHCl_3$) // MPW1PW91/6-311+G(2d,p) CSGT	d_4	d_1	d_3	d_2

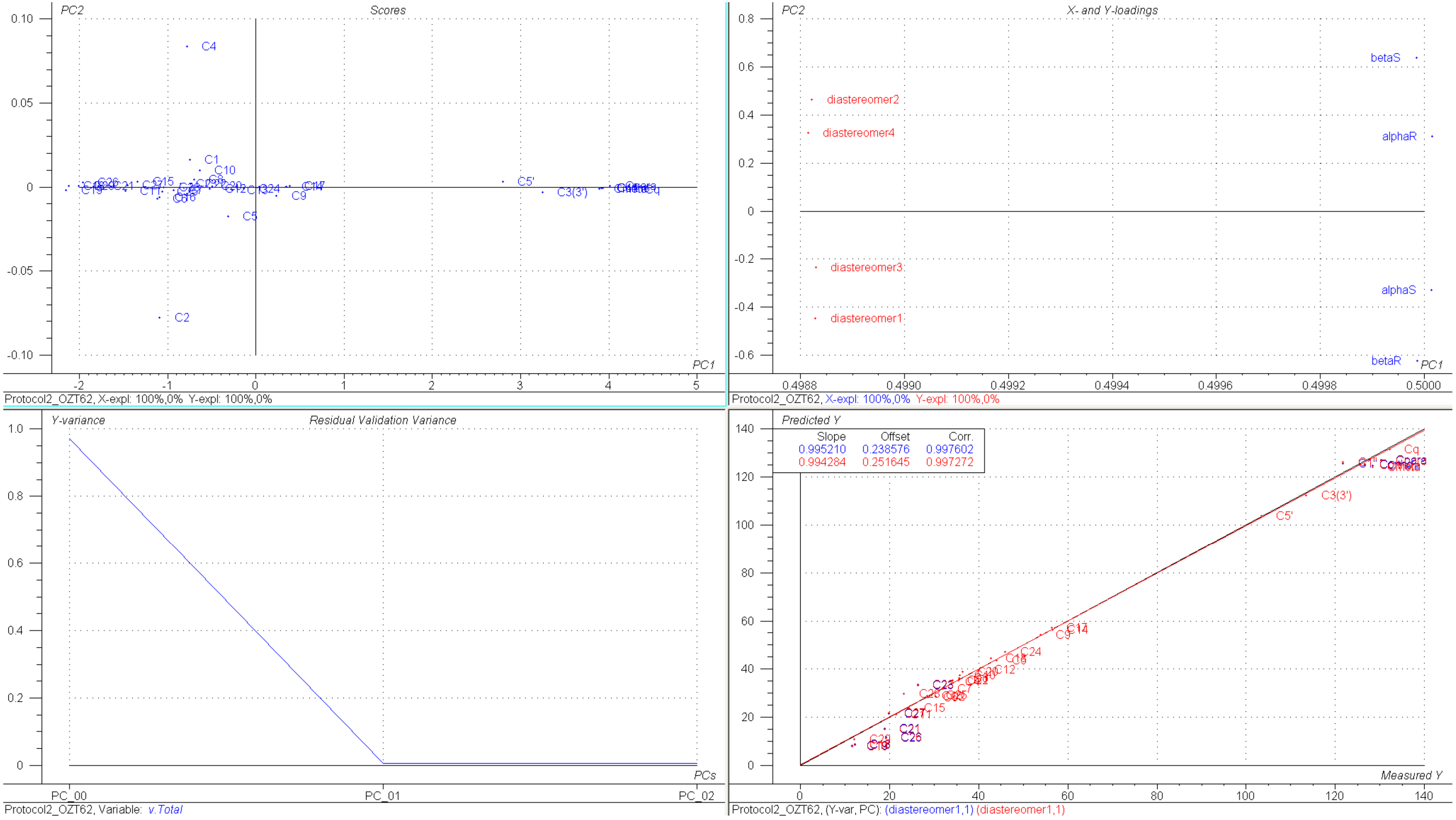

Figure S4. PLS2 analysis of compound **4** (protocol 2) prior to outlier removal. Aromatic carbons and C1'' exceed the Tukey threshold (3.68), while C3(3') and C5' remain below it.

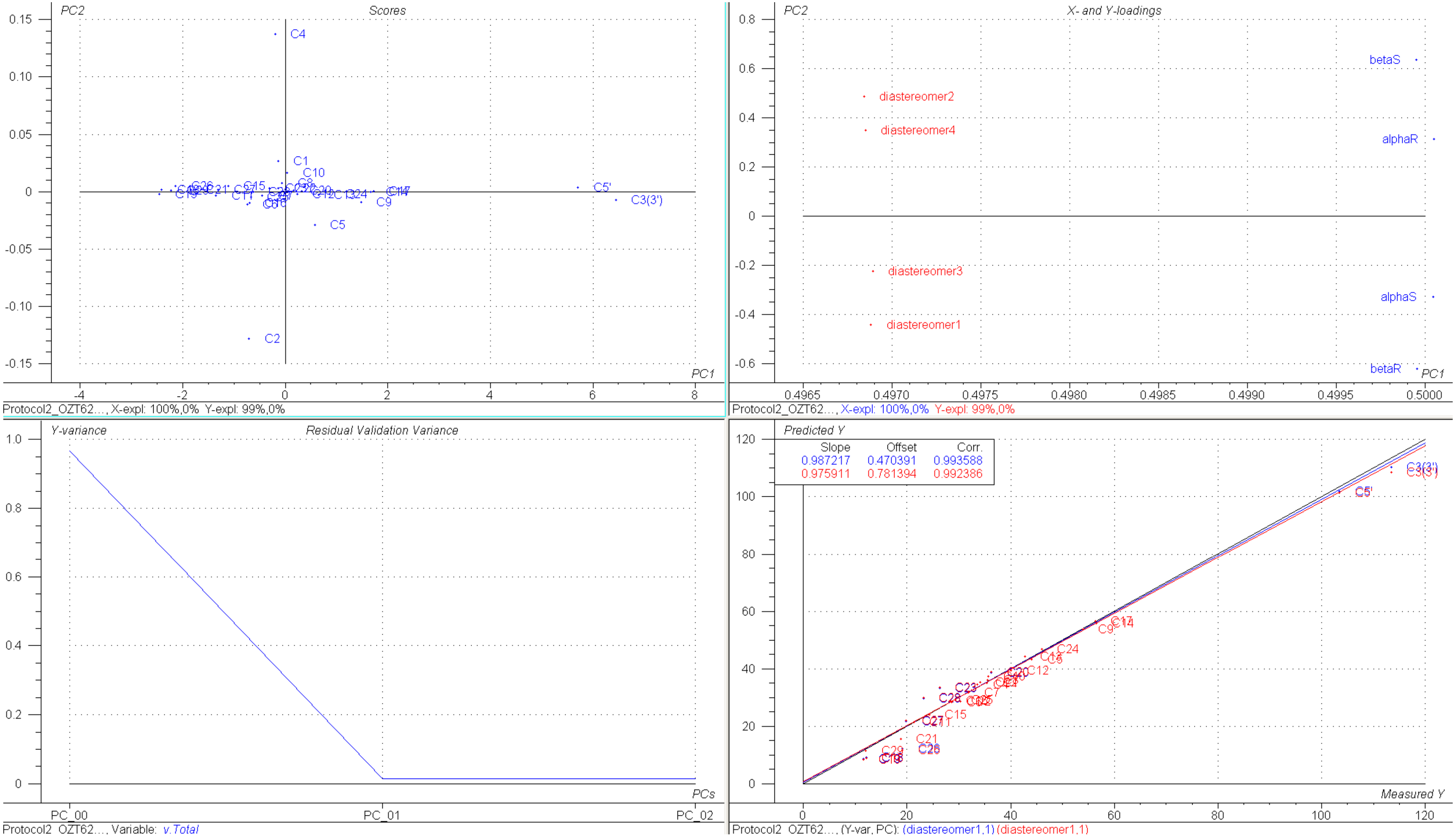

Figure S5. PLS2 analysis of compound **4** (protocol 2) after removal of structural outliers (aromatic carbons and C1"). The exclusion significantly improved the model: PC-01 and PC-02 increased from 0.53% to 4.21% and 4.22%, respectively.

Table S9. Identification of structural outliers for compound **4** across different computational protocols using the Tukey criterion (Q3 + 1.5 × IQR)

Protocol	Tukey threshold	**C3(3')**	**C5'**	**C1"**	**C_q**	**C_{ortho}**	**C_{meta}**	**C_{para}**
1	0.93	**3.78**	**3.03**	**4.59**	**3.82**	**3.64**	**3.61**	**3.74**
2	3.68	3.25	2.80	**3.93**	**4.24**	**3.92**	**3.89**	**4.01**
2A	3.23	**2.83**	**4.03**	**4.19**	**3.95**	**3.96**	**4.10**	**2.15**
4	3.65	3.24	2.75	**3.87**	**4.29**	**3.94**	**3.91**	**4.03**
4A	3.62	3.24	2.75	**3.89**	**4.25**	**3.98**	**3.96**	**4.08**
5	3.62	3.24	2.75	**3.90**	**4.26**	**3.98**	**3.96**	**4.08**
7	3.64	3.17	2.68	**3.83**	**4.31**	**4.03**	**4.02**	**4.13**
8	3.62	3.21	2.67	**3.83**	**4.29**	**4.03**	**4.02**	**4.13**

Values exceeding the Tukey threshold (Q3 + 1.5 × IQR) are considered structural outliers and are shown in **bold**

Table S10. PLS2 PC-02 loadings for different computational protocols based on the diastereomeric mixture of compound **4**.

	**Approximations**	**β-*syn***		**β-*anti***		**α-*syn***		**α-*anti***	
		**X**	**Y (→ d_1)**	**X**	**Y (→ d_2)**	**X**	**Y (→ d_3)**	**X**	**Y (→ d_4)**
1	B3LYP/6-31G // B3LYP/6-31G GIAO	–0.600	–0.434	+0.576	+0.307	–0.382	–0.257	+0.406	+0.201
2	B3LYP/6-31G(d) // B3LYP/6-311+G(2d,p) GIAO	–0.623	–0.442	+0.638	+0.489	–0.328	–0.224	+0.313	+0.349
2A	B3LYP/6-31G(d) // wB97XD/Def2SVP PCM($CHCl_3$) GIAO	**+0.643**	**+0.699**	**–0.628**	**–0.395**	**+0.302**	**+0.441**	**–0.318**	**–0.237**
4	B3LYP/6-311G(d,p) // B3LYP/6-311G(d,p) GIAO	–0.650	–0.425	+0.630	+0.527	–0.296	–0.201	+0.316	+0.388
4A	B3LYP/6-311G(d,p) // MPW1PW91/6-311+G(2d,p) CSGT	–0.642	–0.418	+0.623	+0.558	–0.308	–0.187	+0.326	+0.418
5	B3LYP/6-311+G(2d,p) // MPW1PW91/6-311+G(2d,p) CSGT	–0.640	–0.434	+0.624	+0.566	–0.310	–0.196	+0.325	+0.423
7	M062X/6-311+G(d,p) // MPW1PW91/6-311+G(2d,p) CSGT	**+0.636**	**+0.748**	**–0.608**	**–0.377**	**+0.325**	**+0.479**	**–0.353**	**–0.216**
8	M062X/6-311+G(d,p) SMD ($CHCl_3$) // MPW1PW91/6-311+G(2d,p) CSGT	**+0.643**	**+0.724**	**–0.613**	**–0.406**	**+0.310**	**+0.451**	**–0.341**	**–0.247**

Values in **bold** indicate inverted sign of PC-02. This is a computational artifact due to differences in functional, basis set, NMR method, and solvation model; it does not affect the stereochemical assignment, which remains consistent: *β*-syn → d_1, *β*-anti → d_2, *α*-syn → d_3, and *α*-anti → d_4.

**Table S11**. Statistical parameters of refined PLS2 models for different computational protocols based on the diastereomeric mixture of compound **4**.							
№		**PC-02, %**	**R^2**	**Bias**	**RMSEC**	**Slope**	**Intercept**
1	B3LYP/6-31G // B3LYP/6-31G GIAO	4.16	0.951	+0.887	3.611	0.842	3.802
2	B3LYP/6-31G(*d*) // B3LYP/6-311+G(*2d,p*) GIAO	4.22	0.960	+0.893	3.301	0.835	3.387
2A	B3LYP/6-31G(*d*) // wB97XD/Def2SVP PCM($CHCl_3$) GIAO	3.64	0.970	+0.034	2.272	0.852	2.687
4	B3LYP/6-311G(*d,p*) // B3LYP/6-311G(*d,p*) GIAO	4.17	0.960	+0.813	3.257	0.814	3.498
4A	B3LYP/6-311G(*d,p*) // MPW1PW91/6-311+G(*2d,p*) CSGT	4.14	0.962	+0.795	3.166	0.818	3.029
5	B3LYP/6-311+G(*2d,p*) // MPW1PW91/6-311+G(*2d,p*) CSGT	4.17	0.964	+0.789	3.095	0.820	2.980
7	M062X/6-311+G(*d,p*) // MPW1PW91/6-311+G(*2d,p*) CSGT	4.46	0.950	+0.452	3.657	0.656	5.727
8	M062X/6-311+G(*d,p*) SMD ($CHCl_3$) // MPW1PW91/6-311+G(*2d,p*) CSGT	4.52	0.953	+0.701	3.530	0.719	4.263
8A	M062X/6-311+G(*d,p*) SMD ($CHCl_3$) // M062X/6-311+G(*d,p*) GIAO	4.16	0.951	+0.887	3.611	0.842	3.802

PC-02, % – indicates the proportion of variance in the data responsible for the separation of diastereomers (component informativeness).
R^2 – coefficient of determination. Indicates what fraction of the variance in the experimental data the model explains overall (overall predictive power).
RMSEC – root-mean-square error of calibration. Indicates the absolute accuracy of predictions in units of chemical shift (ppm).
Bias – systematic error. Indicates whether the model has a tendency to overestimate or underestimate predictions.
Slope and **Intercept** – parameters of the regression line: Predicted = Slope × Experimental + Intercept. They characterize how ideally (Slope = 1, Intercept = 0) the model reproduces the experiment**.**

NMR data

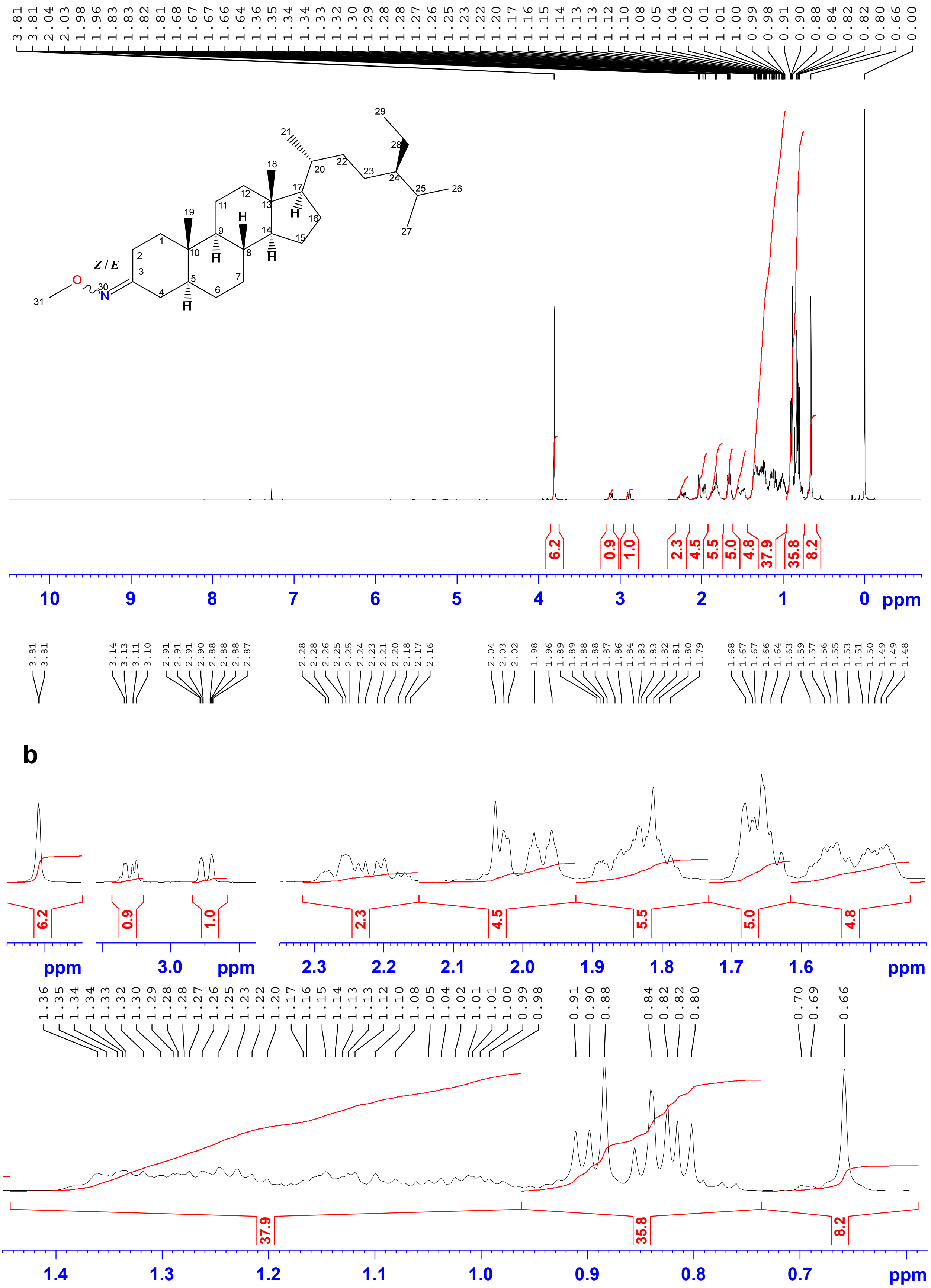

Figure S6. ^{1}H NMR spectrum of the E/Z isomeric mixture of compound **2:** (a) full spectrum; (b) expanded region.

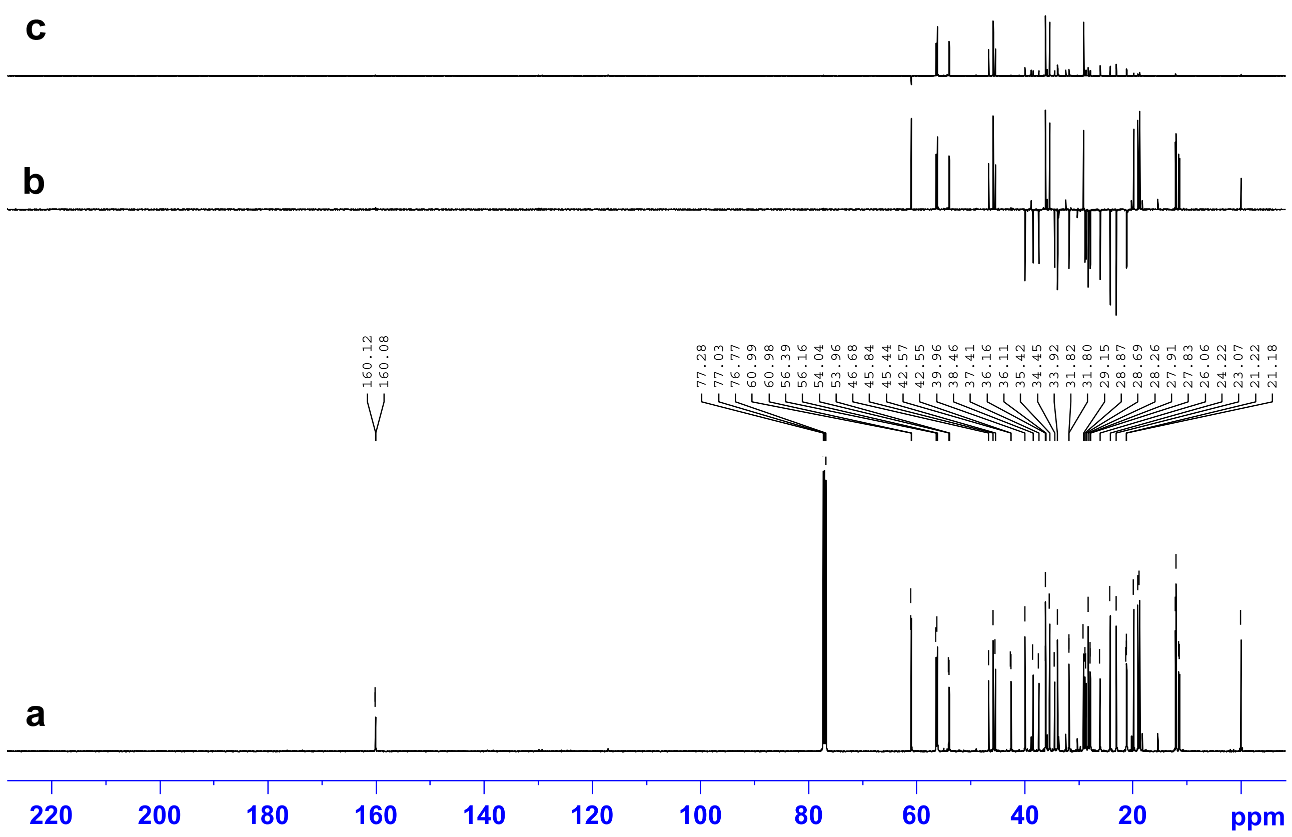

Figure S7. ^{13}C NMR spectra of the E/Z isomeric mixture of compound **2**: (a) $^{13}C\{^1H\}$, (b) DEPT-135, (c) DEPT-90**.**

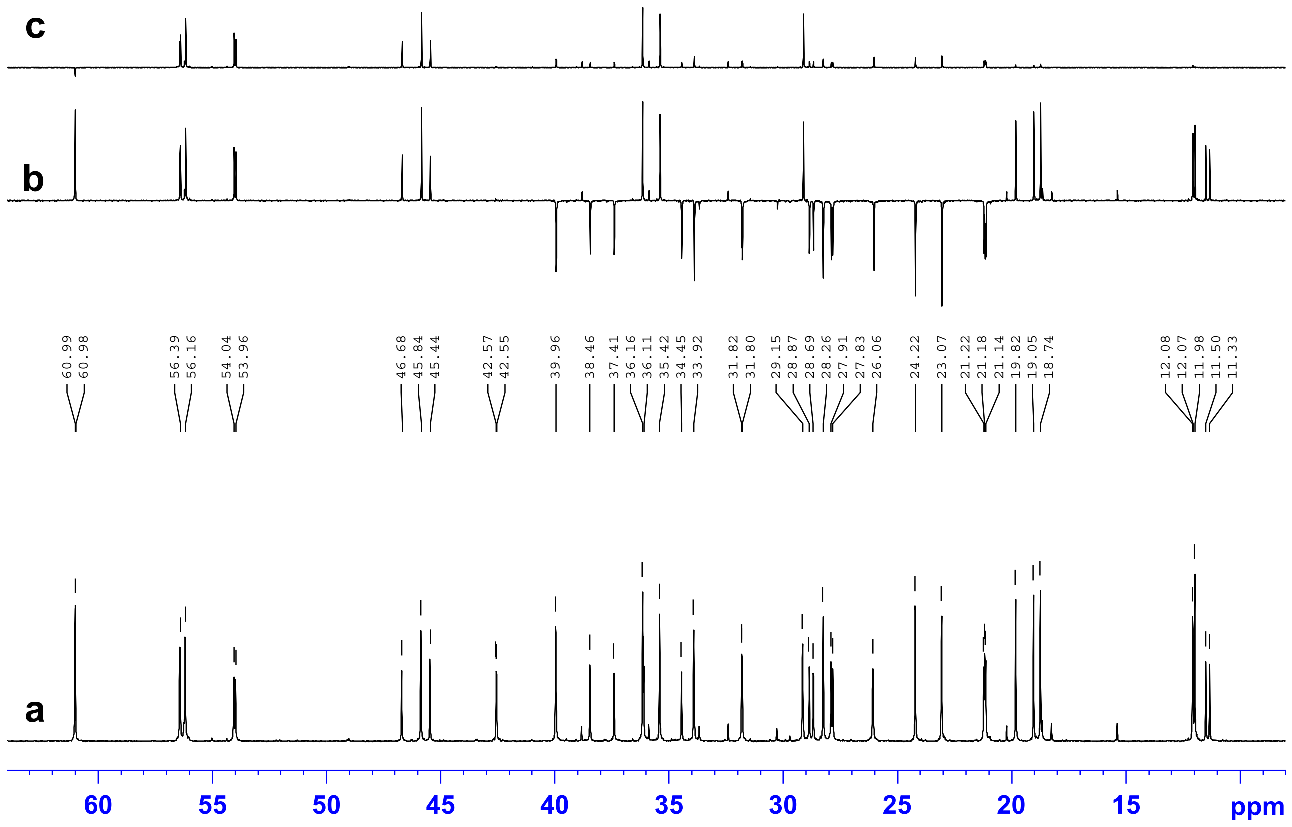

Figure S8. Expanded ^{13}C NMR spectra of the E/Z isomeric mixture of compound **2**: (a) $^{13}C\{^1H\}$, (b) DEPT-135, (c) DEPT-90.

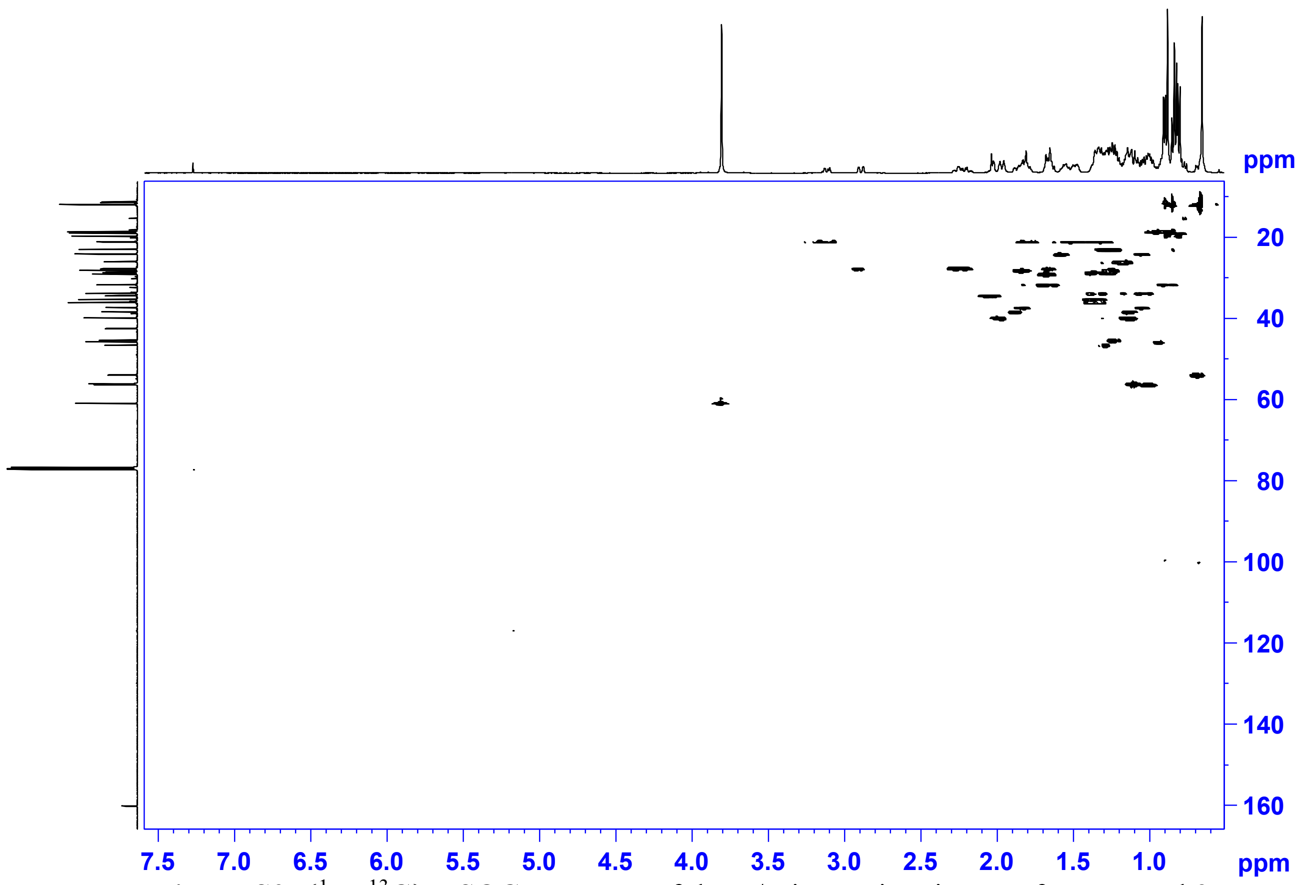

Figure S9. {^{1}H, ^{13}C} HSQC spectrum of the E/Z isomeric mixture of compound **2**.

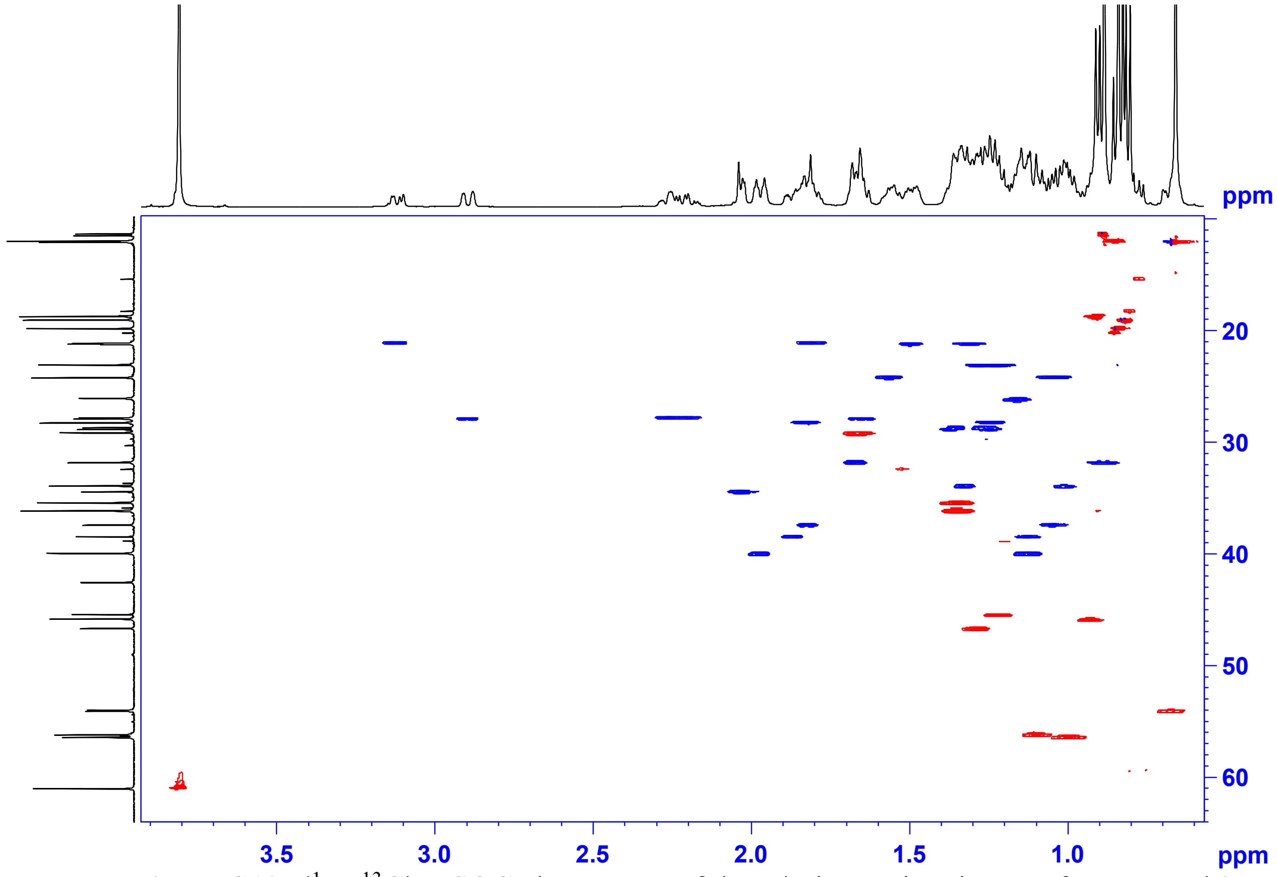

Figure S10. {^{1}H, ^{13}C} HSQCed spectrum of the E/Z isomeric mixture of compound **2**.

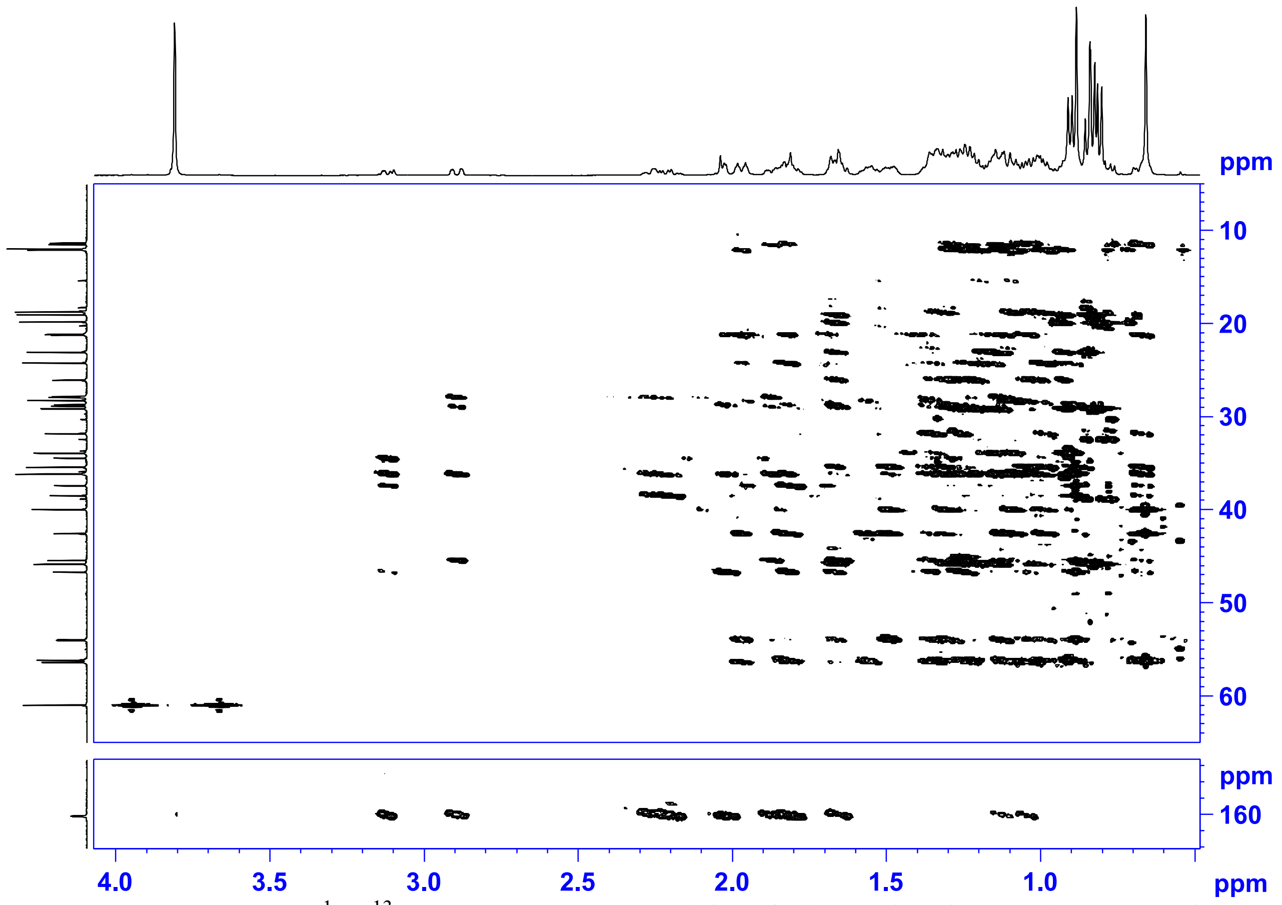

Figure S11. {^{1}H, ^{13}C} HMBC spectrum of the E/Z isomeric mixture of compound **2**.

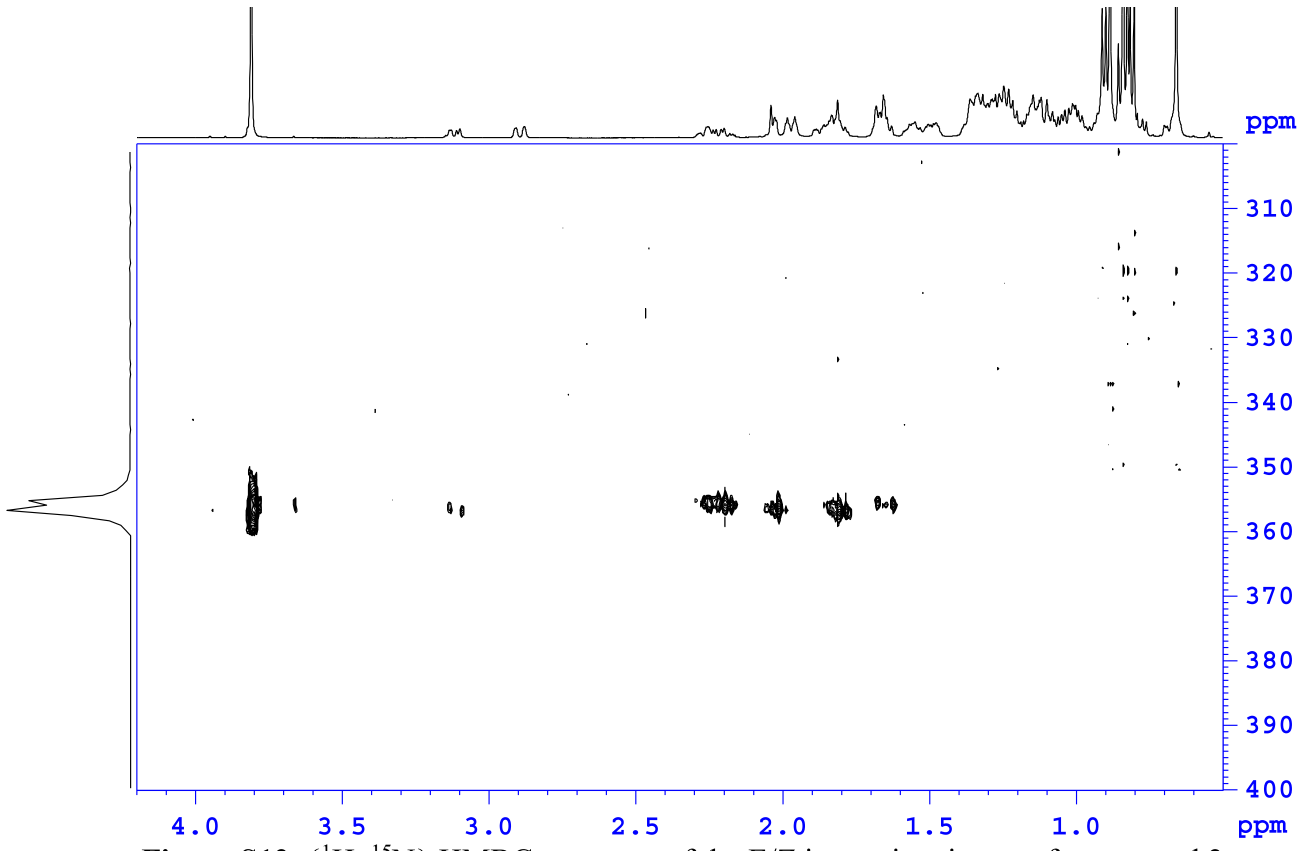

Figure S12. {^{1}H, ^{15}N} HMBC spectrum of the E/Z isomeric mixture of compound **2**.

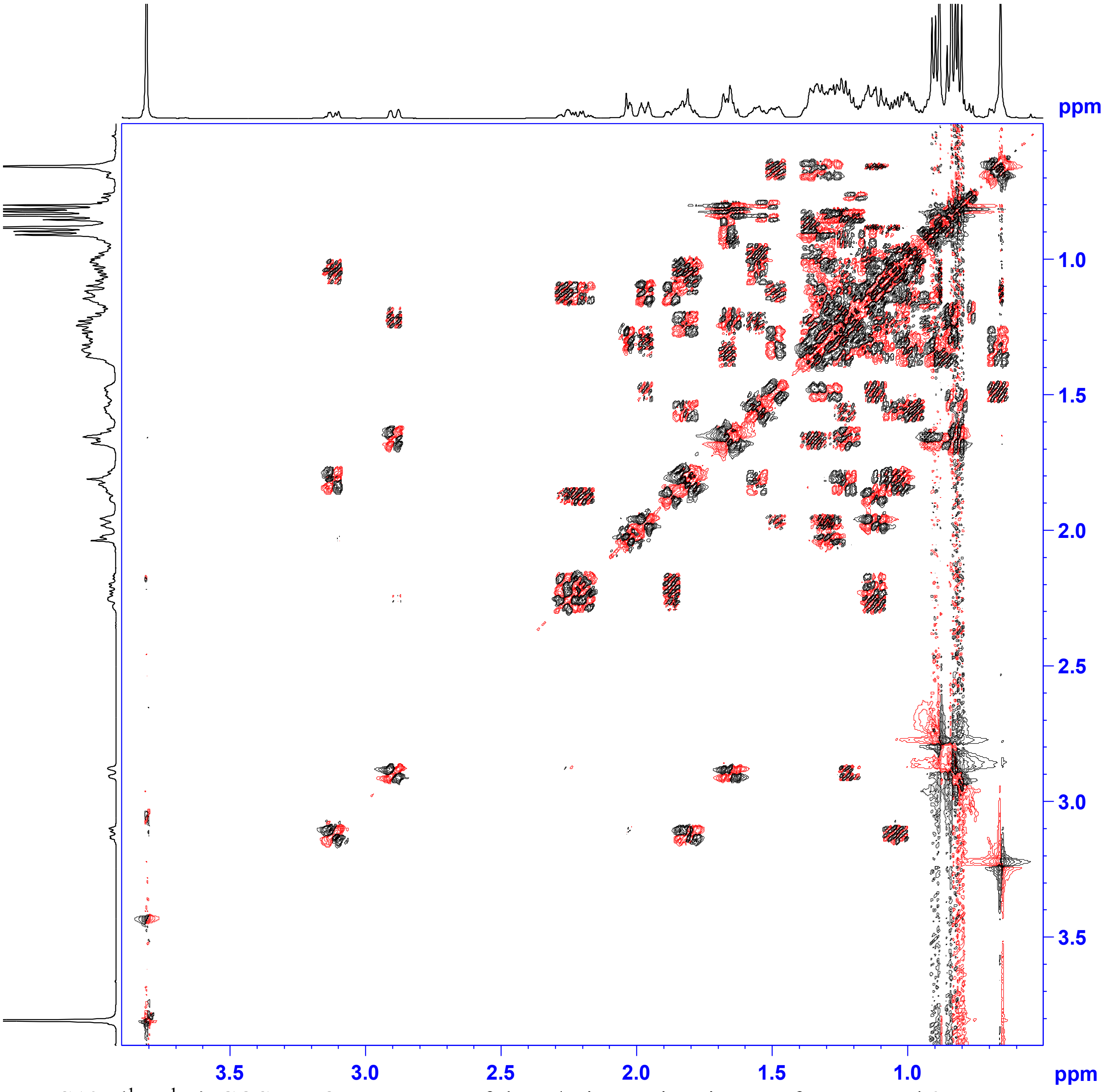

Figure S13. {^{1}H, ^{1}H} COSY-DQF spectrum of the E/Z isomeric mixture of compound **2**.

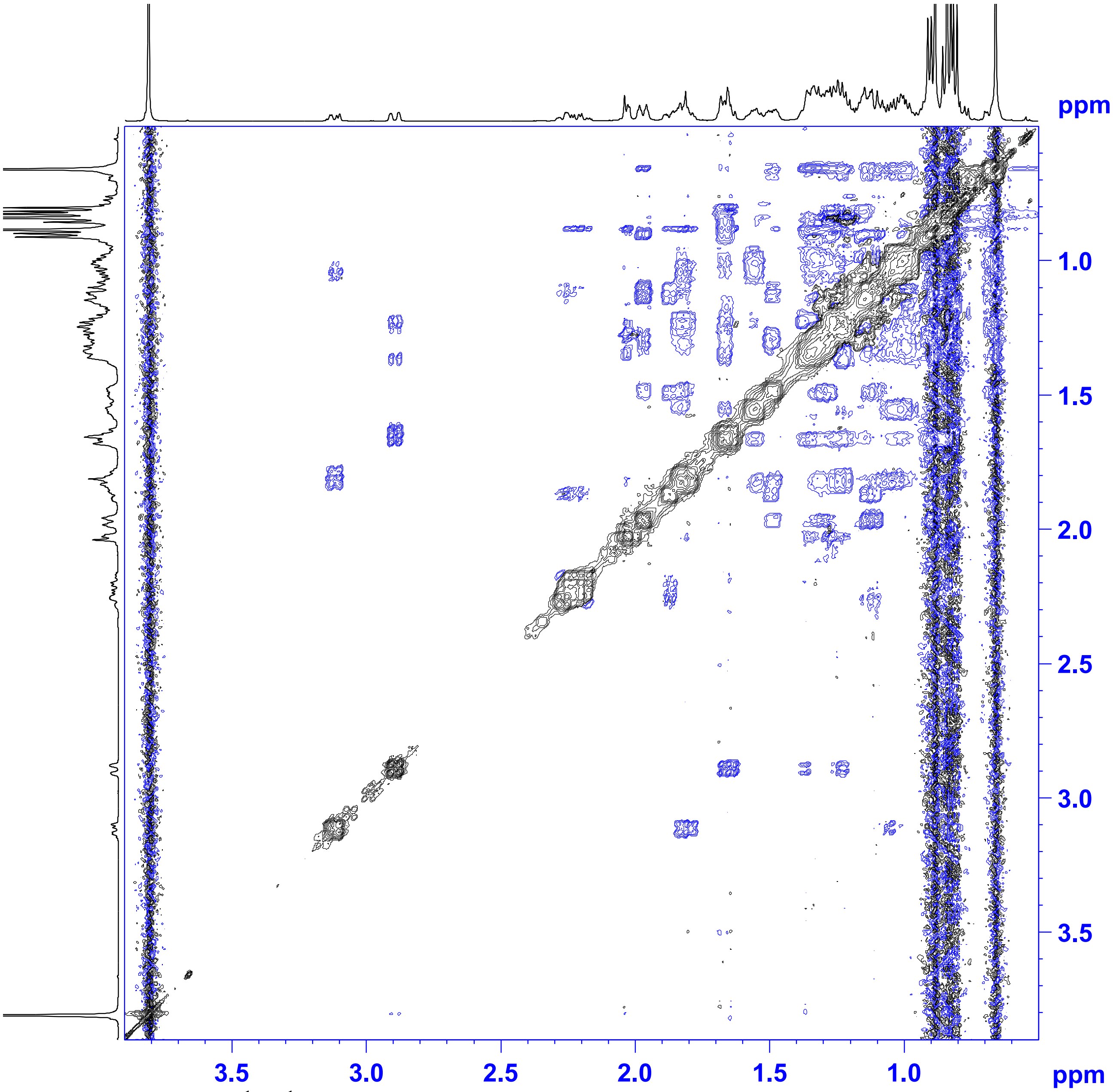

Figure S14. {^{1}H, ^{1}H} NOESY spectrum of the E/Z isomeric mixture of compound **2**.

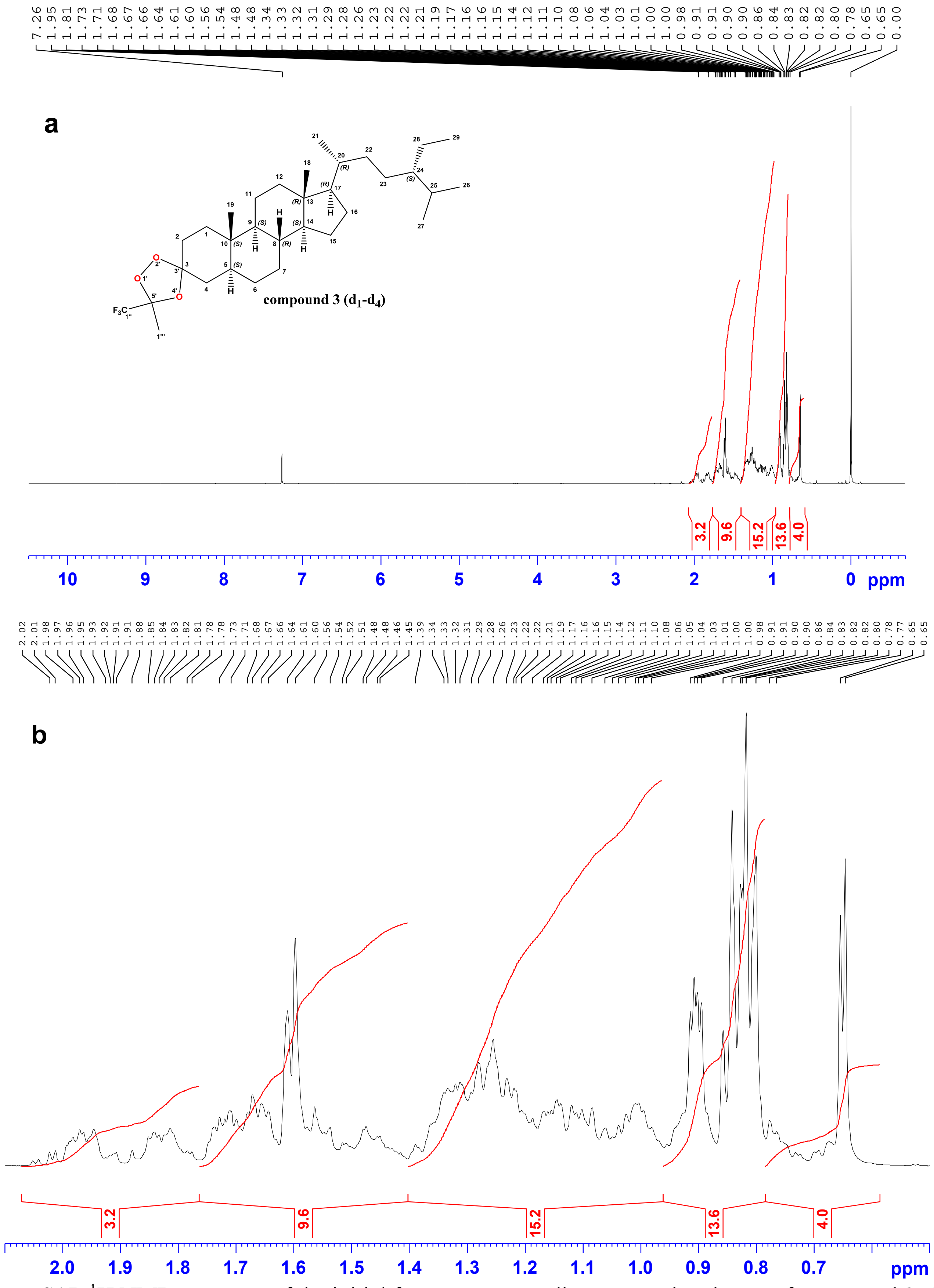

Figure S15. ^{1}H NMR spectrum of the initial four-component diastereomeric mixture of compound **3**: (a) full spectrum; (b) expanded region.

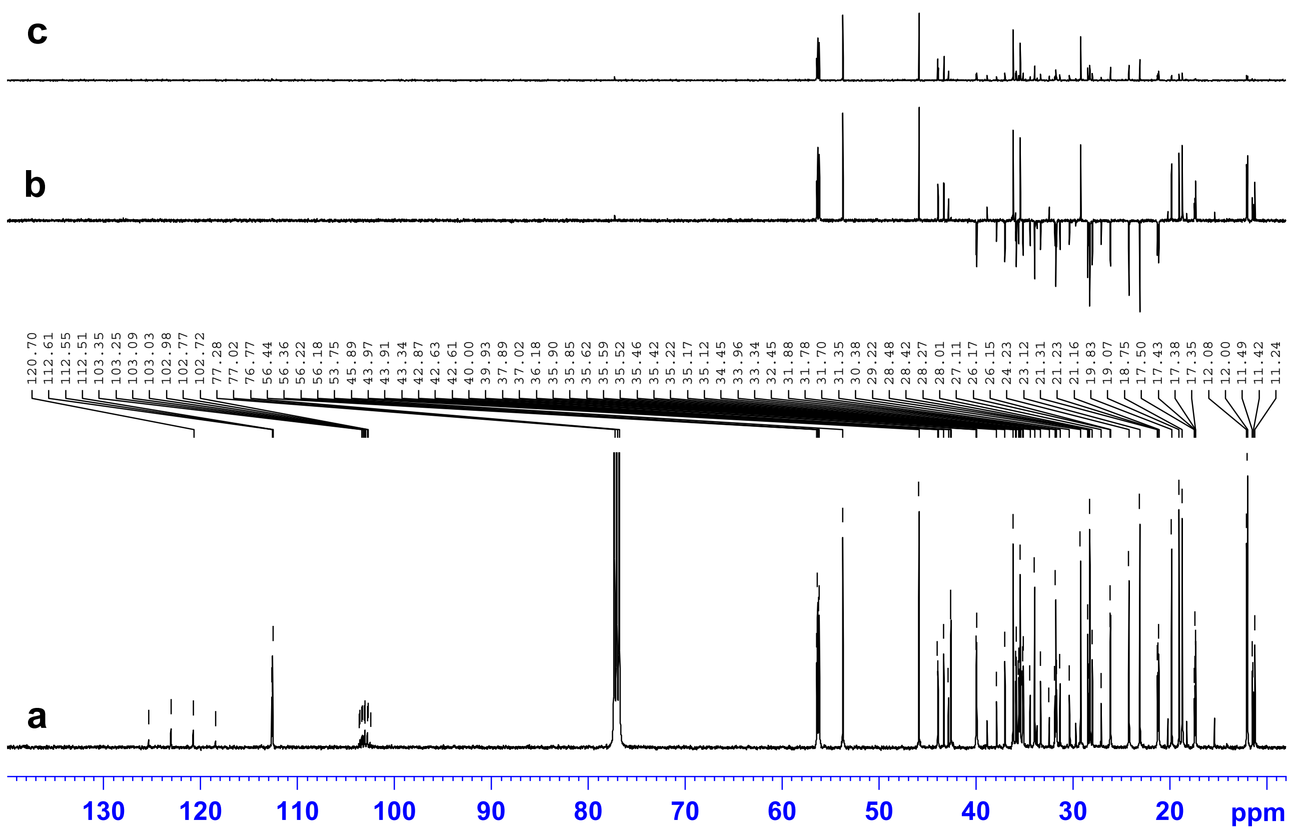

Figure S16. ^{13}C NMR spectra of the initial four-component diastereomeric mixture of compound **3**: (a) $^{13}C\{^1H\}$, (b) DEPT-135, (c) DEPT-90.

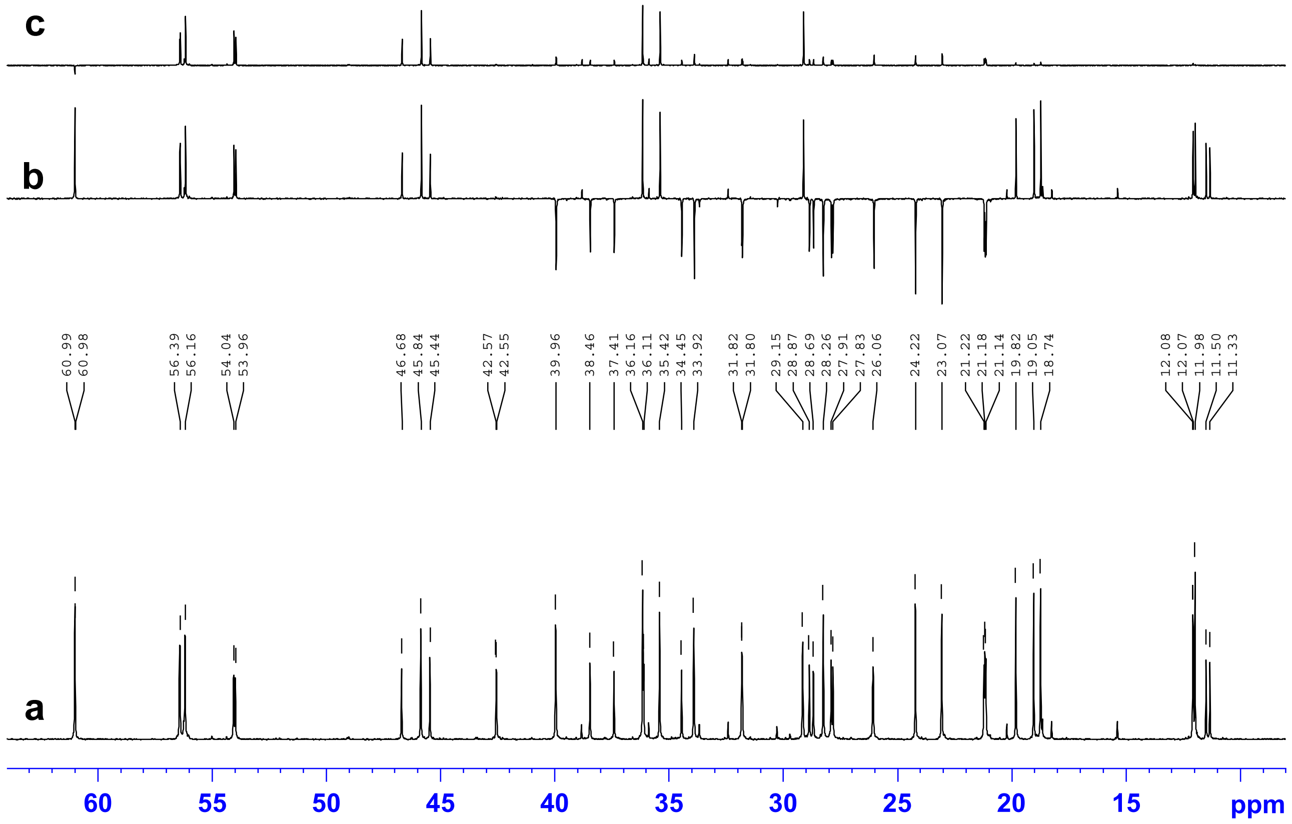

Figure S17. Expanded ^{13}C NMR spectra of the initial four-component diastereomeric mixture of compound **3**: (a) $^{13}C\{^1H\}$, (b) DEPT-135, (c) DEPT-90.

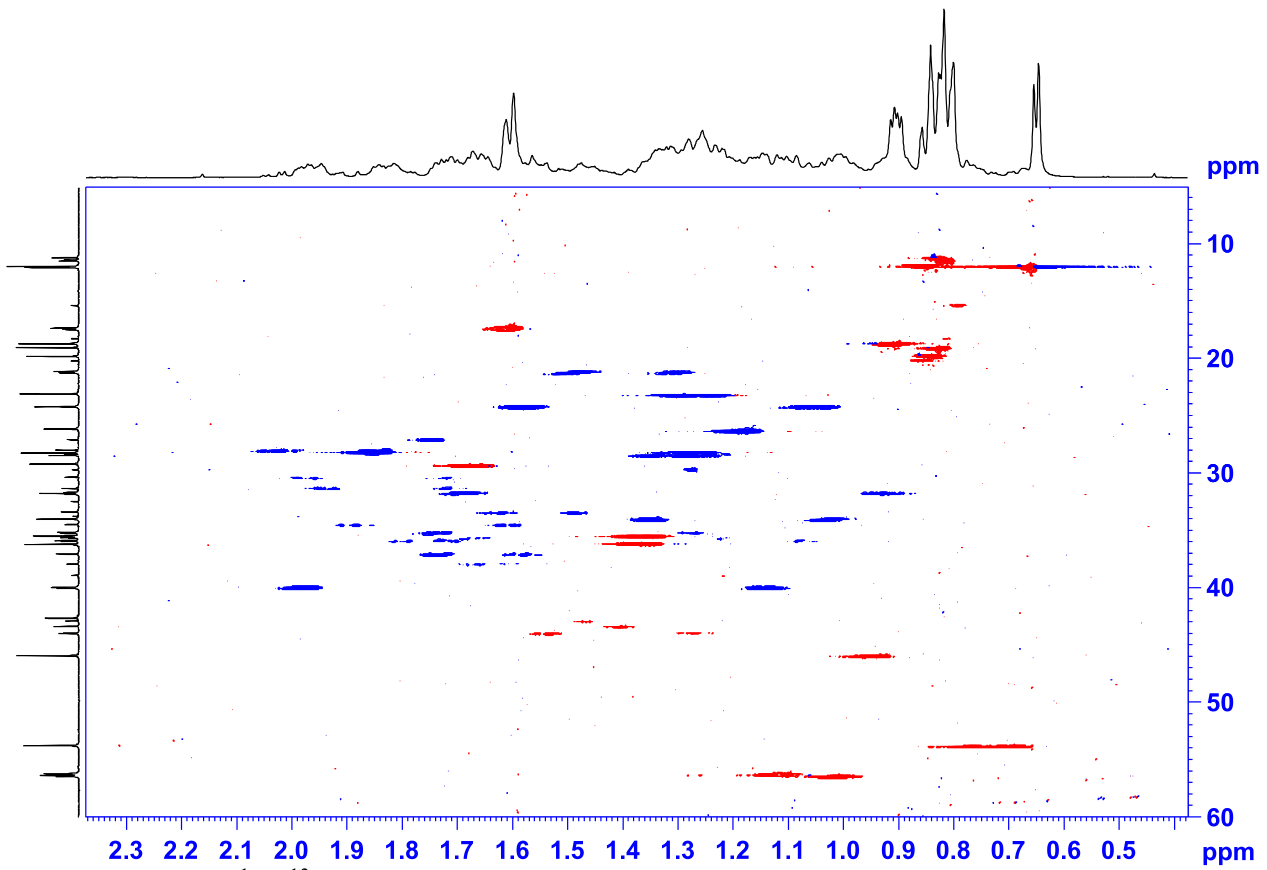

Figure S18. {^{1}H, ^{13}C} HSQCed spectrum of the initial four-component diastereomeric mixture of compound **3**.

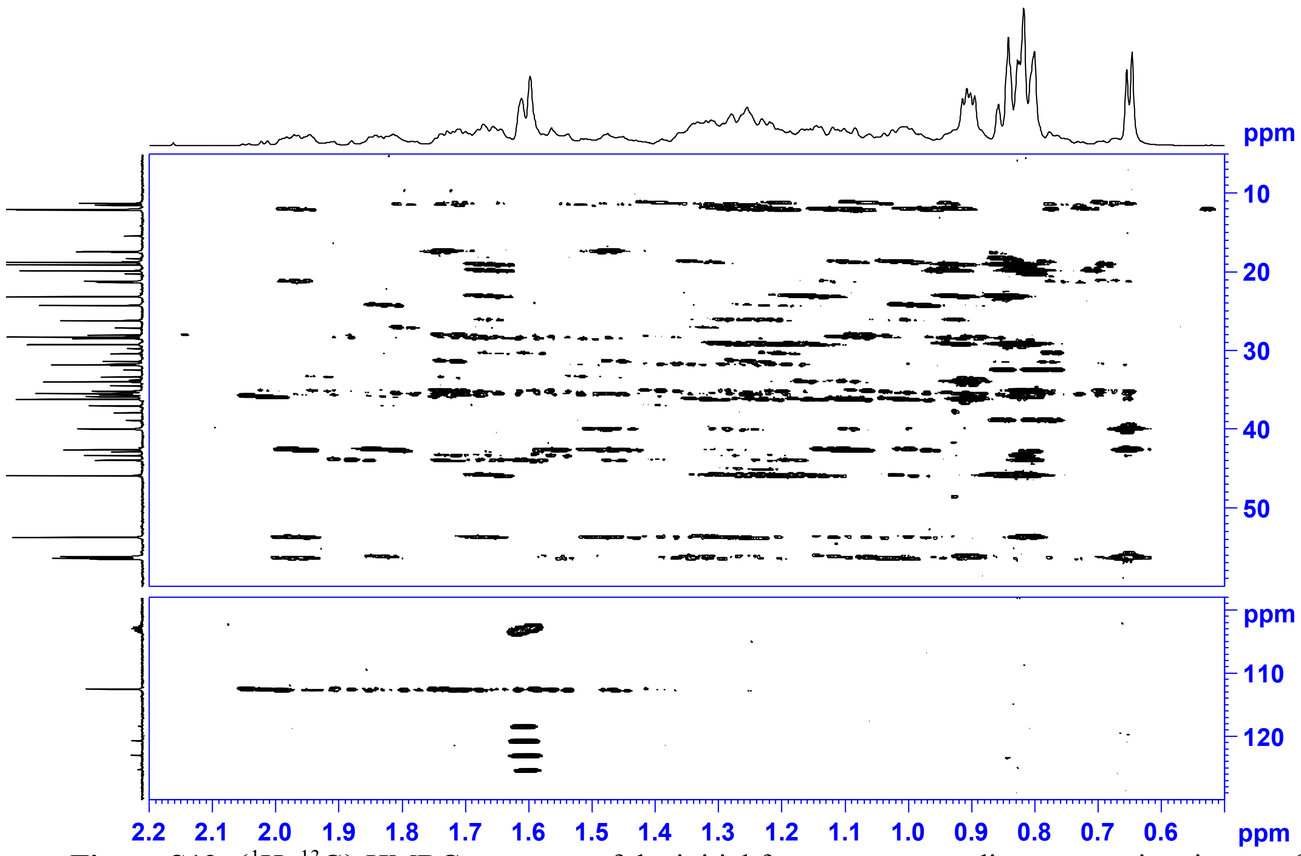

Figure S19. {^{1}H, ^{13}C} HMBC spectrum of the initial four-component diastereomeric mixture of compound **3**.

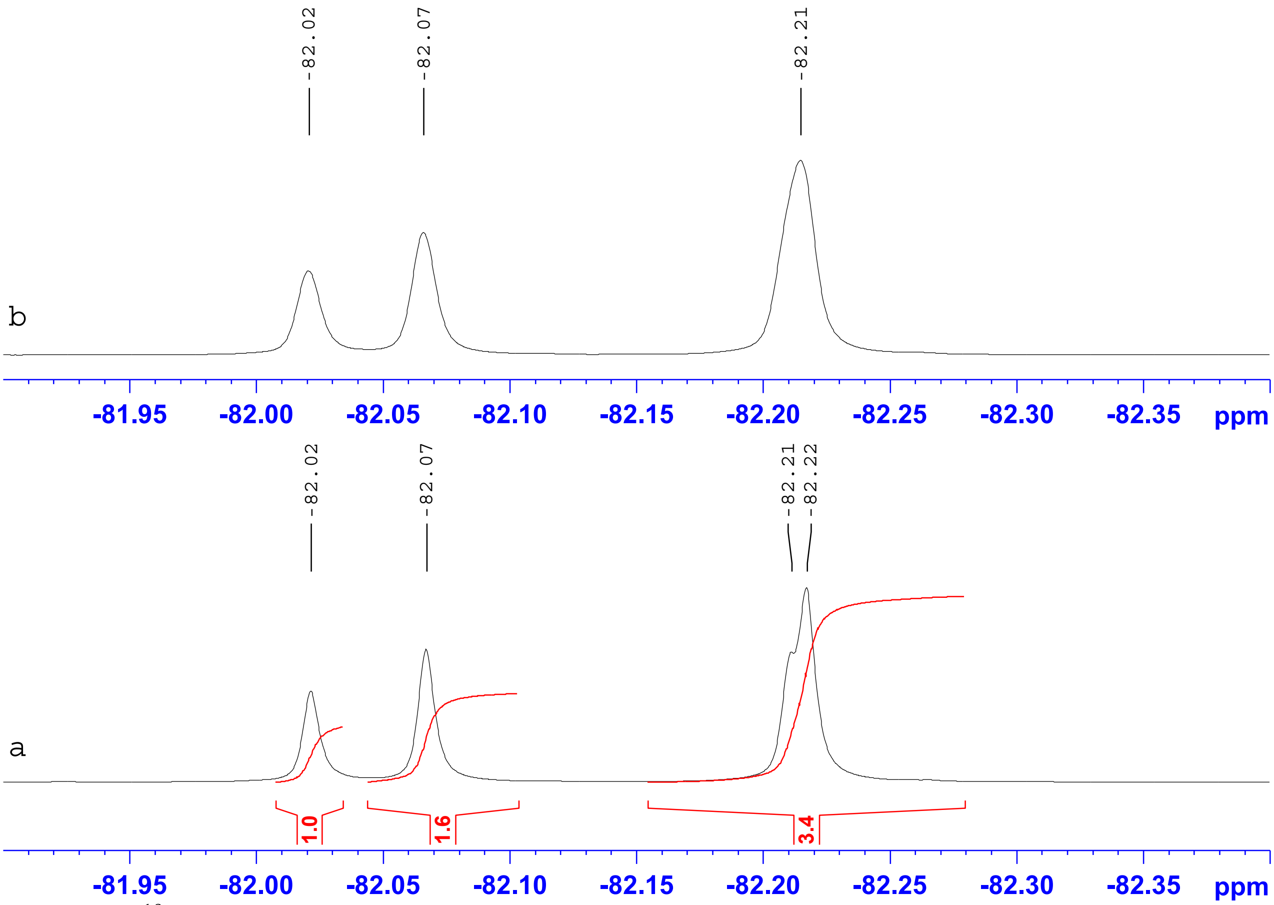

Figure S20. ^{19}F NMR spectra of the initial four-component diastereomeric mixture of compound **3**: (a) $^{19}F\{^{1}H\}$, (b) ^{19}F.

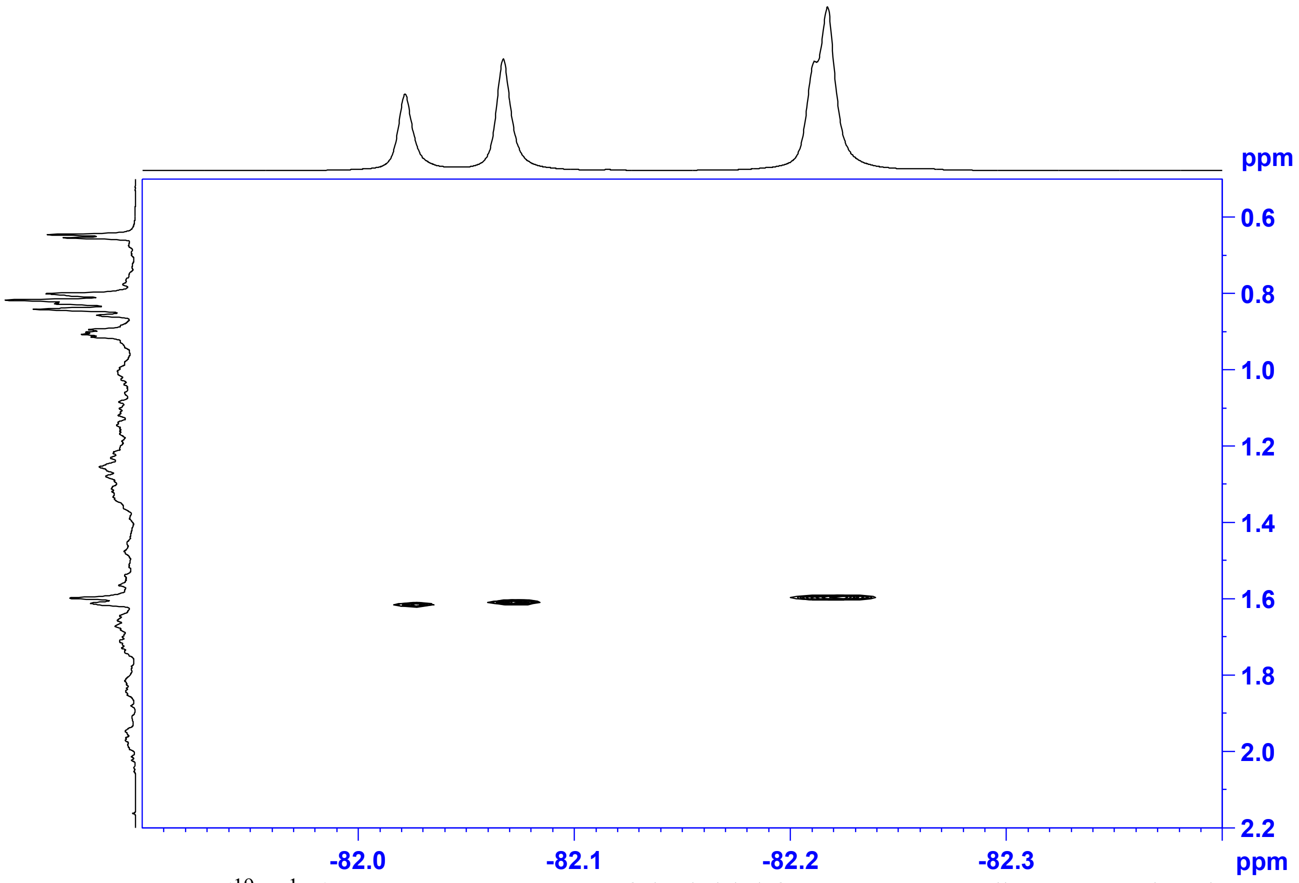

Figure S21. $\{^{19}F, ^{1}H\}$ HETCOR spectrum of the initial four-component diastereomeric mixture of compound **3**.

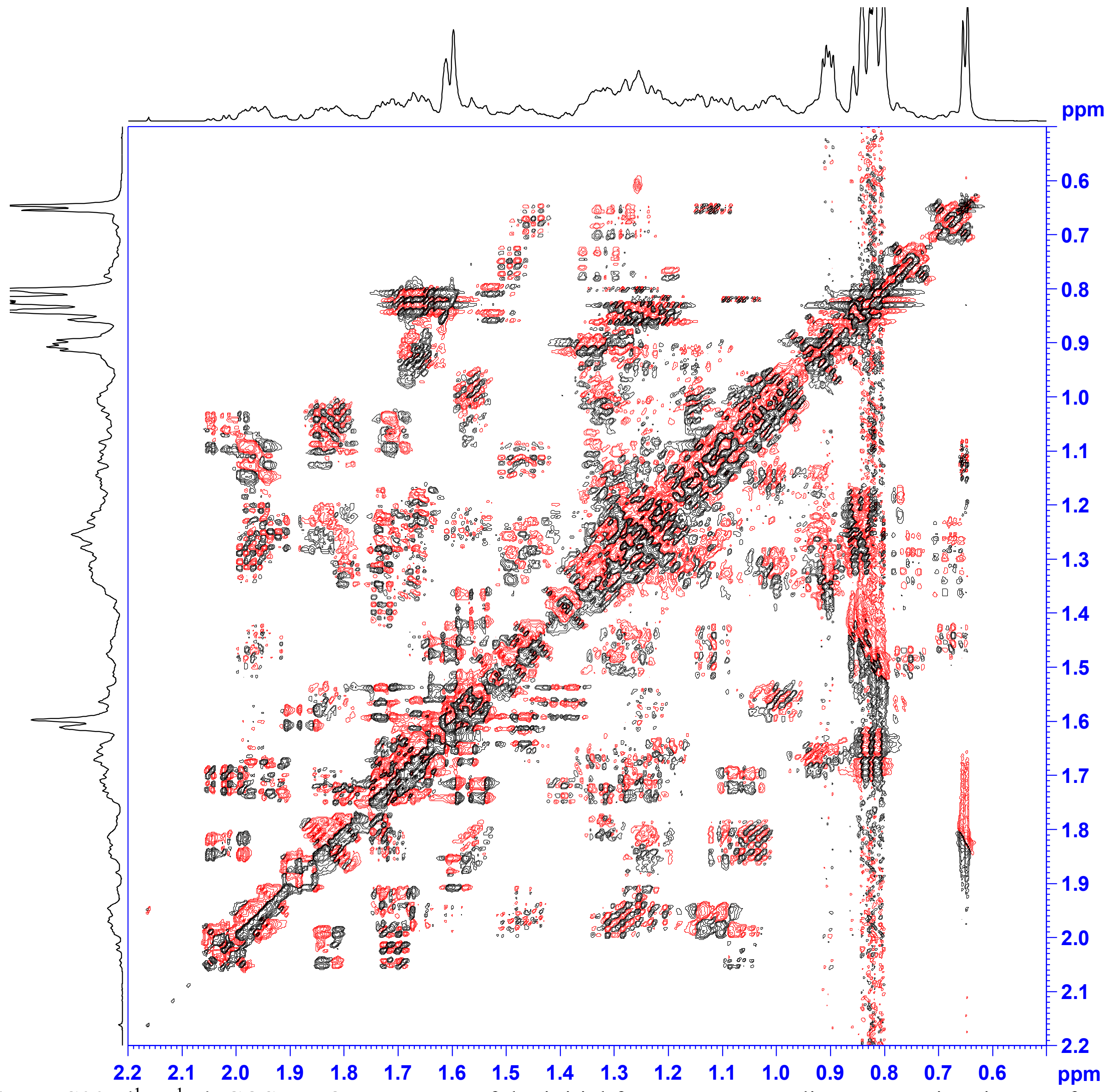

Figure S22. {^{1}H, ^{1}H} COSY-DQF spectrum of the initial four-component diastereomeric mixture of compound **3**.

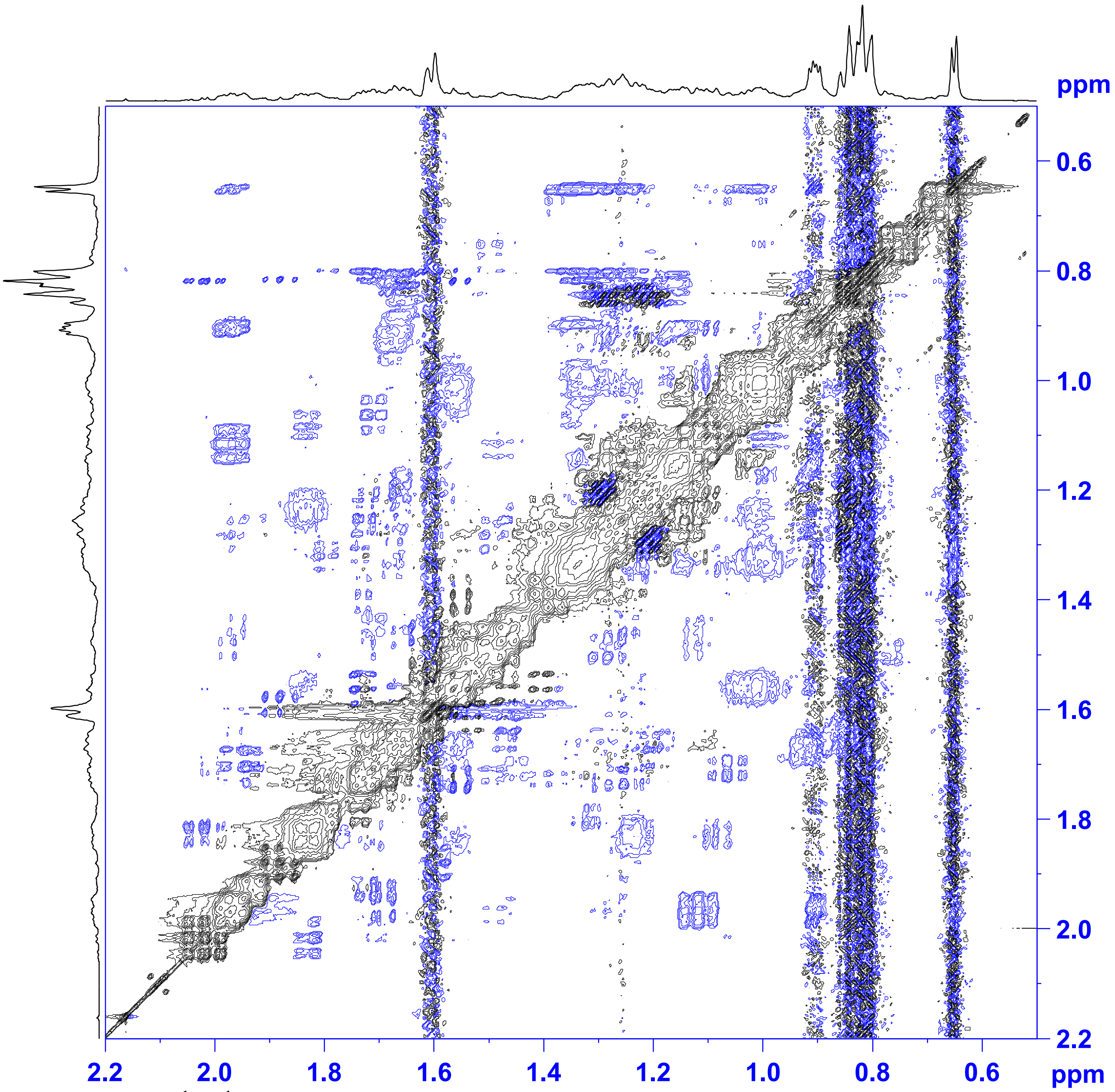

Figure S23. {^{1}H, ^{1}H} NOESY spectrum of the initial four-component diastereomeric mixture of compound **3**.

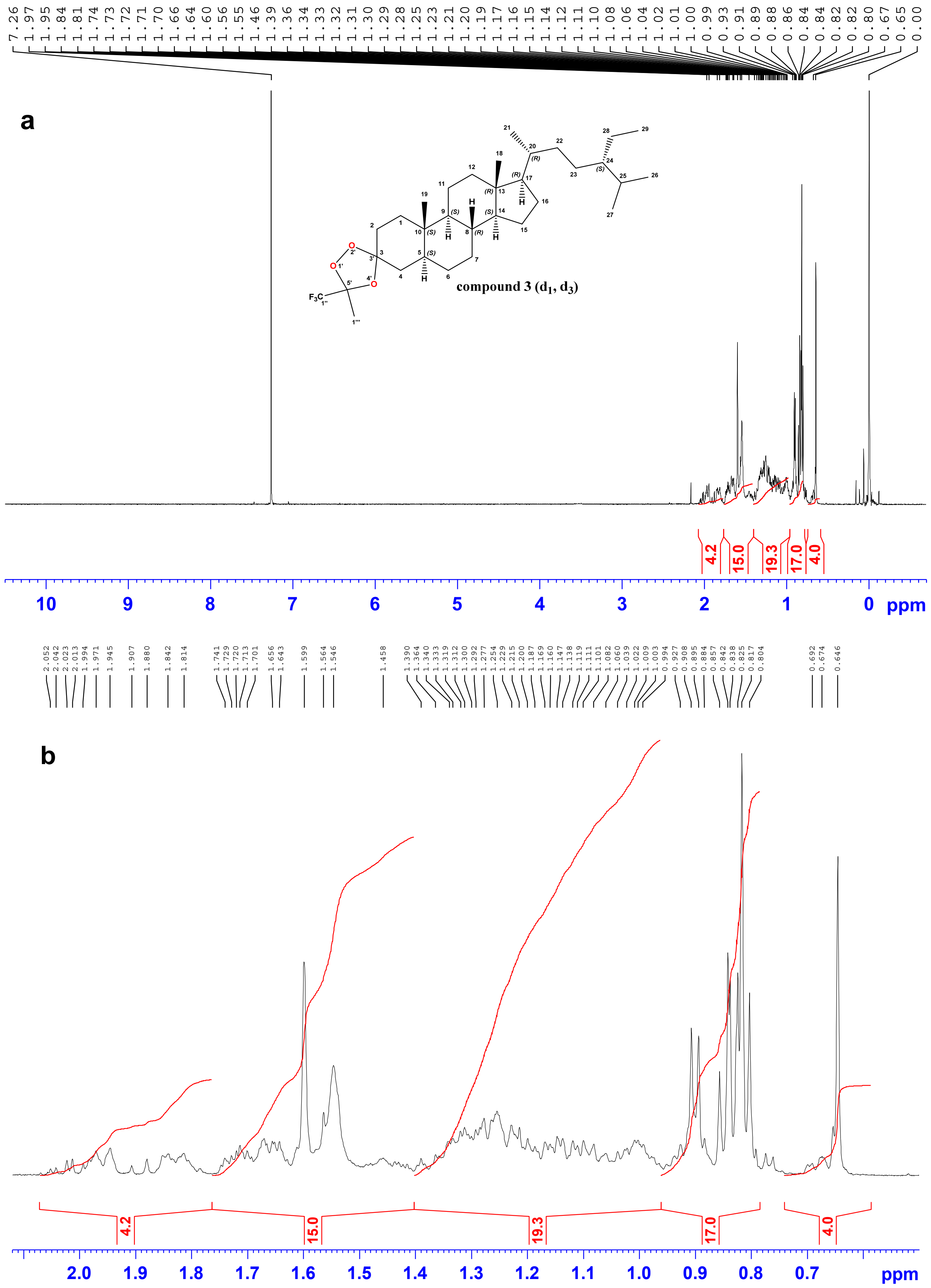

Figure S24. ^{1}H NMR spectrum of the isolated α-isomer pair of compound **3**: (a) full spectrum; (b) expanded region.

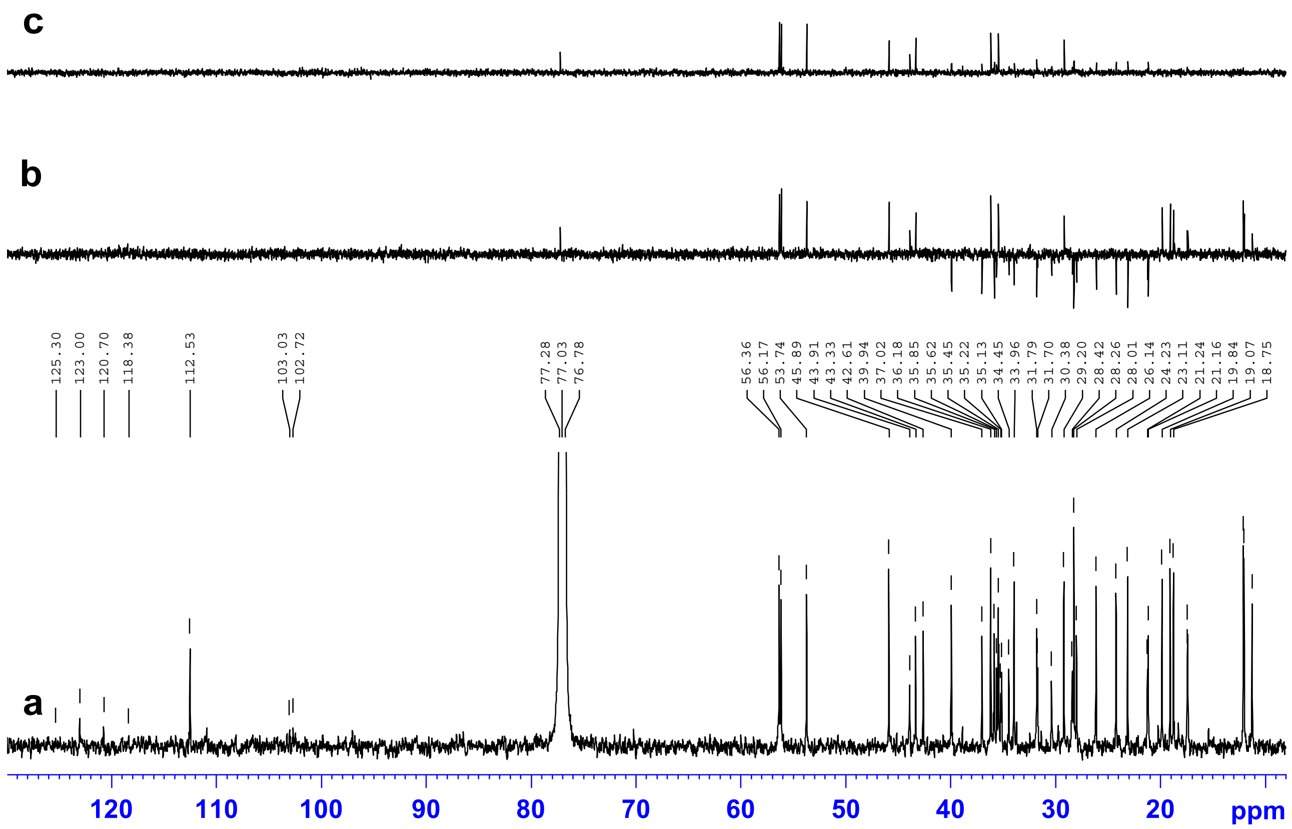

Figure S25. ^{13}C NMR spectra of the isolated α-isomer pair of compound **3**: (a) $^{13}C\{^1H\}$, (b) DEPT-135, (c) DEPT-90.

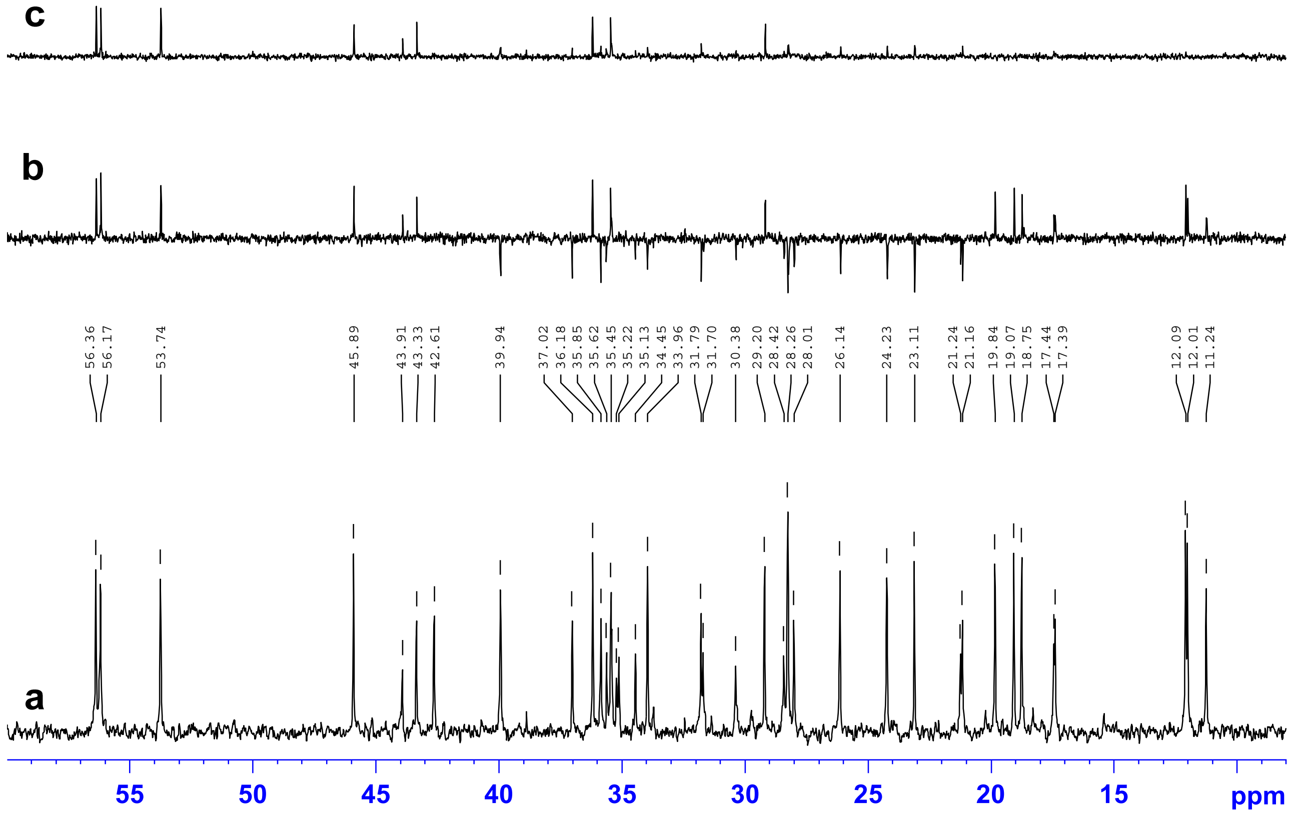

Figure S26. Expanded ^{13}C NMR spectra of the isolated α-isomer pair of compound **3**: (a) $^{13}C\{^1H\}$, (b) DEPT-135, (c) DEPT-90.

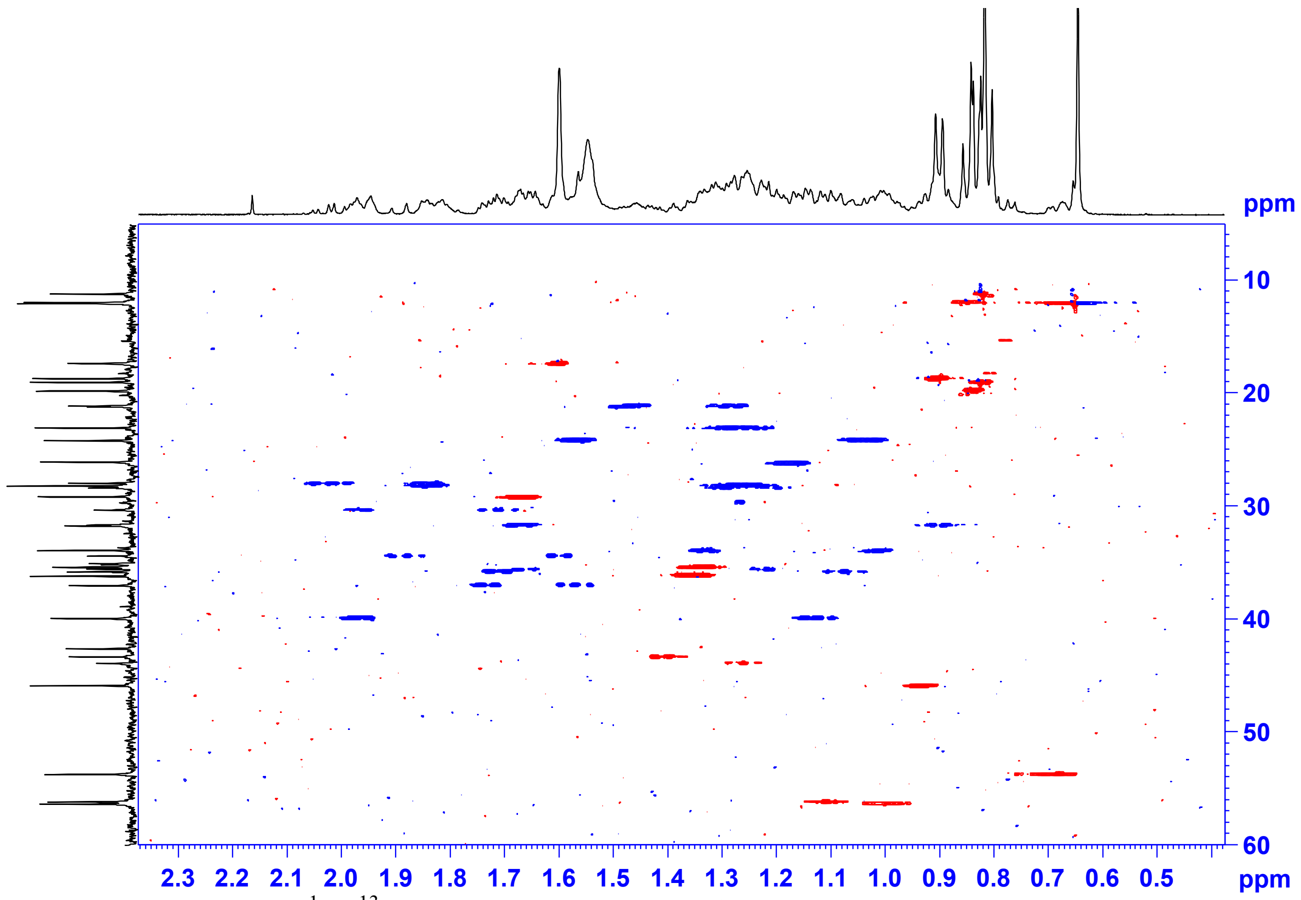

Figure S27. {^{1}H, ^{13}C} HSQCed spectrum of the isolated α-isomer pair of compound **3**.

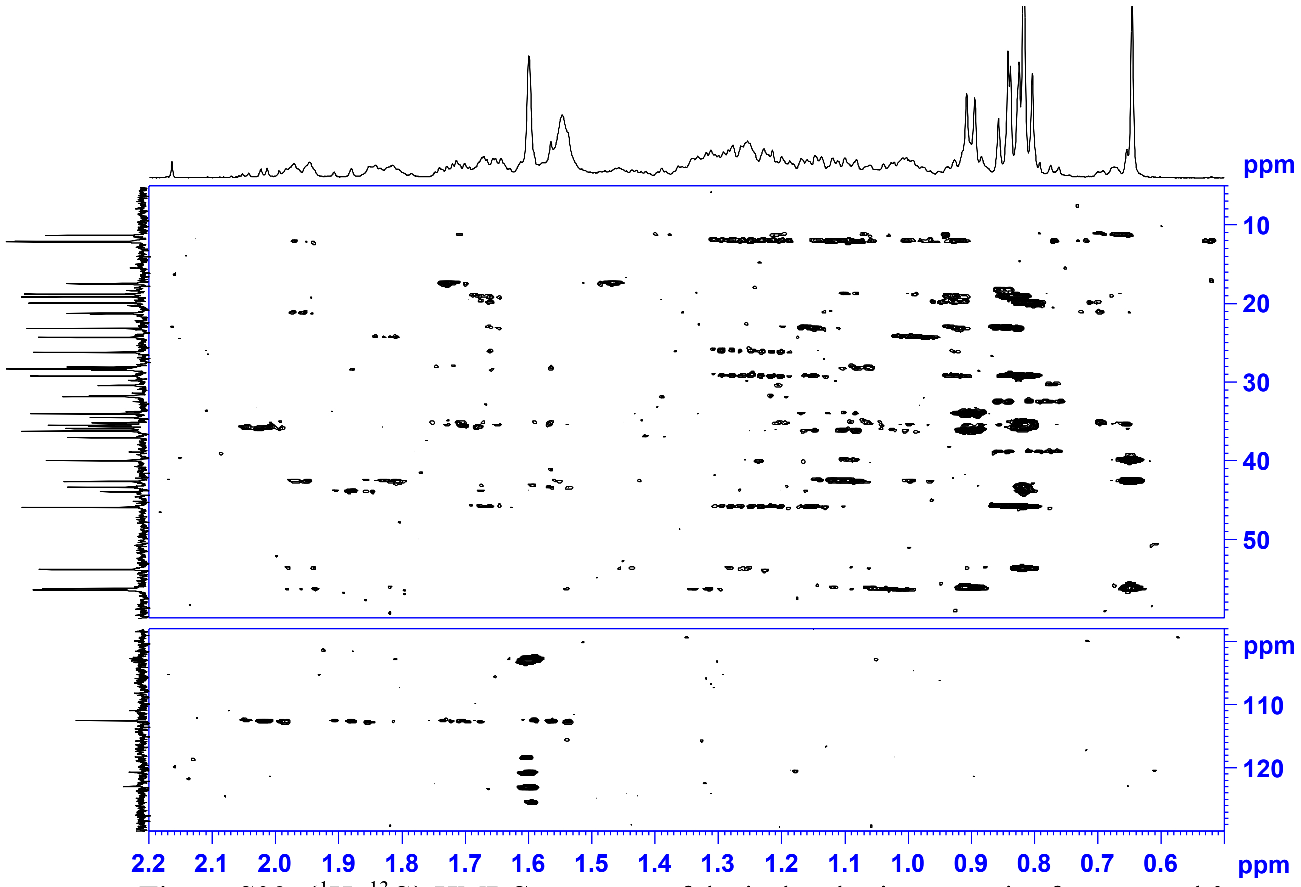

Figure S28. {^{1}H, ^{13}C} HMBC spectrum of the isolated α-isomer pair of compound **3**.

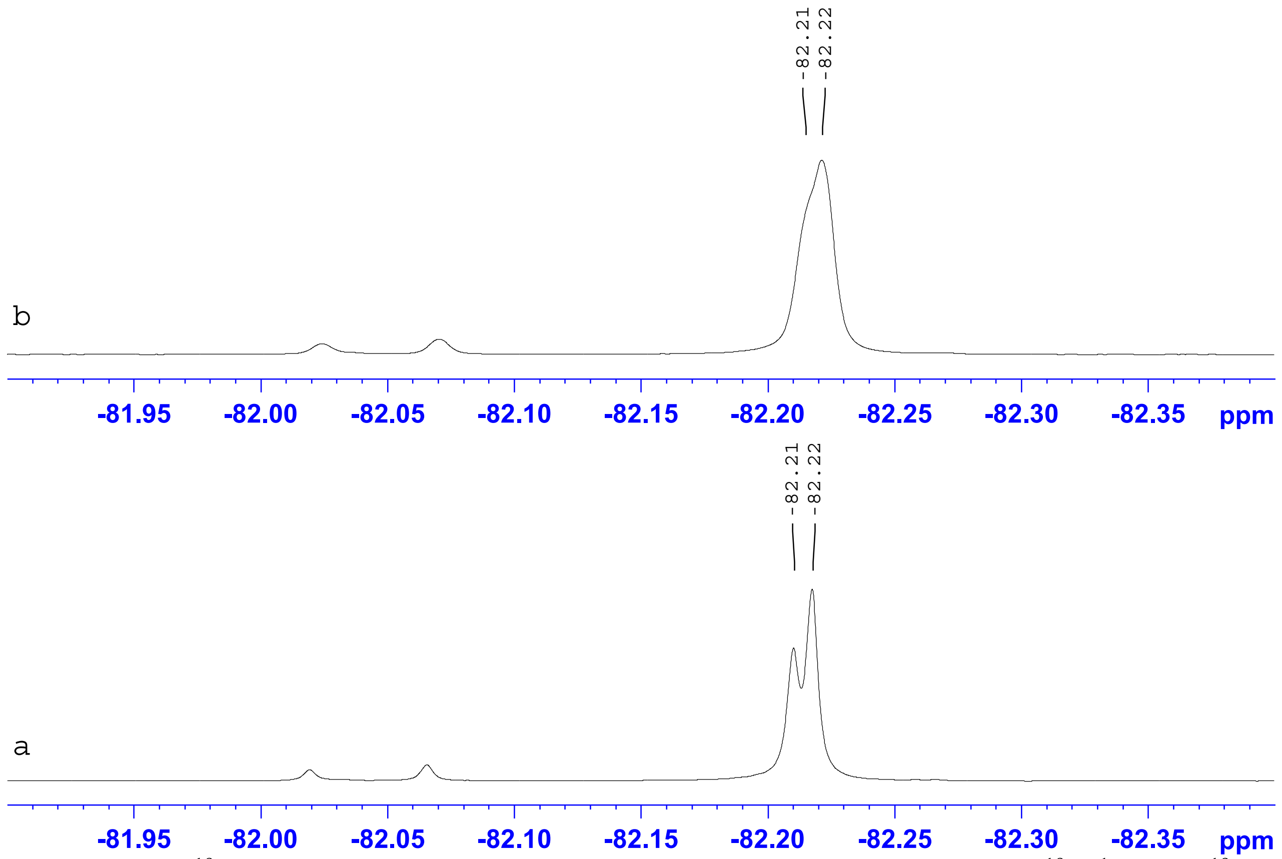

Figure S29. ^{19}F NMR spectra of the isolated α-isomer pair of compound **3**: (a) $^{19}F\{^{1}H\}$, (b) ^{19}F.

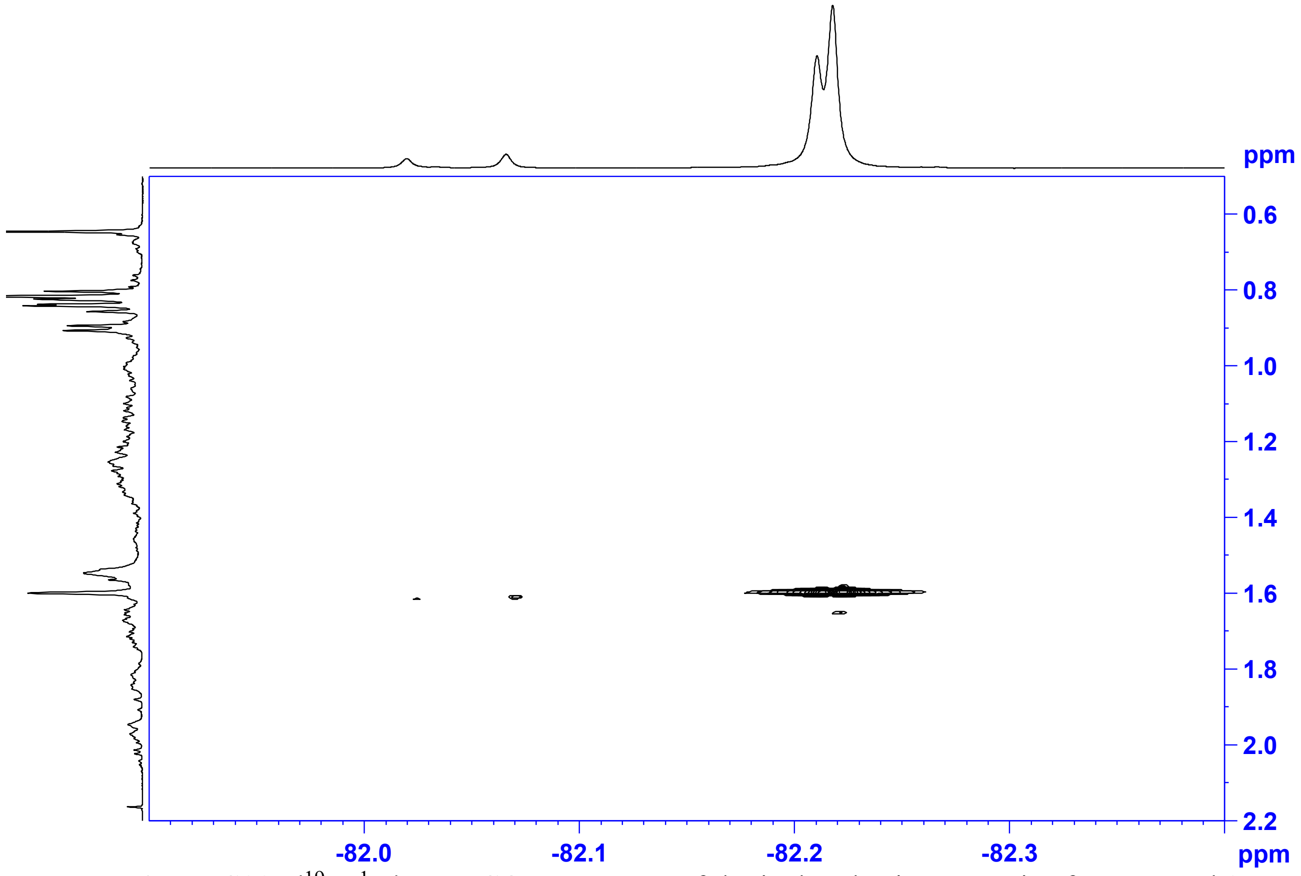

Figure S30. $\{^{19}F, ^{1}H\}$ HETCOR spectrum of the isolated α-isomer pair of compound **3**.

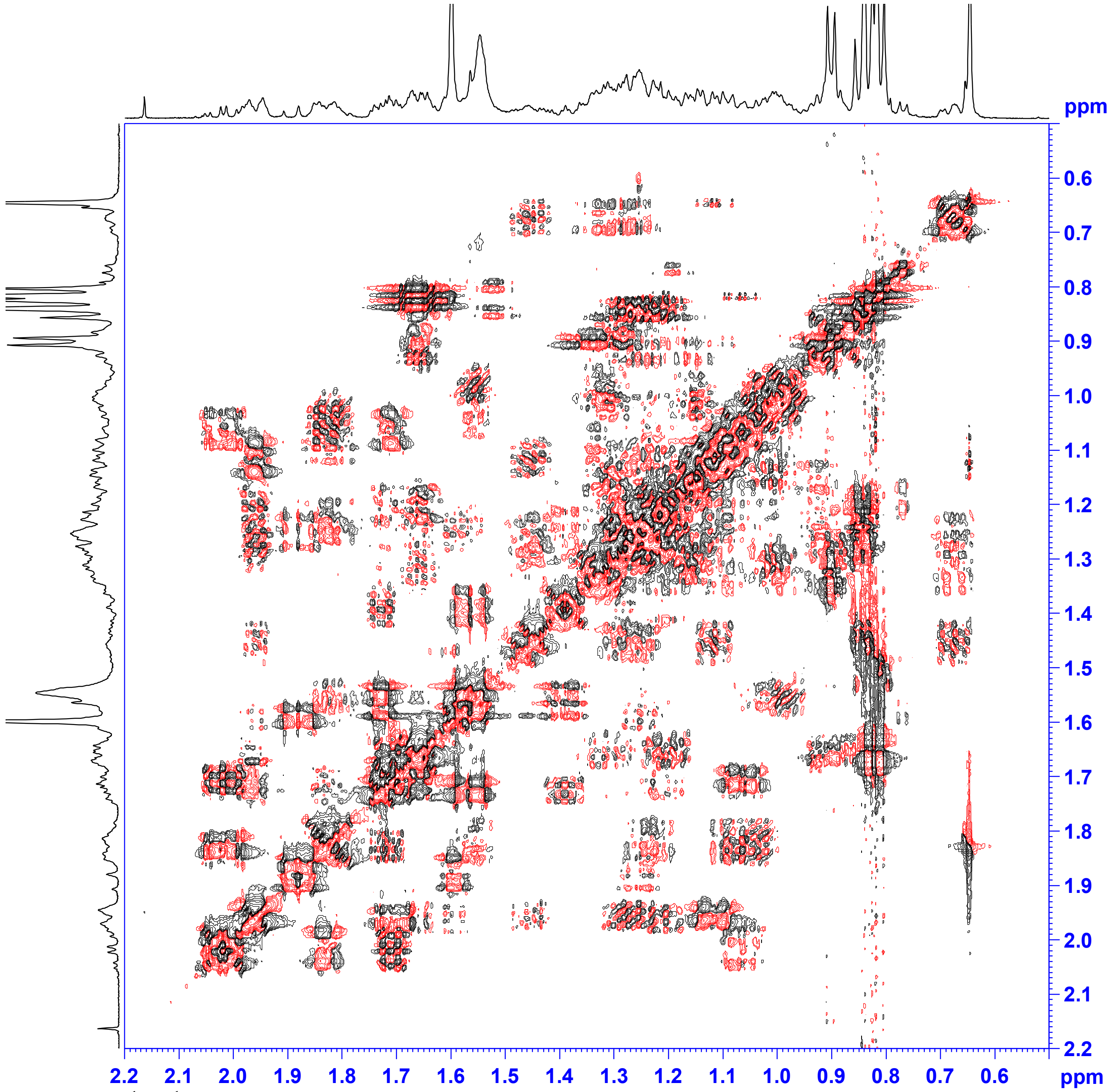

Figure S31. {^{1}H, ^{1}H} COSY-DQF spectrum of the isolated α-isomer pair of compound **3**.

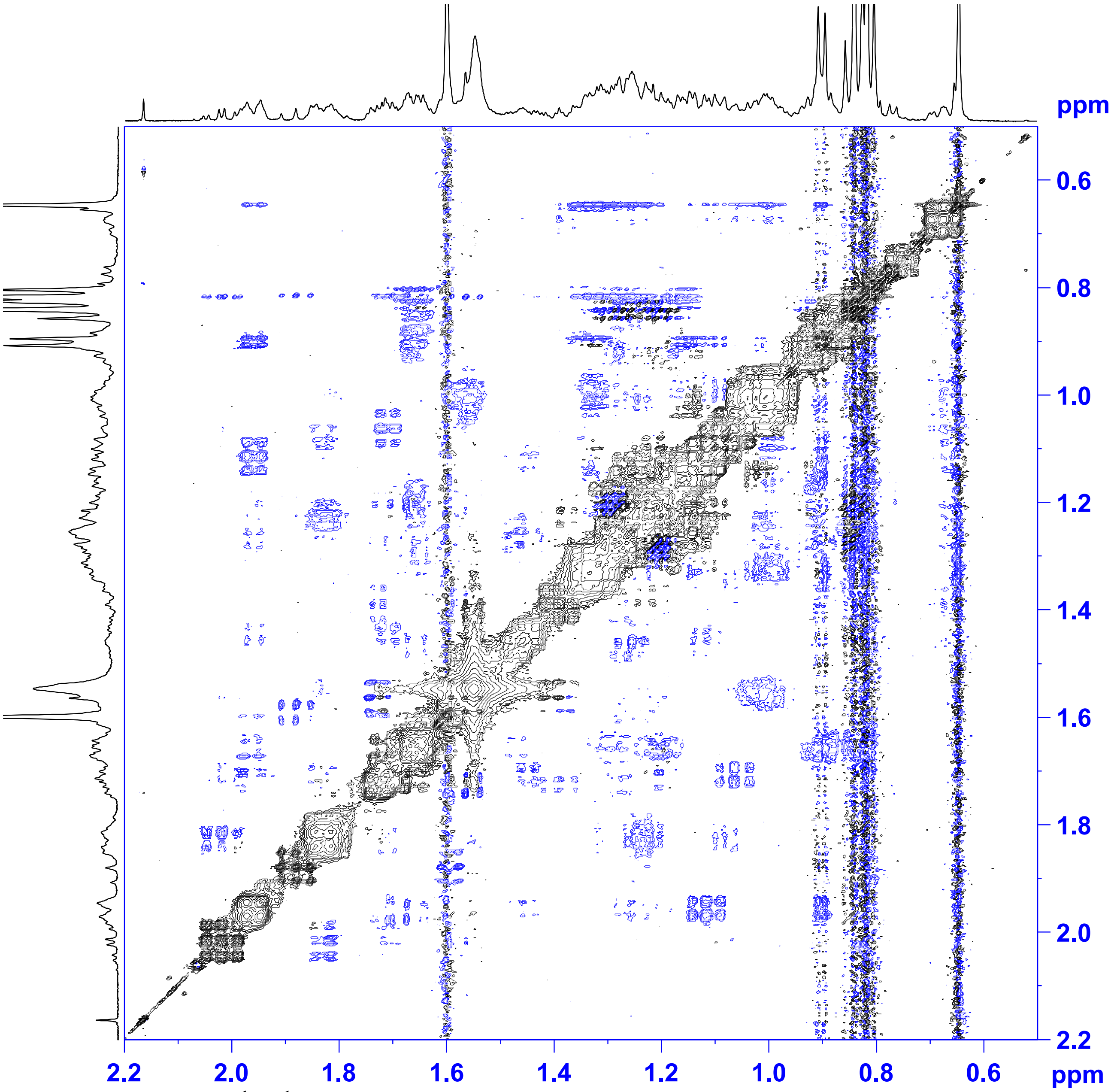

Figure S32. {^{1}H, ^{1}H} NOESY spectrum of the isolated α-isomer pair of compound **3**.

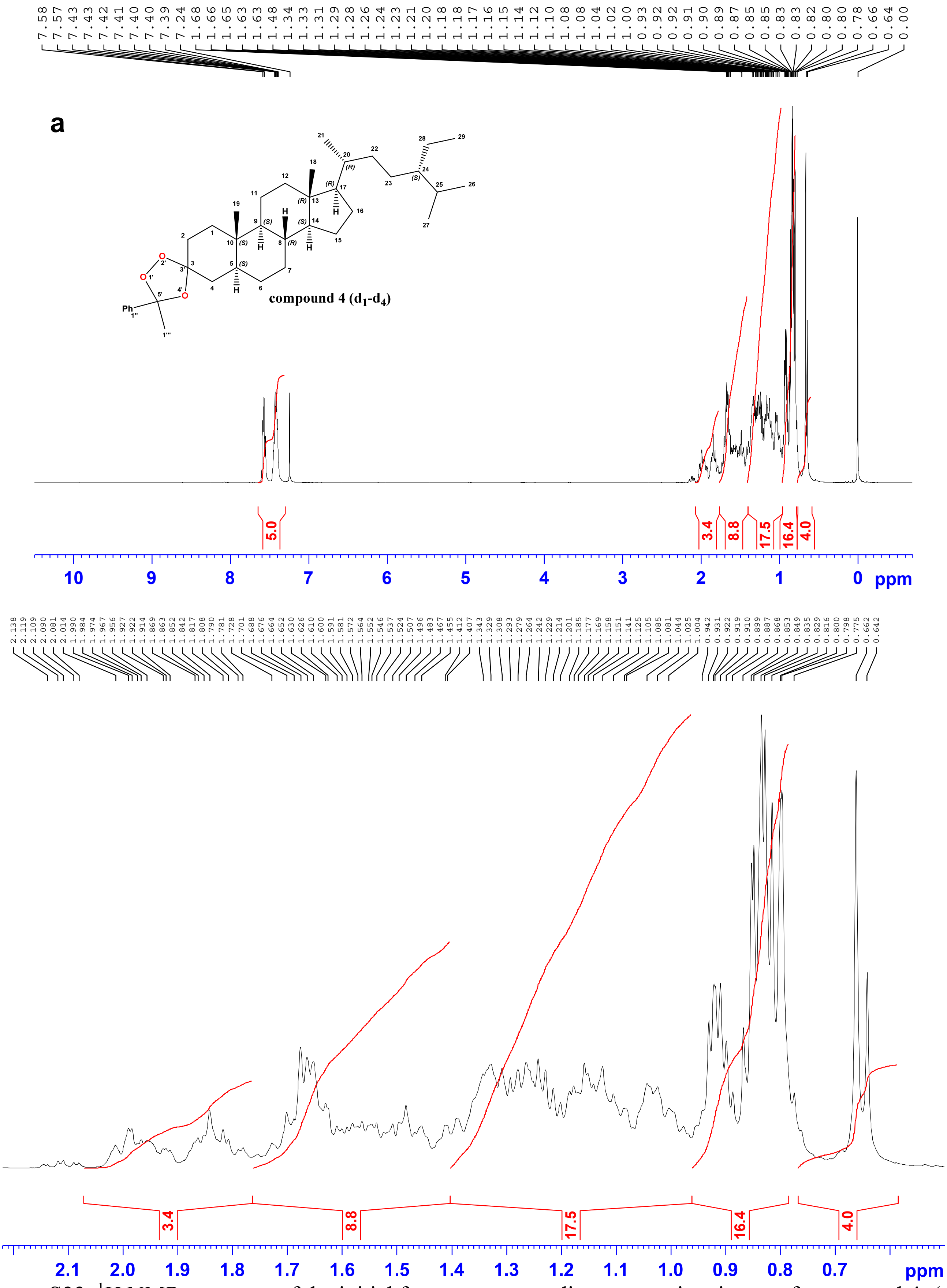

Figure S33. ^{1}H NMR spectrum of the initial four-component diastereomeric mixture of compound **4**: (a) full spectrum; (b) expanded region.

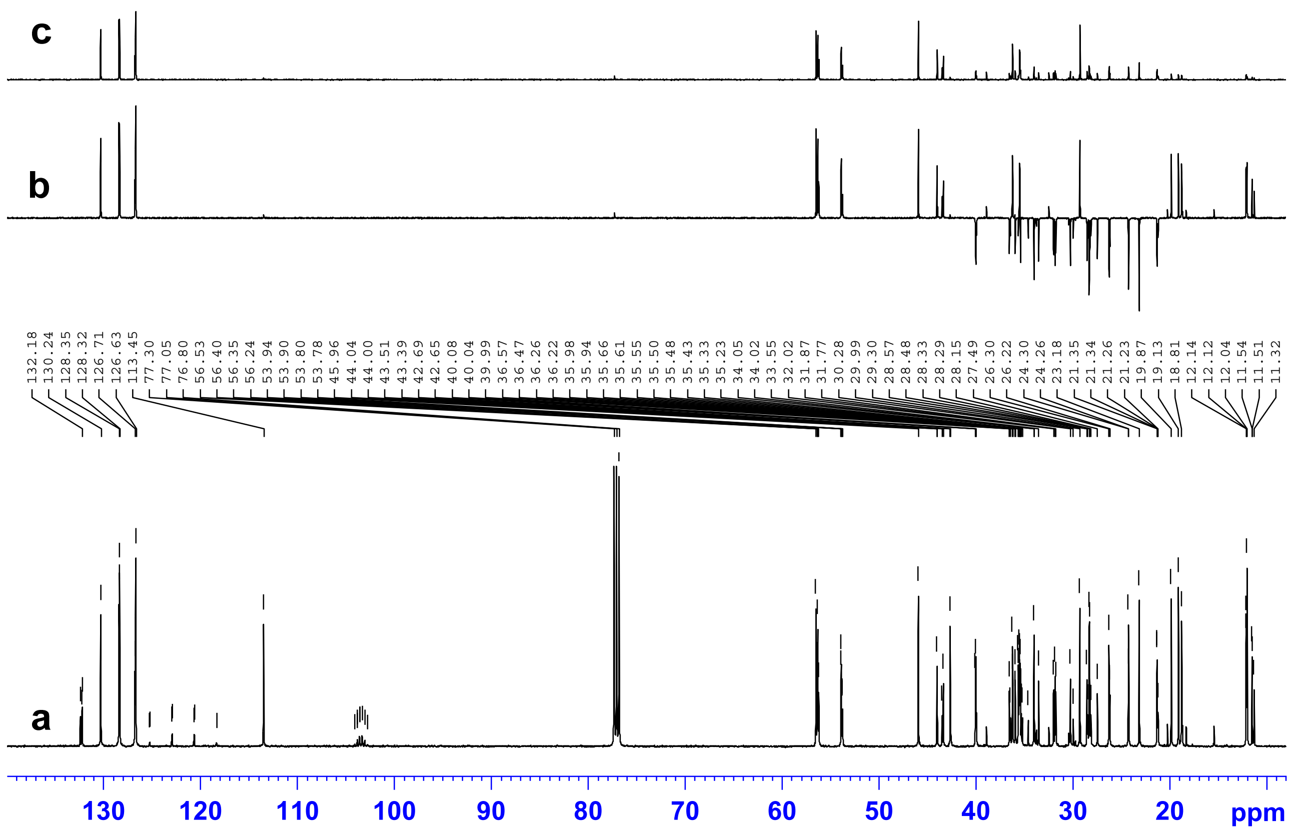

Figure S34. ^{13}C NMR spectra of the initial four-component diastereomeric mixture of compound **4**: (a) $^{13}C\{^1H\}$, (b) DEPT-135, (c) DEPT-90.

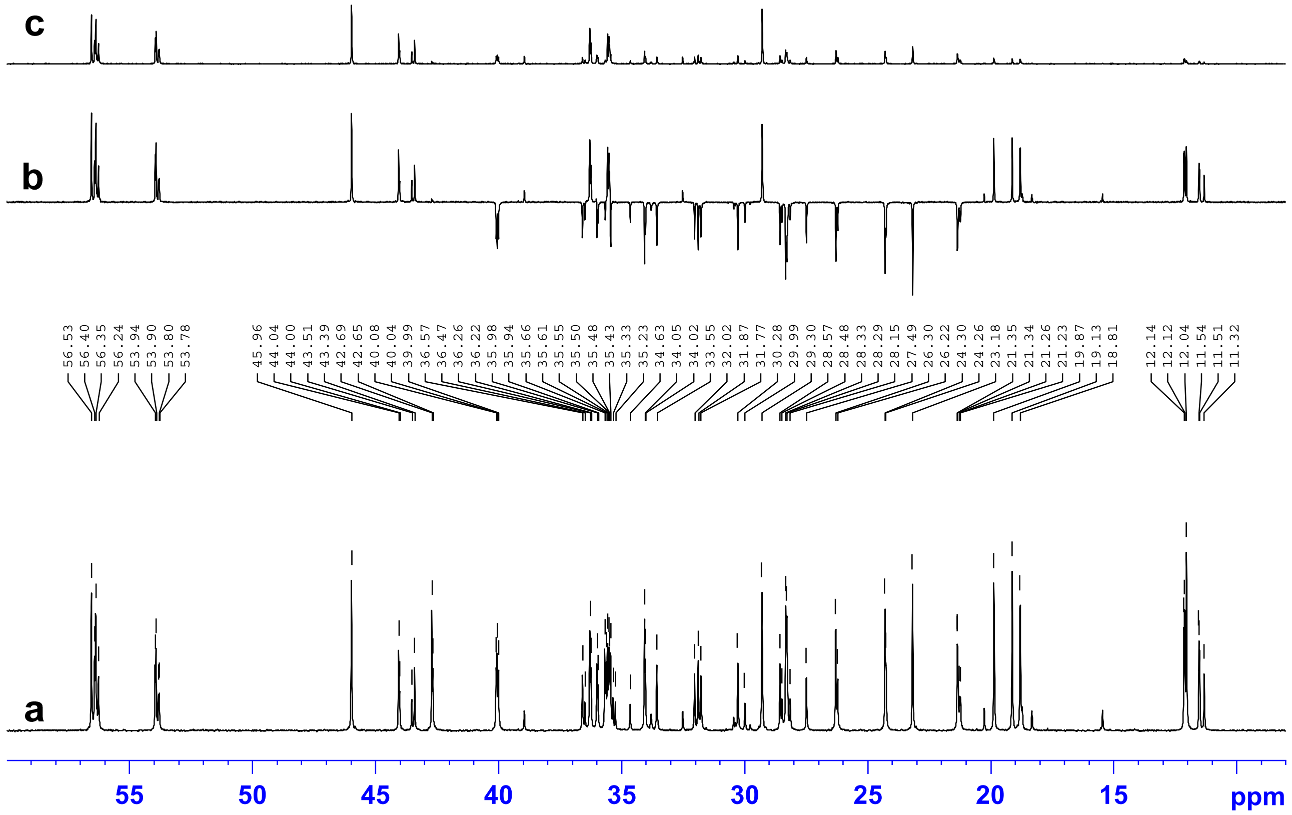

Figure S35. Expanded ^{13}C NMR spectra of the initial four-component diastereomeric mixture of compound **4**: (a) $^{13}C\{^1H\}$, (b) DEPT-135, (c) DEPT-90.

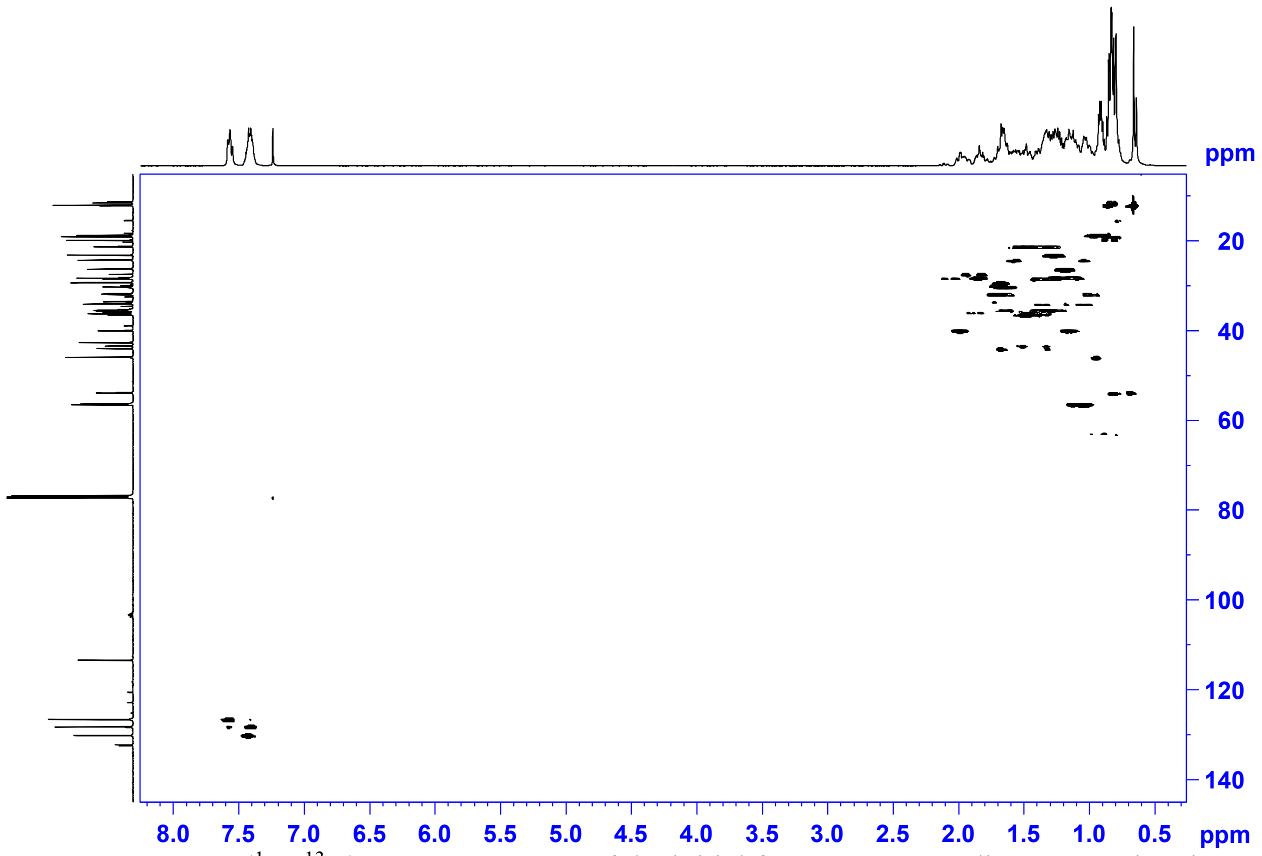

Figure S36. {^{1}H, ^{13}C} HSQC spectrum of the initial four-component diastereomeric mixture of compound **4**.

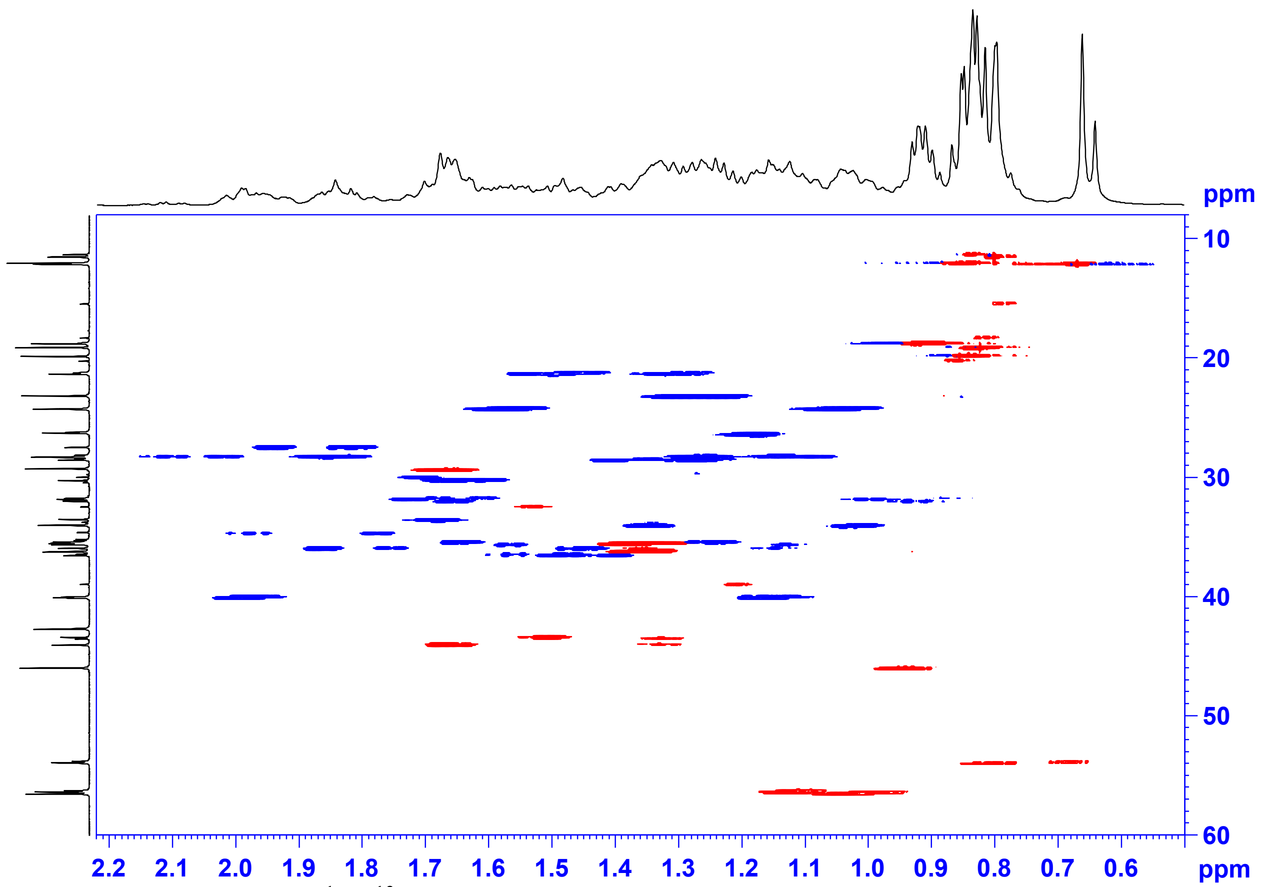

Figure S37. Expanded {^{1}H, ^{13}C} HSQCed spectrum of the initial four-component diastereomeric mixture of compound **4**.

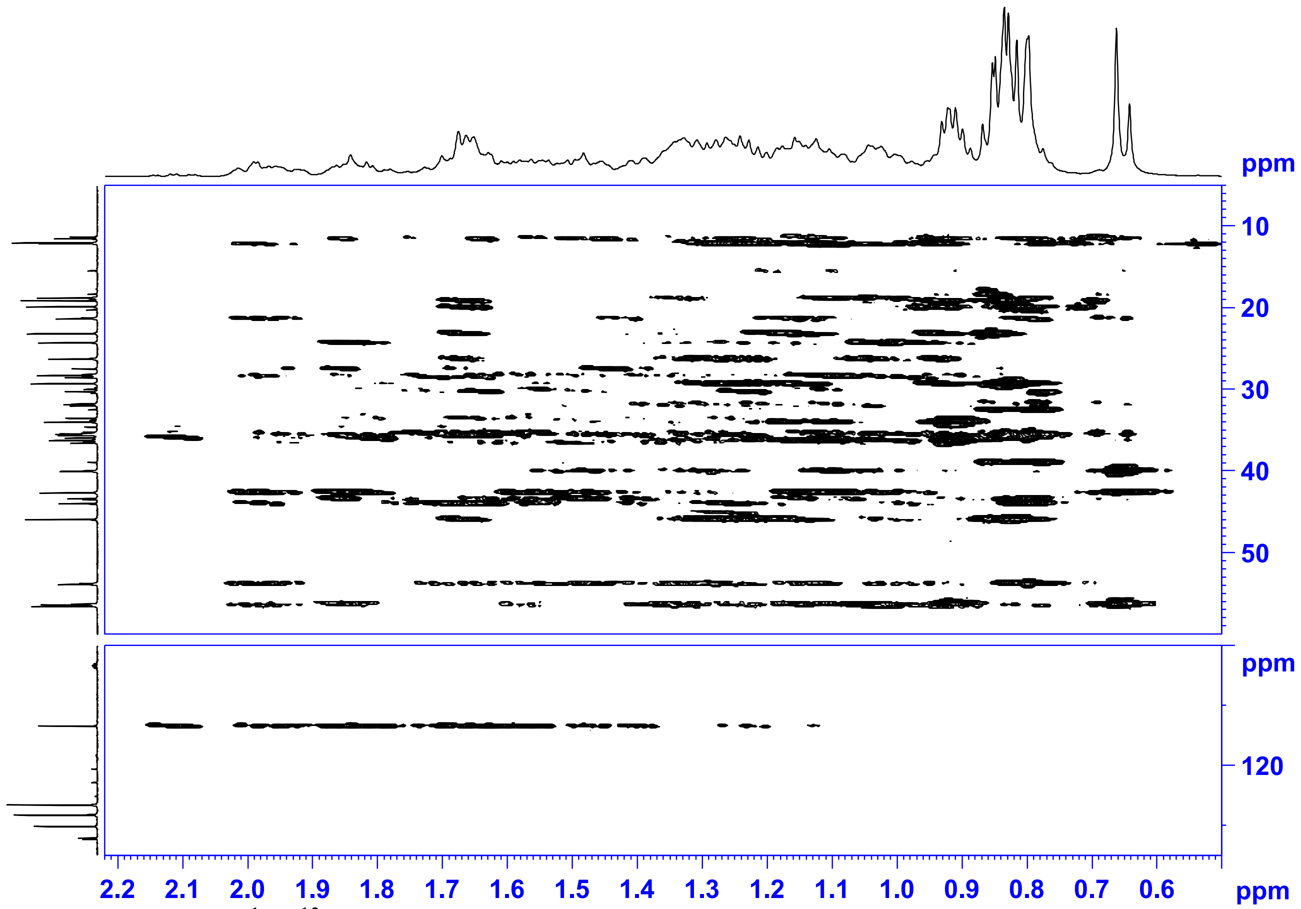

Figure S38. {^{1}H, ^{13}C} HMBC spectrum of the initial four-component diastereomeric mixture of compound **4**.

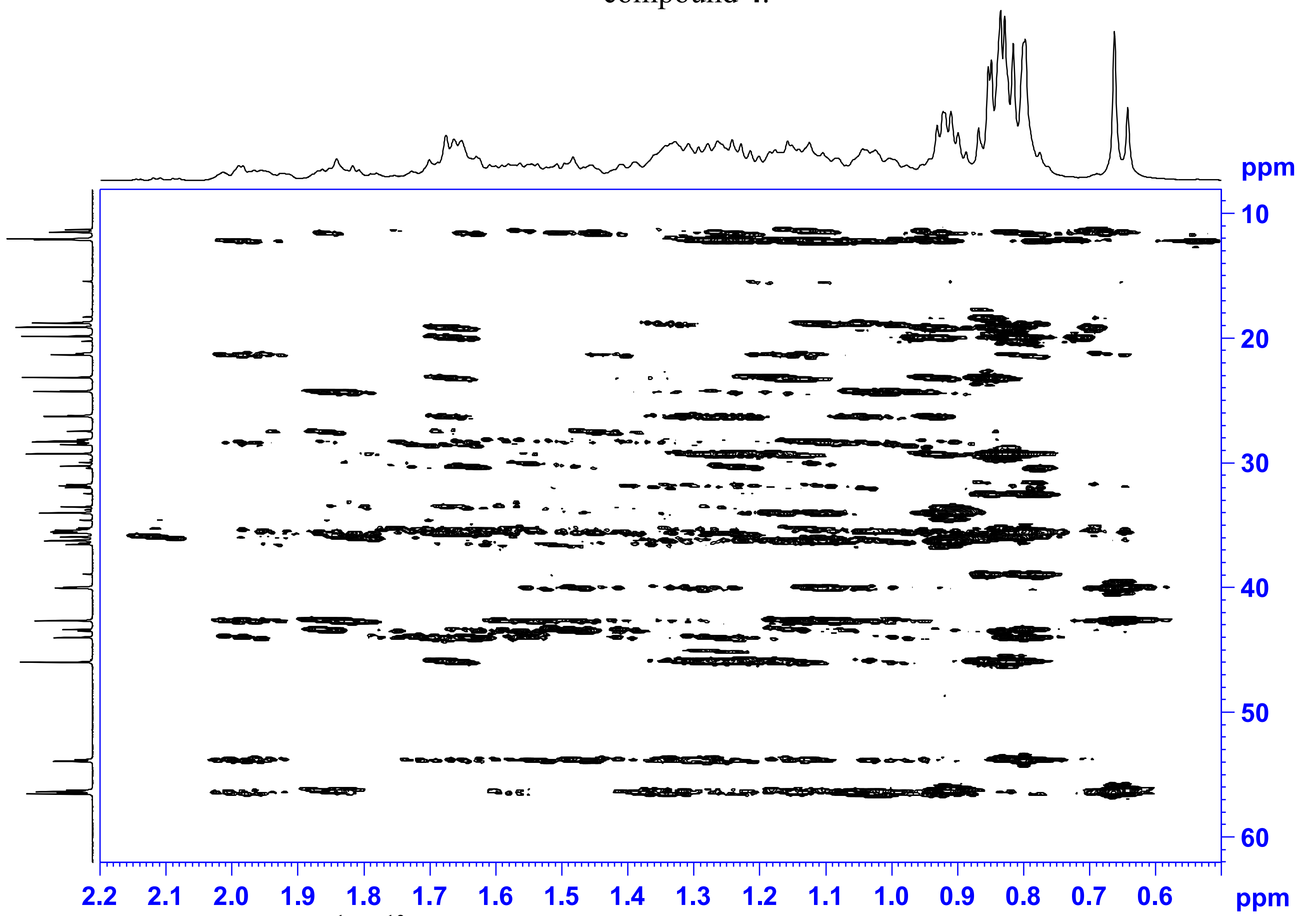

Figure S39. Expanded {^{1}H, ^{13}C} HMBC spectrum of the initial four-component diastereomeric mixture of compound **4**.

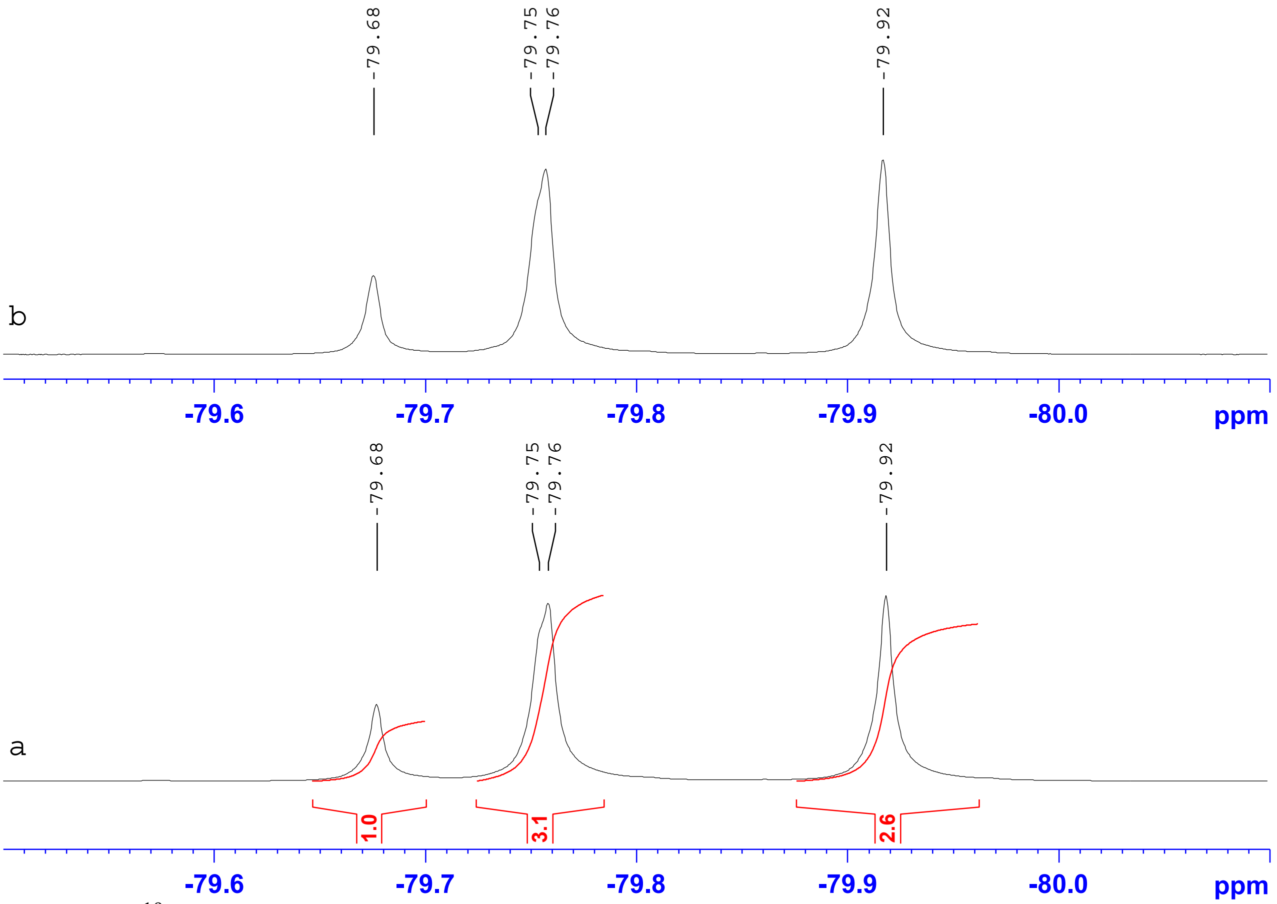

Figure S40. ^{19}F NMR spectra of the initial four-component diastereomeric mixture of compound **4**: (a) $^{19}F\{^1H\}$, (b) ^{19}F.

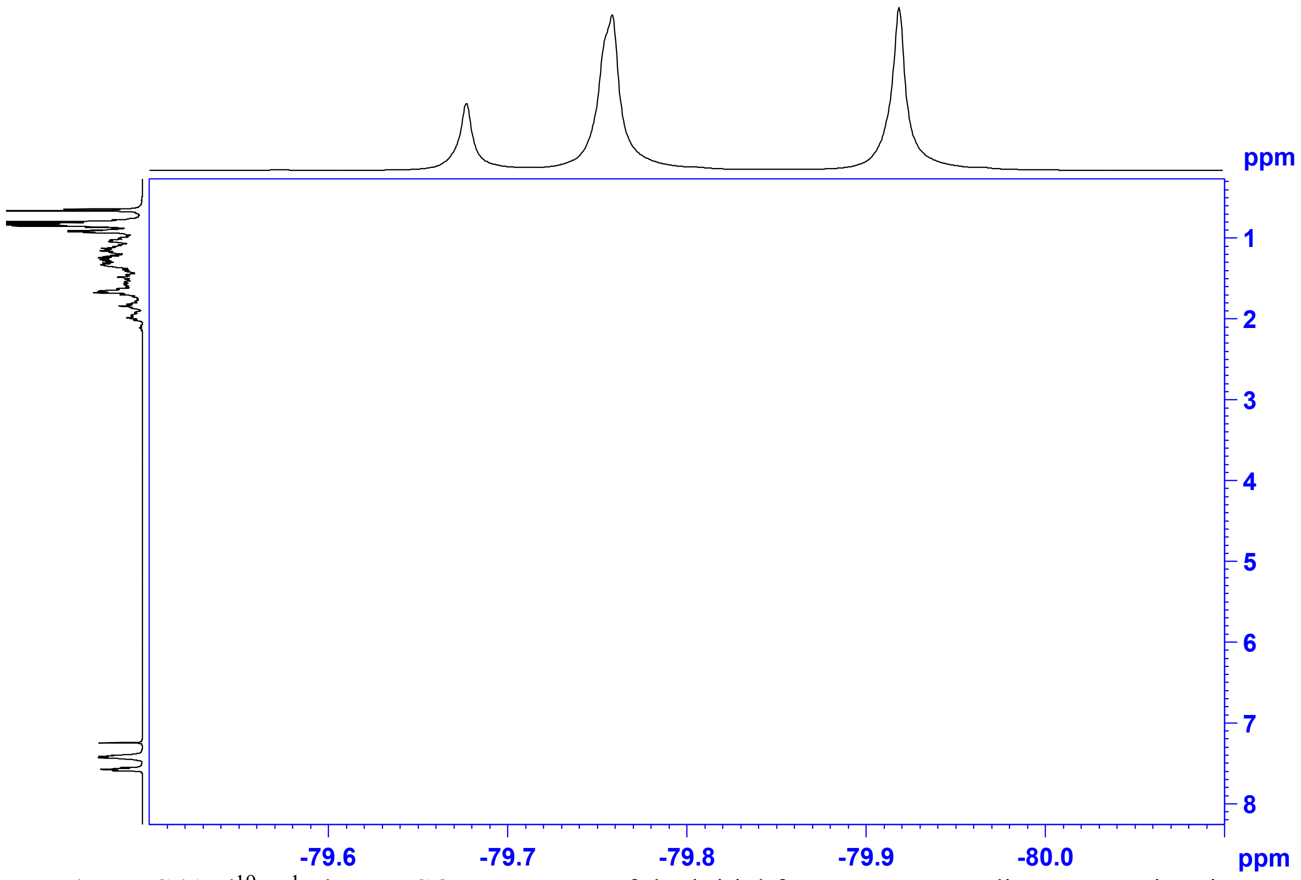

Figure S41. $\{^{19}F, ^1H\}$ HETCOR spectrum of the initial four-component diastereomeric mixture of compound **4**.

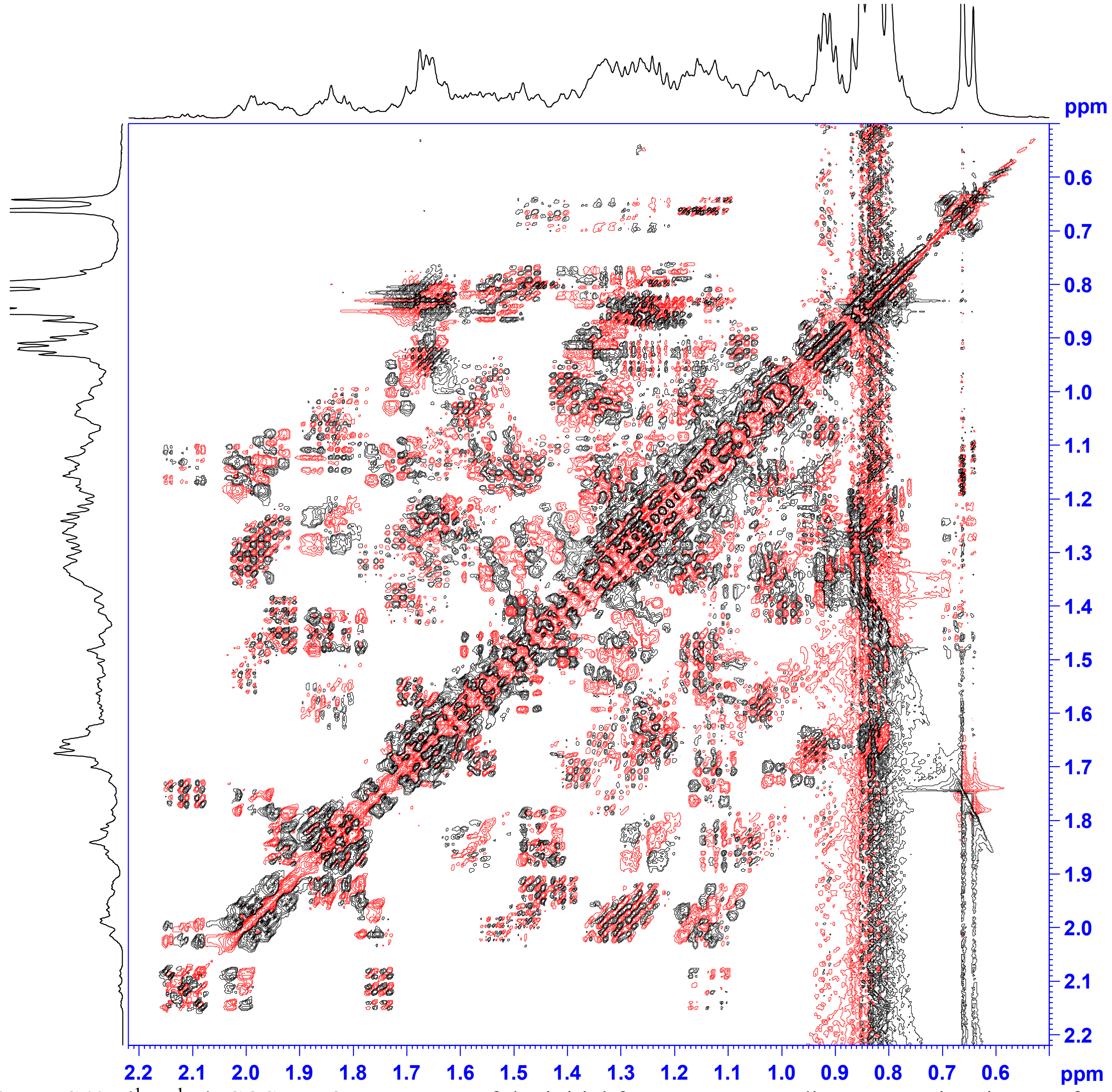

Figure S42. {^1H, ^1H} COSY-DQF spectrum of the initial four-component diastereomeric mixture of compound **4**.

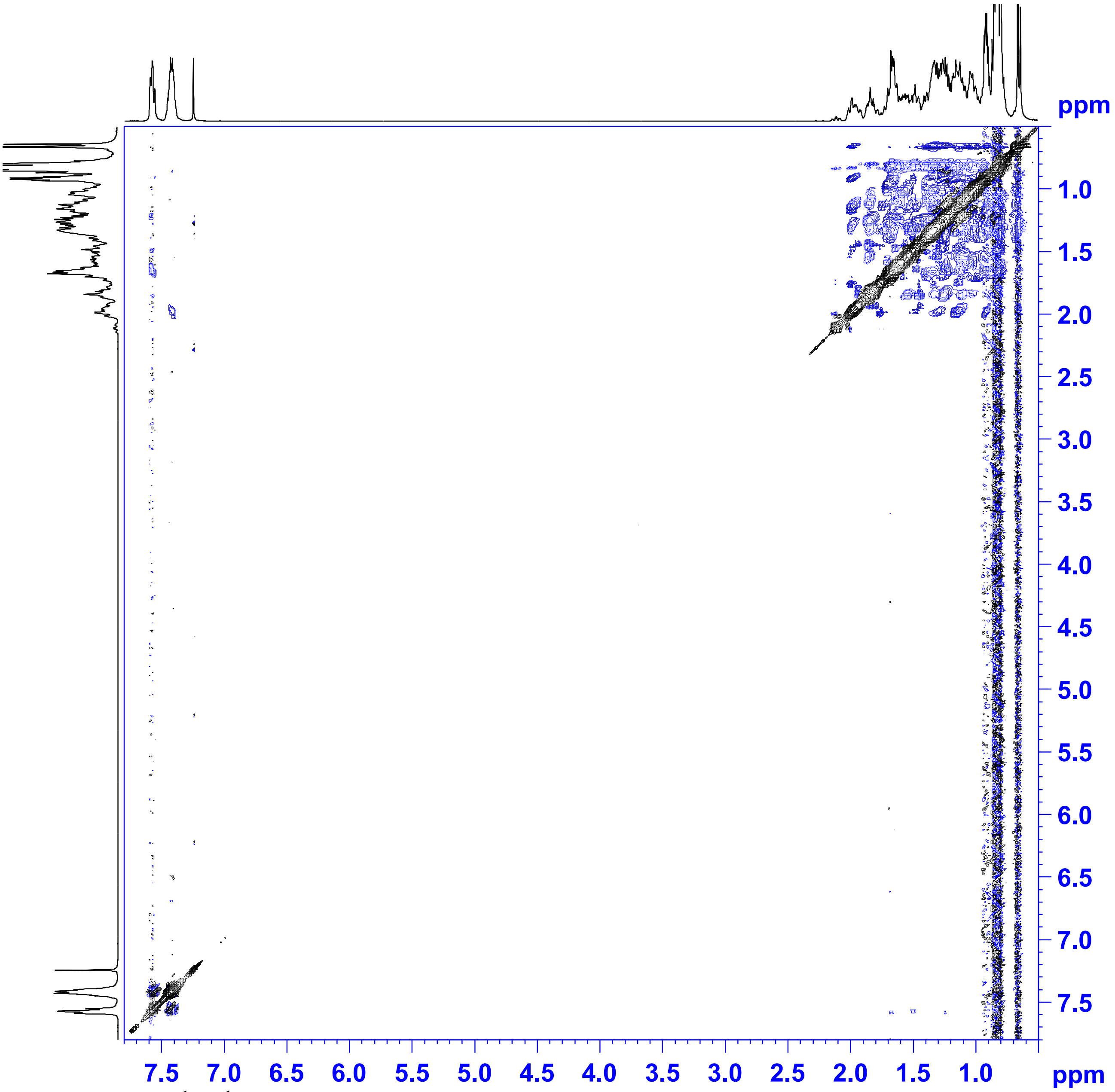

Figure S43. {^{1}H, ^{1}H} NOESY spectrum of the initial four-component diastereomeric mixture of compound **4**.

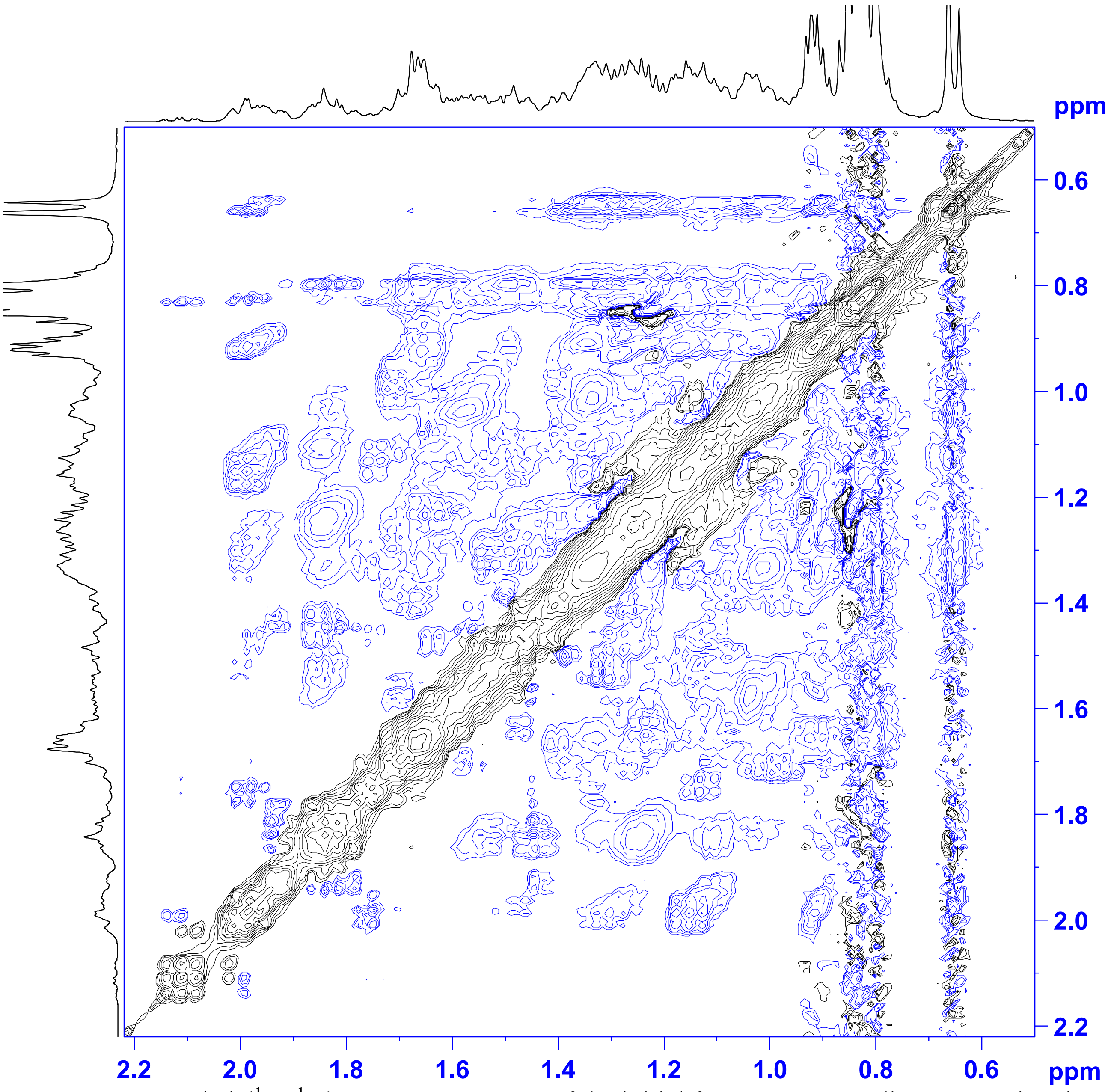

Figure S44. Expanded {^{1}H, ^{1}H} NOESY spectrum of the initial four-component diastereomeric mixture of compound **4**.

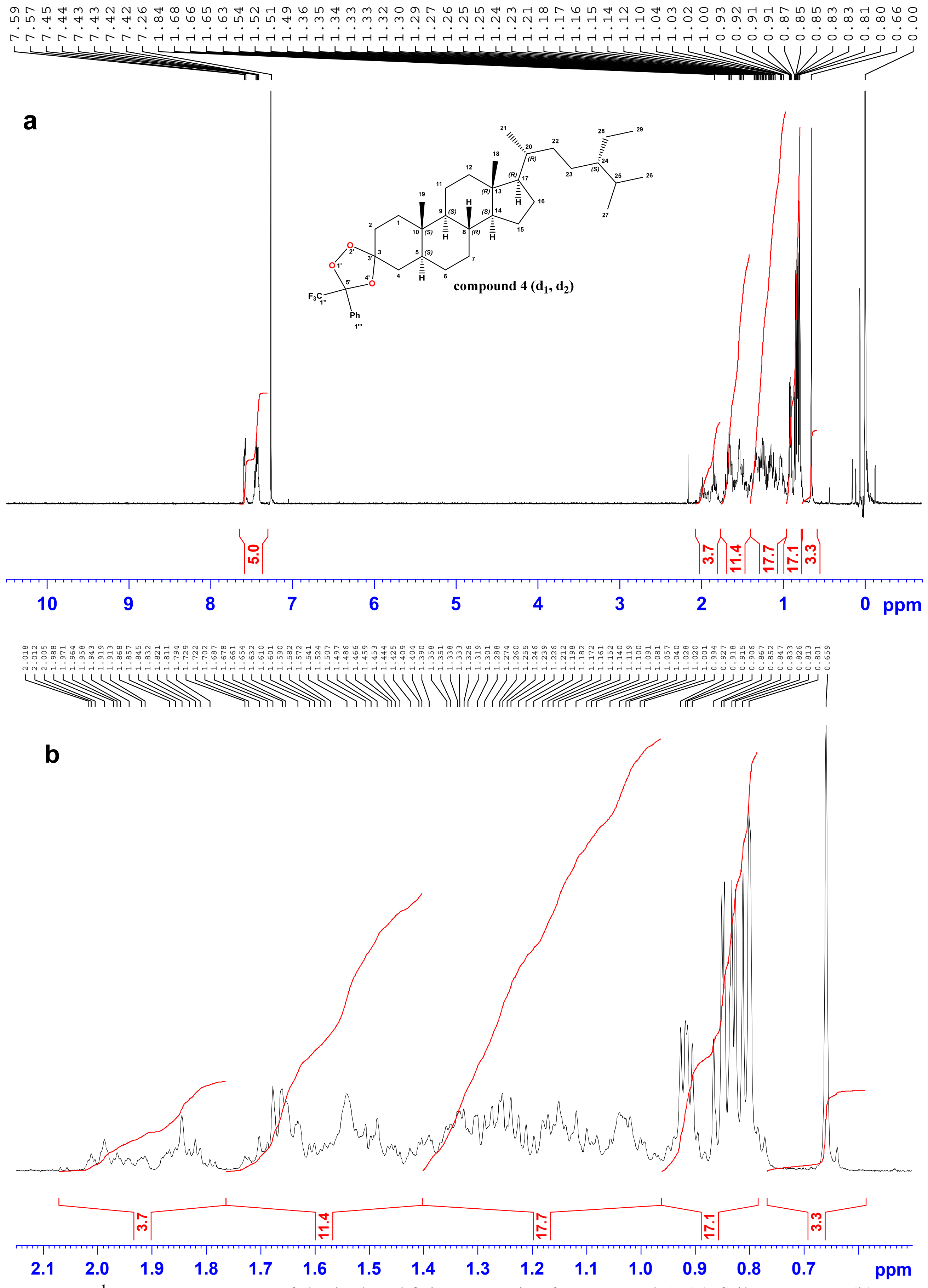

Figure S45. ^{1}H NMR spectrum of the isolated β-isomer pair of compound **4**: (a) full spectrum; (b) expanded region.

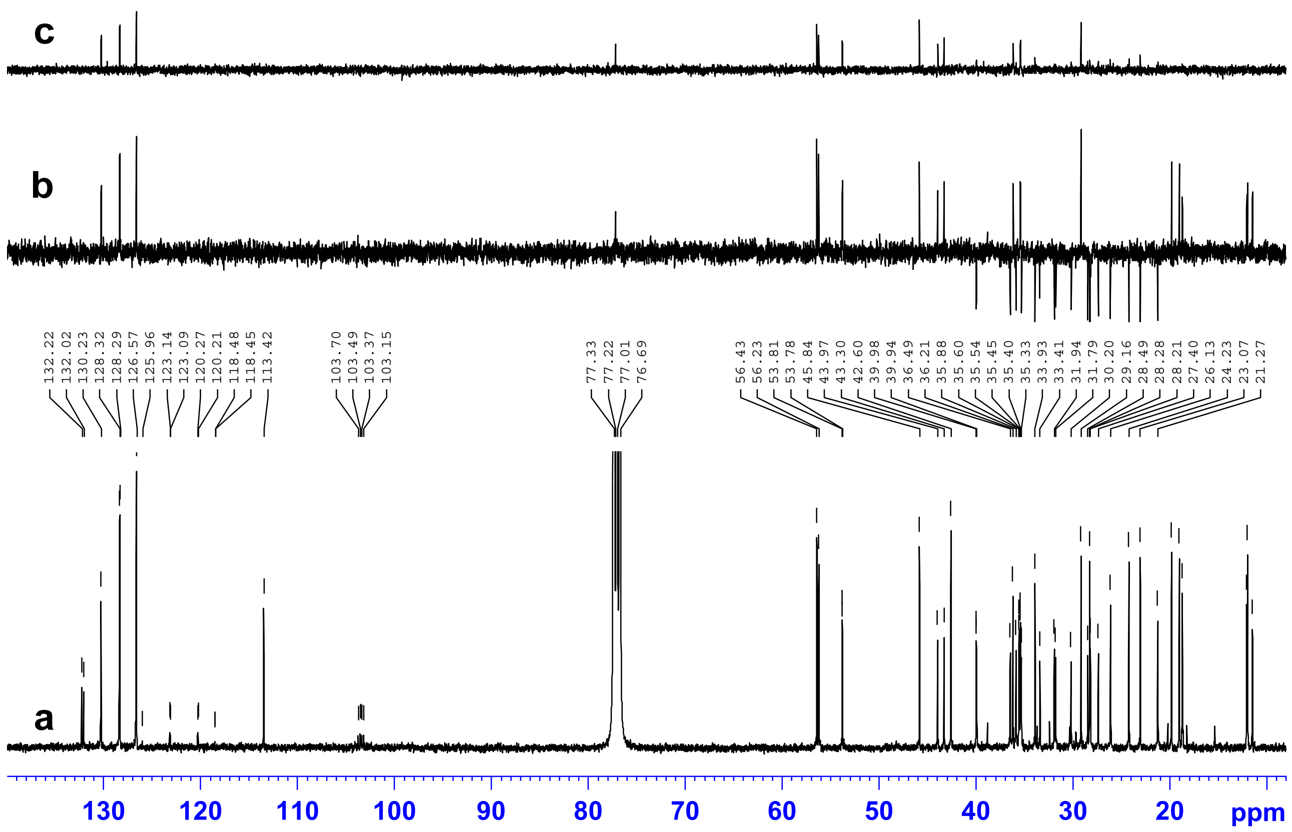

Figure S46. ^{13}C NMR spectra of the isolated β-isomer pair of compound **4**: (a) ^{13}C{^{1}H}, (b) DEPT-135, (c) DEPT-90.

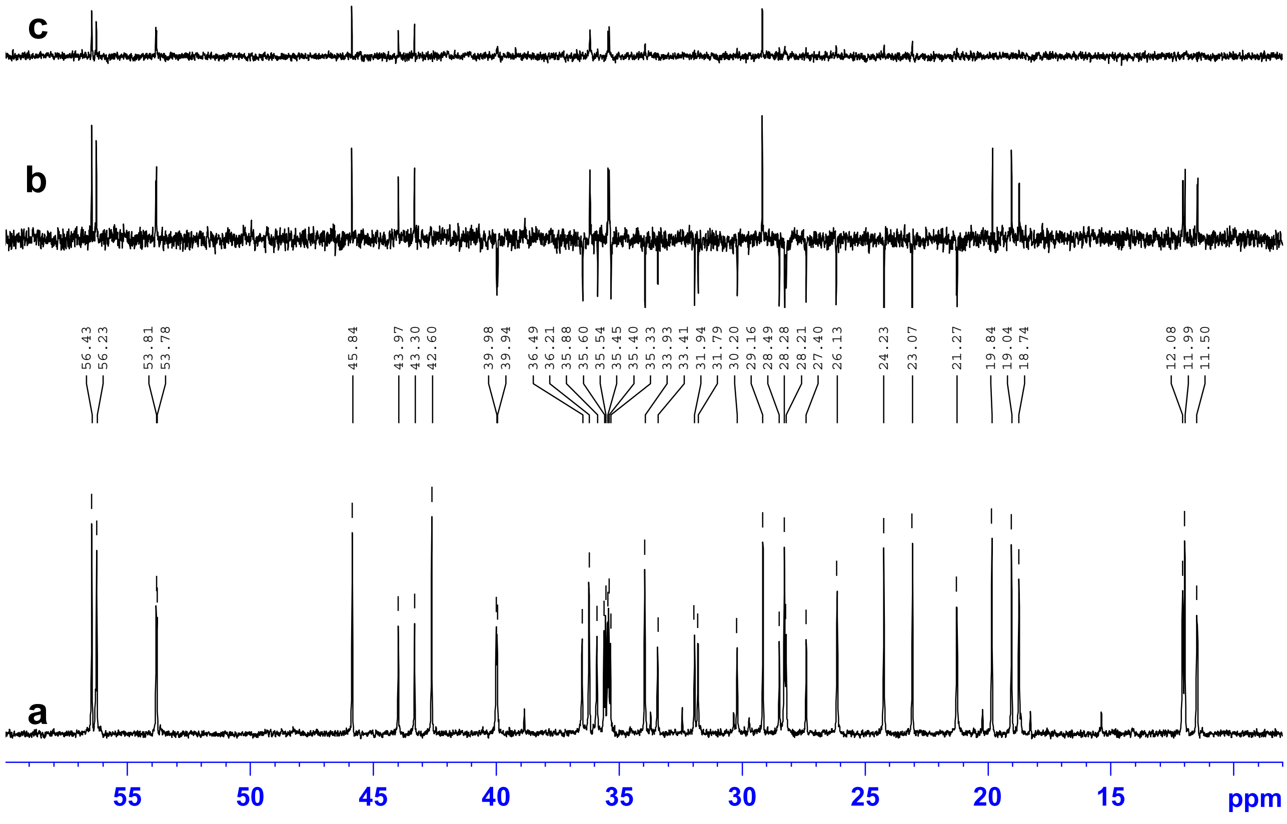

Figure S47. Expanded ^{13}C NMR spectra of the isolated β-isomer pair of compound **4**: (a) ^{13}C{^{1}H}, (b) DEPT-135, (c) DEPT-90.

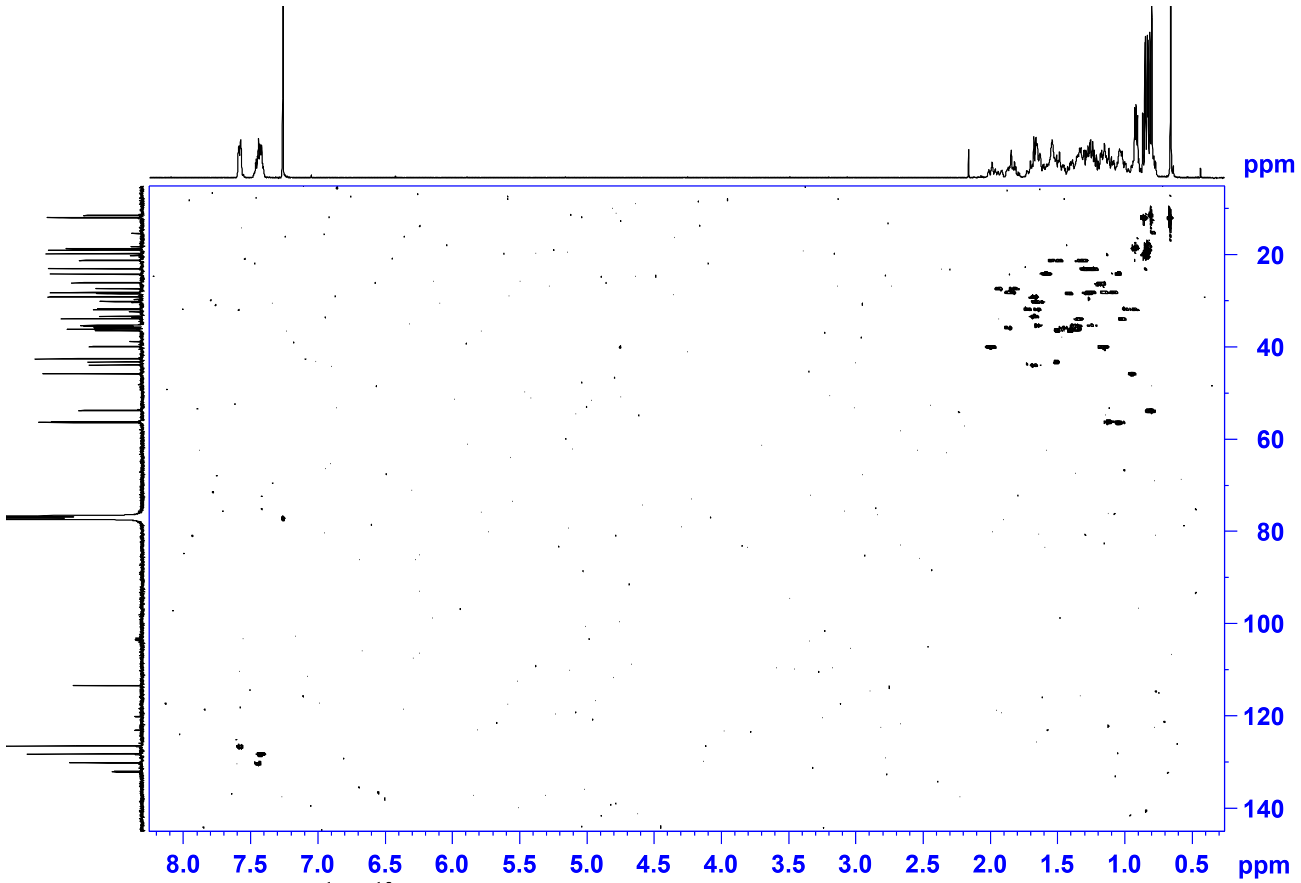

Figure S48. {^{1}H, ^{13}C} HSQC spectrum of the isolated β-isomer pair of compound **4**.

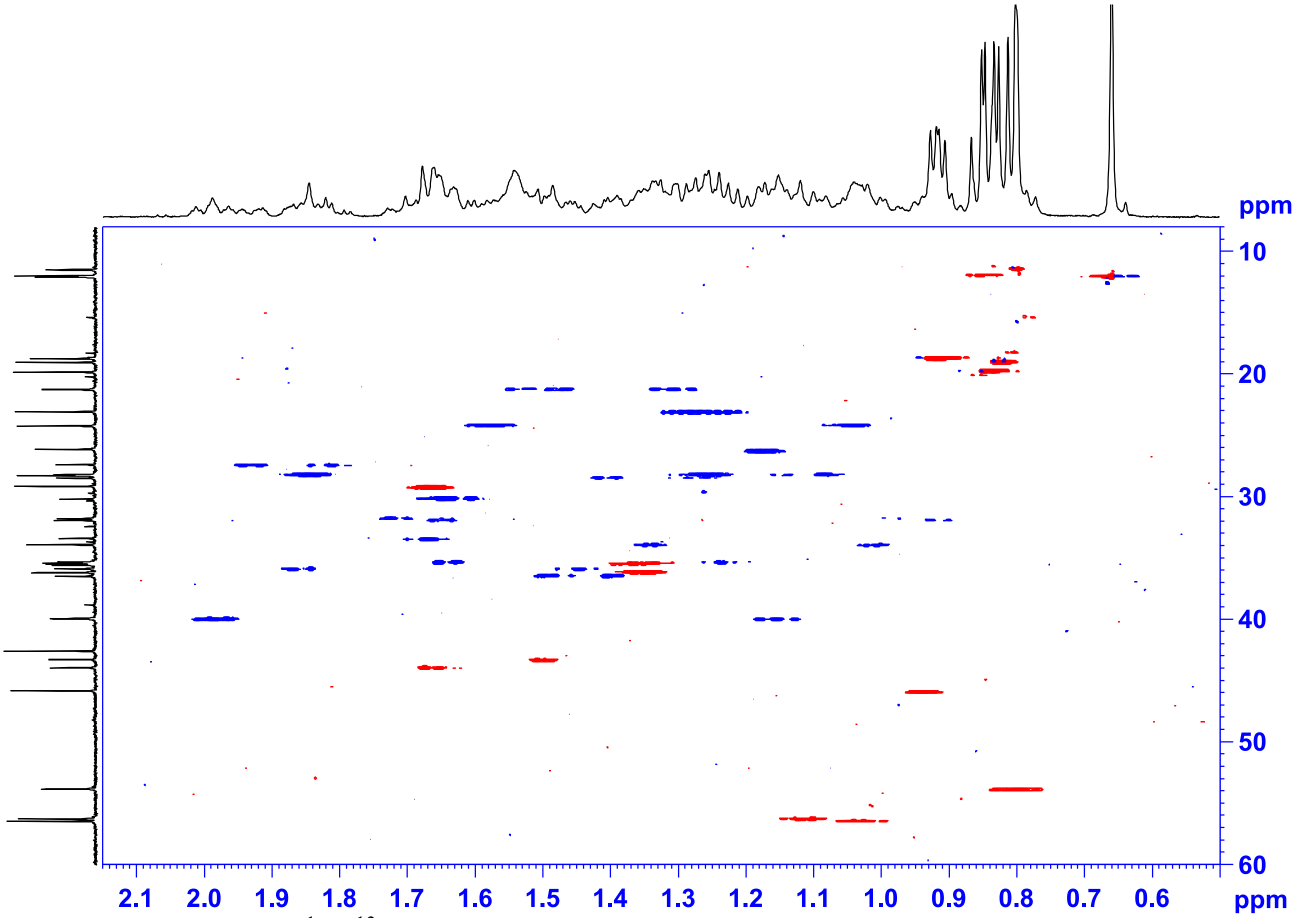

Figure S49. {^{1}H, ^{13}C} HSQCed spectrum of the isolated β-isomer pair of compound **4**.

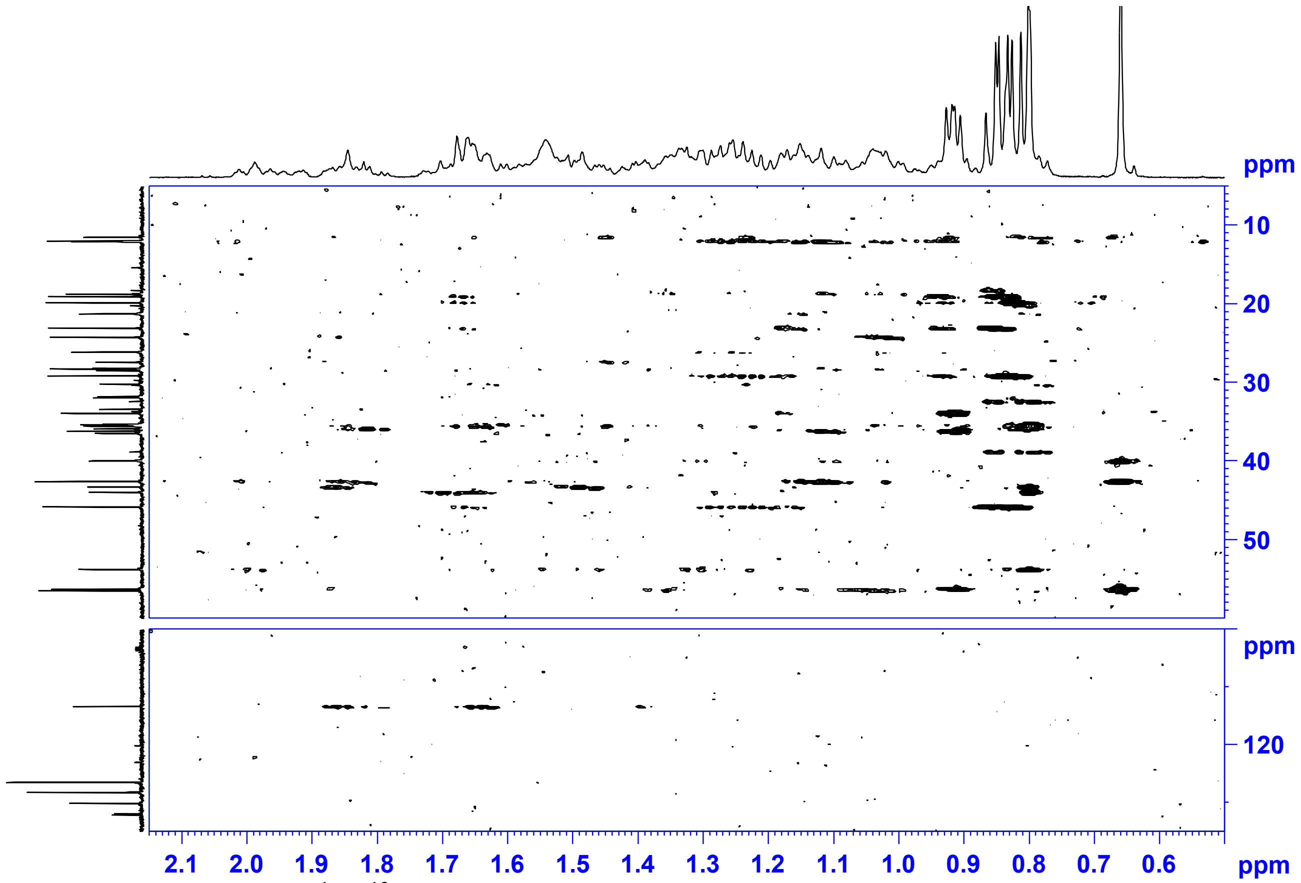

Figure S50. {¹H, ¹³C} HMBC spectrum of the isolated β-isomer pair of compound **4**.

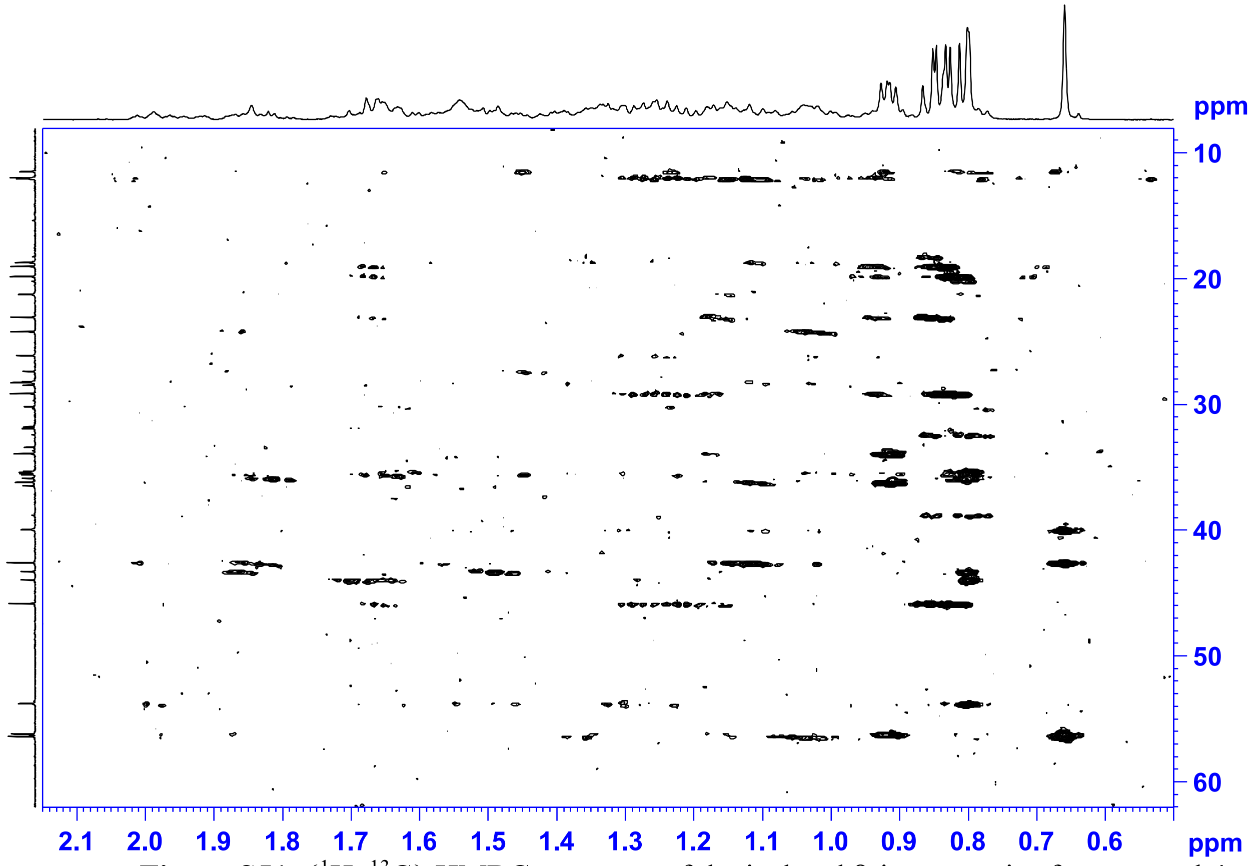

Figure S51. {¹H, ¹³C} HMBC spectrum of the isolated β-isomer pair of compound **4**.

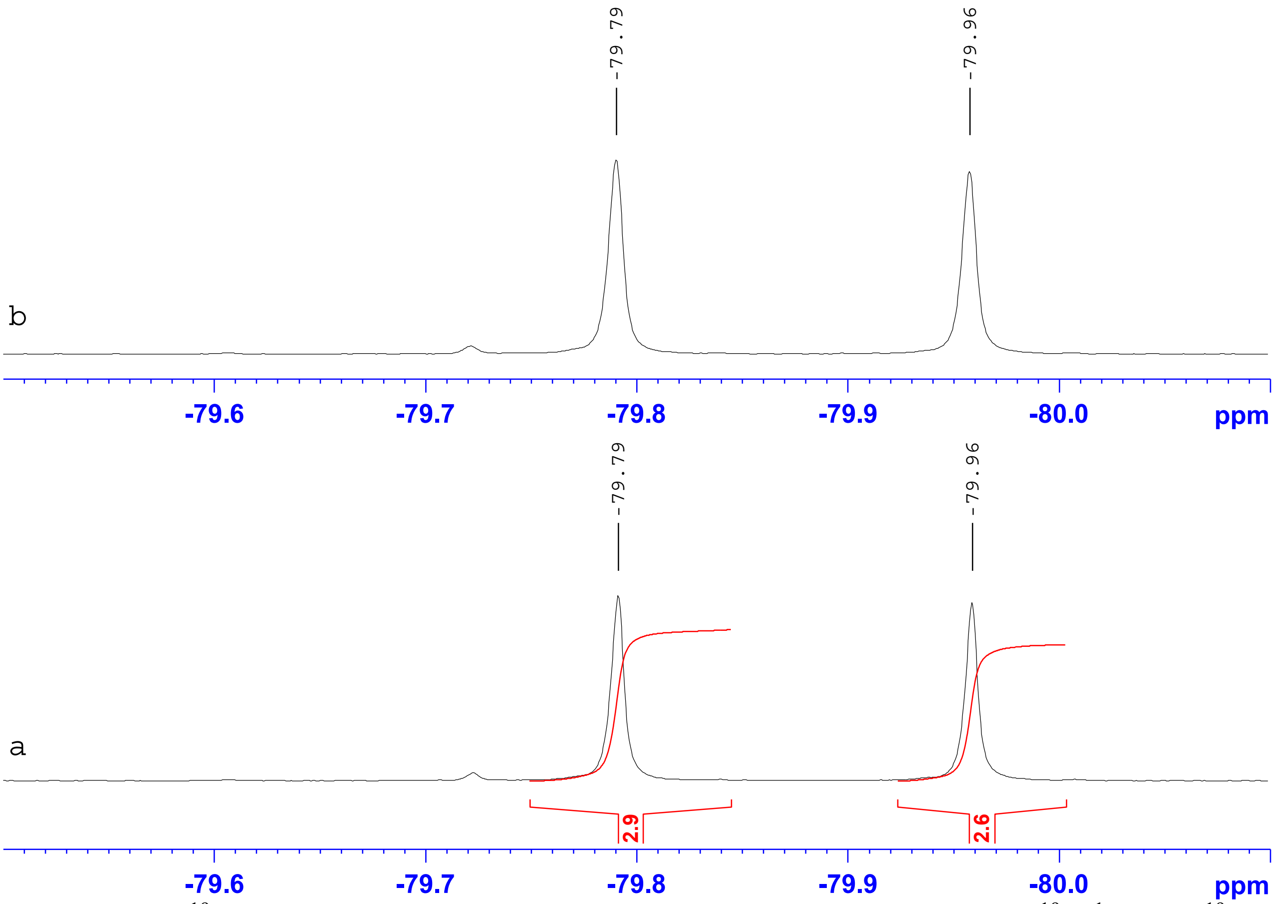

Figure S52. ^{19}F NMR spectra of the isolated β-isomer pair of compound **4**: (a) ^{19}F{^{1}H}, (b) ^{19}F.

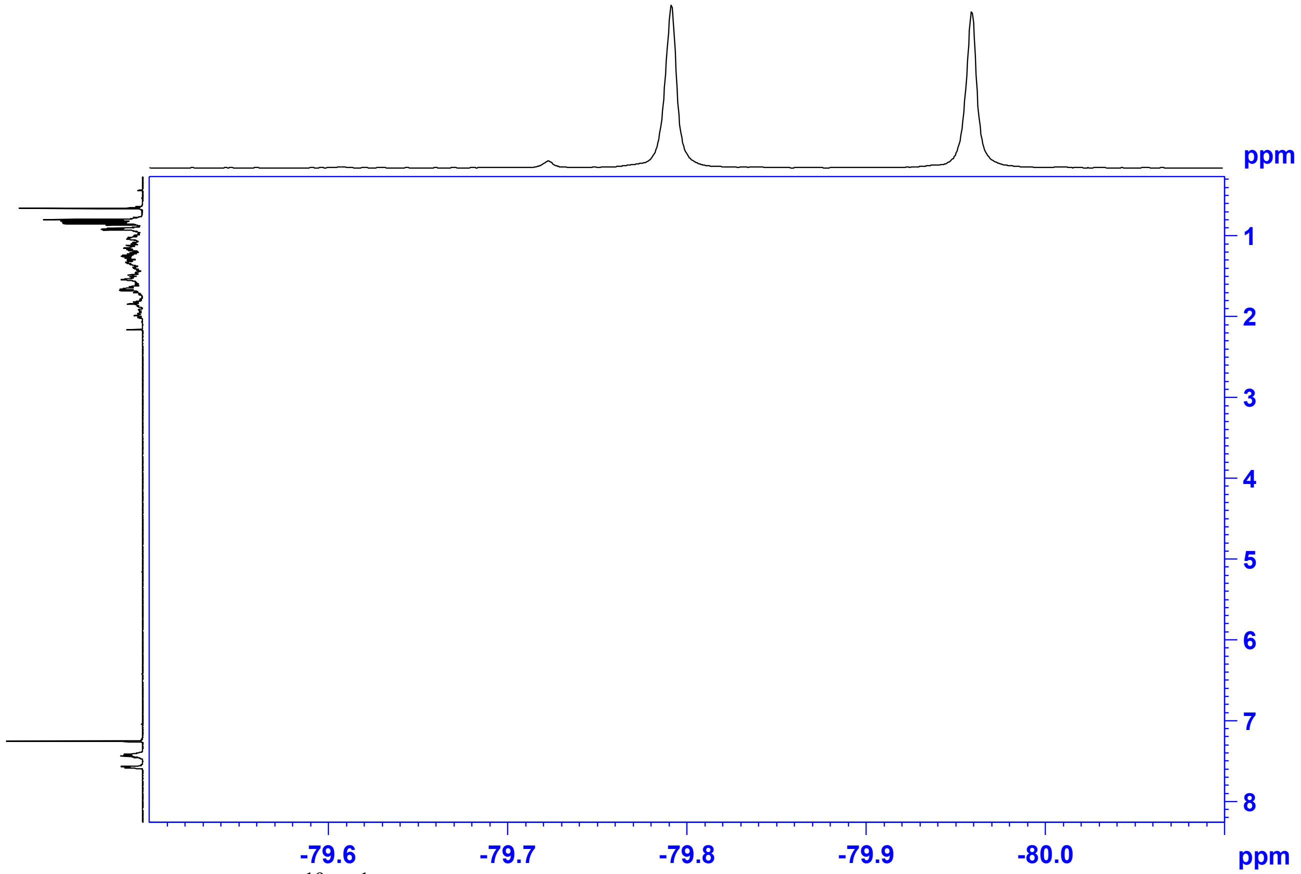

Figure S53. {^{19}F, ^{1}H} HETCOR spectrum of the isolated β-isomer pair of compound **4**.

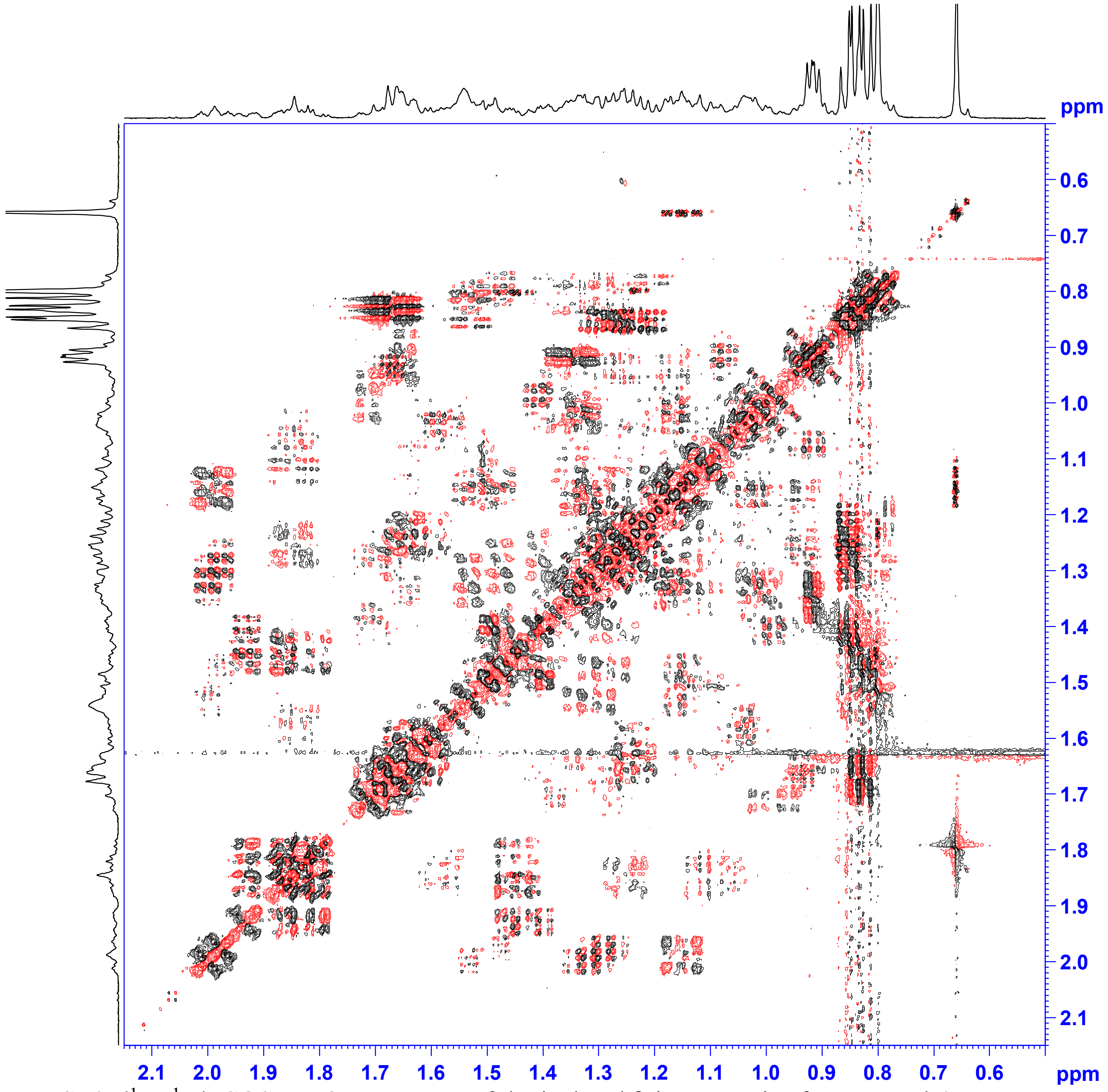

Figure S54. {^{1}H, ^{1}H} COSY-DQF spectrum of the isolated β-isomer pair of compound **4**.

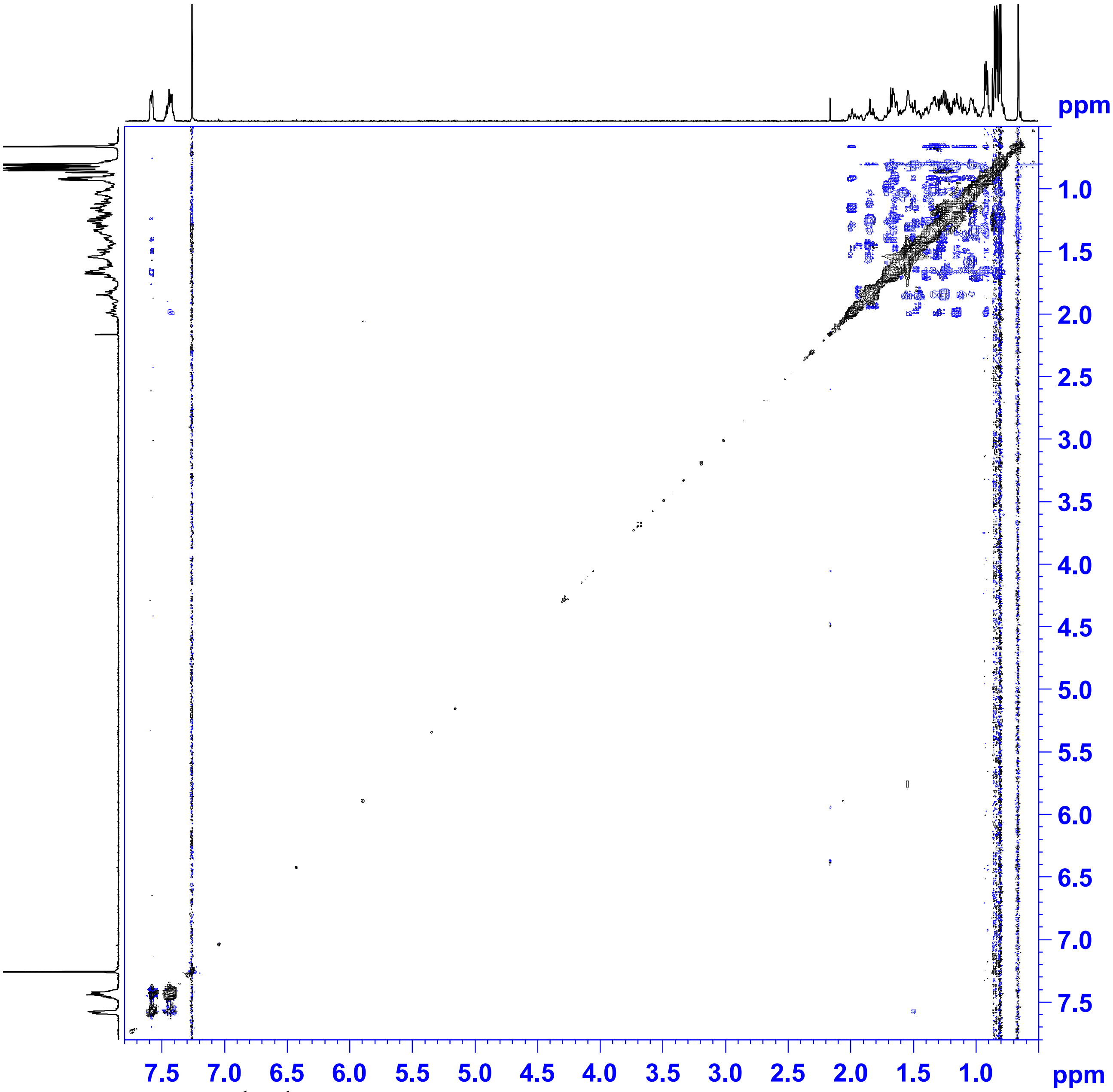

Figure S55. {^{1}H, ^{1}H} NOESY spectrum of the isolated β-isomer pair of compound **4**.

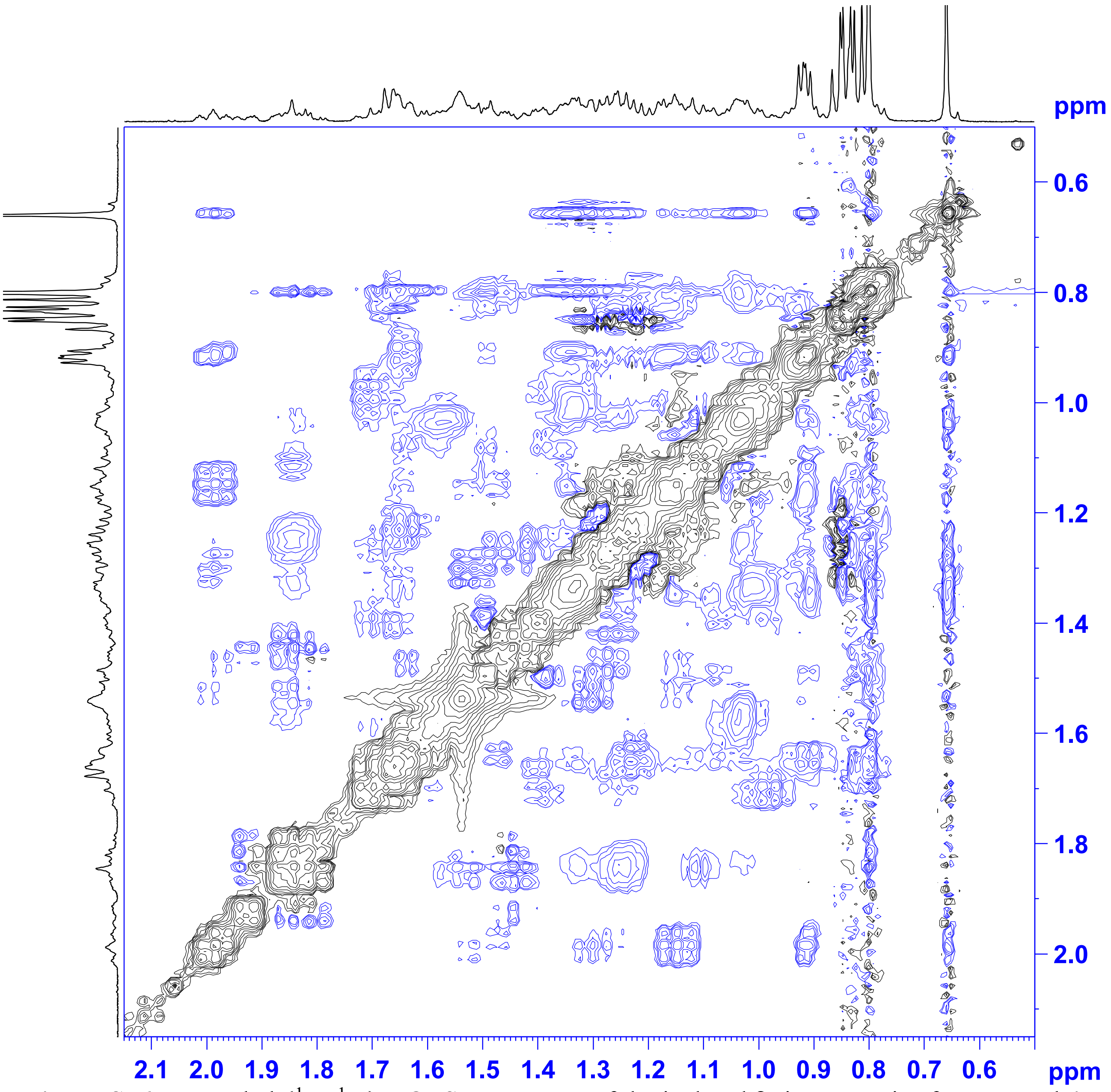

Figure S56. Expanded {^{1}H, ^{1}H} NOESY spectrum of the isolated β -isomer pair of compound **4**.

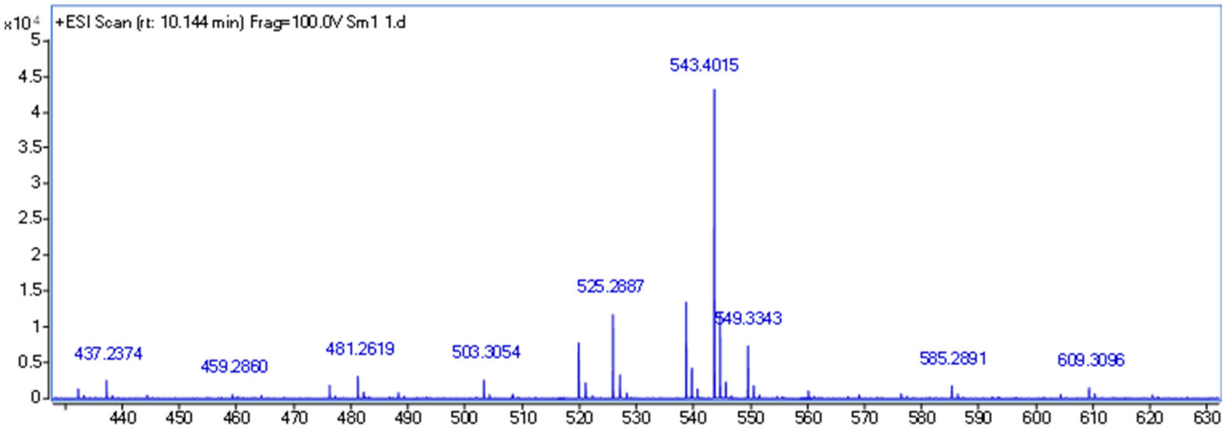

Figure S57. HRMS data of compound **3d$_1$**, **3d$_3$**

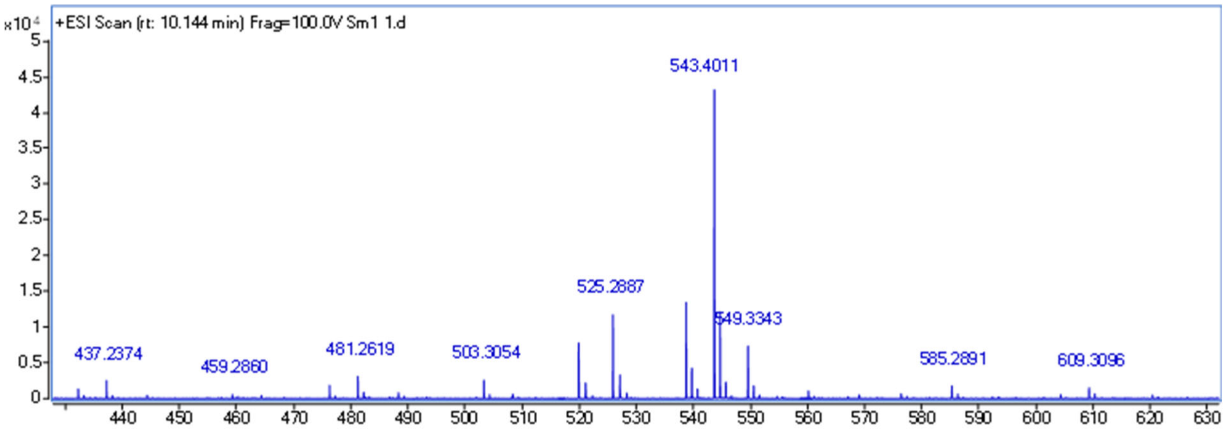

Figure S58. HRMS data of compound **3d$_2$**, **3d$_4$**
